# Dual-Pathway Strategy for Click-Type Functionalization and Programmable Polymer Deconstruction

**DOI:** 10.1021/acs.macromol.6c00380

**Published:** 2026-02-18

**Authors:** Ivan O. Levkovsky, Lucca Trachsel, Hironobu Murata, Krzysztof Matyjaszewski

**Affiliations:** Department of Chemistry, 6612 Carnegie Mellon University, Pittsburgh, Pennsylvania 15213, United States

## Abstract

Developing polymeric materials that combine precise, modular functionalization with programmed backbone degradability remains an outstanding challenge in macromolecular engineering. Herein, we present a molecular design strategy that integrates orthogonal postpolymerization modification with selective, stimulus-responsive backbone degradability within a single macromolecular platform. The ring-opening polymerization of 1,2-dithiolanes introduces cleavable disulfide linkages into polymer backbones, providing a powerful route to degradable materials under biologically relevant reducing conditions. By incorporating a β-triketone (TK) moiety into an α-lipoic-acid-derived 1,2-dithiolane, we synthesized triketone–lipoic acid (TKLA), a dual-functional monomer that combines click-type, catalyst-free amine ligation with programmed backbone degradability. Leveraging photoinduced electron/energy-transfer reversible addition–fragmentation chain-transfer (PET-RAFT) copolymerization of TKLA with acrylate and acrylamide monomers, we accessed well-defined copolymers containing both pendant TK groups and disulfide-rich backbones in a single synthetic step. Under mild conditions, TK-bearing copolymers react quantitatively with a broad scope of amines to form β,β′-diketoenamines (DKEs), enabling modular installation of hydrophilic, hydrophobic, charged, and bioactive substituents. Importantly, these DKE moieties are not static but participate in associative transamination, allowing dynamic exchange and reconfiguration of installed functionalities. Meanwhile, the disulfide-containing backbones fragment cleanly and selectively under reducing environments, affording controlled deconstruction while preserving, or transforming, the appended side-chain chemistry. Overall, the integration of efficient, chemoselective β-triketone–amine condensation with LA-based degradability within a single monomer framework establishes a molecular design strategy for constructing functional, recyclable, and stimuli-responsive polymer architectures.

## Graphical abstract

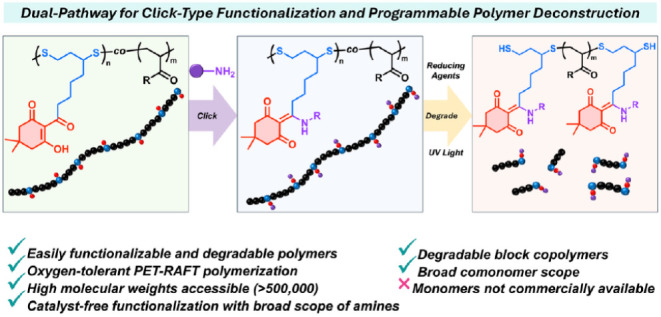

## Introduction

Designing polymeric materials that can be both precisely functionalized and efficiently deconstructed remains a central challenge in sustainable macromolecular engineering.^1^ Advances in introducing degradability into traditionally persistent polymer backbones,^2–5^ together with developments in reversible covalent bonds,^6,7^ have expanded access to materials that can be deliberately dismantled at end-of-use, enabling chemical recycling, programmed disassembly, and stimuli-responsive behavior.^8^ In parallel, postpolymerization modification (PPM) has expanded rapidly as highly efficient and chemoselective organic transformations have been translated from small-molecule chemistry to macromolecular architectures.^9–15^ PPM allows complex macromolecules to be created by transforming reactive pendant groups after polymerization, providing access to (co)­polymers unobtainable through direct polymerization and enabling precise control over properties beyond those of the parent polymer.^16,17^ Yet despite their complementary potential, degradability and PPM are typically treated as orthogonal design axes in the controlled synthesis of degradable polymers, which rarely incorporate robust, orthogonal handles for late-stage functionalization. This constrains opportunities to engineer polymer lifecycles in which structure, function, and end-of-life behavior can be integrated within a single macromolecular platform.

β-Triketones (TKs) have recently emerged as a versatile and underexplored functional motif in polymer and materials science, with reactivity that is exceptionally well-suited for macromolecular diversification. TK moieties undergo spontaneous condensation with primary and secondary amines to form β,β′-diketoenamine (DKE) linkages, releasing water as the sole byproduct and proceeding under mild, catalyst-free conditions. This transformation exhibits hallmarks of click chemistry,^18^ including innocuous byproduct formation, quantitative yields, stoichiometric reactant use, and excellent chemoselectivity,^19–21^ and tolerates a broad range of hydrophilic, hydrophobic, charged, and biologically relevant amines. The high intrinsic reactivity of the TK motif toward amines enables rapid and efficient DKE formation,^22^ while the resulting DKE linkages undergo associative transamination to confer dynamic covalent exchange and network rearrangement under mild conditions.^23^ Drawing inspiration from the dynamic DKE chemistry central to recyclable polydiketoenamine (PDK) plastics,^24–29^ TK-containing vinyl monomers were recently introduced as a click-type platform for modular postpolymerization modification, enabling both efficient late-stage diversification and access to adaptive, reconfigurable polymer materials (Figure 1A).^30^

**Figure 1:**
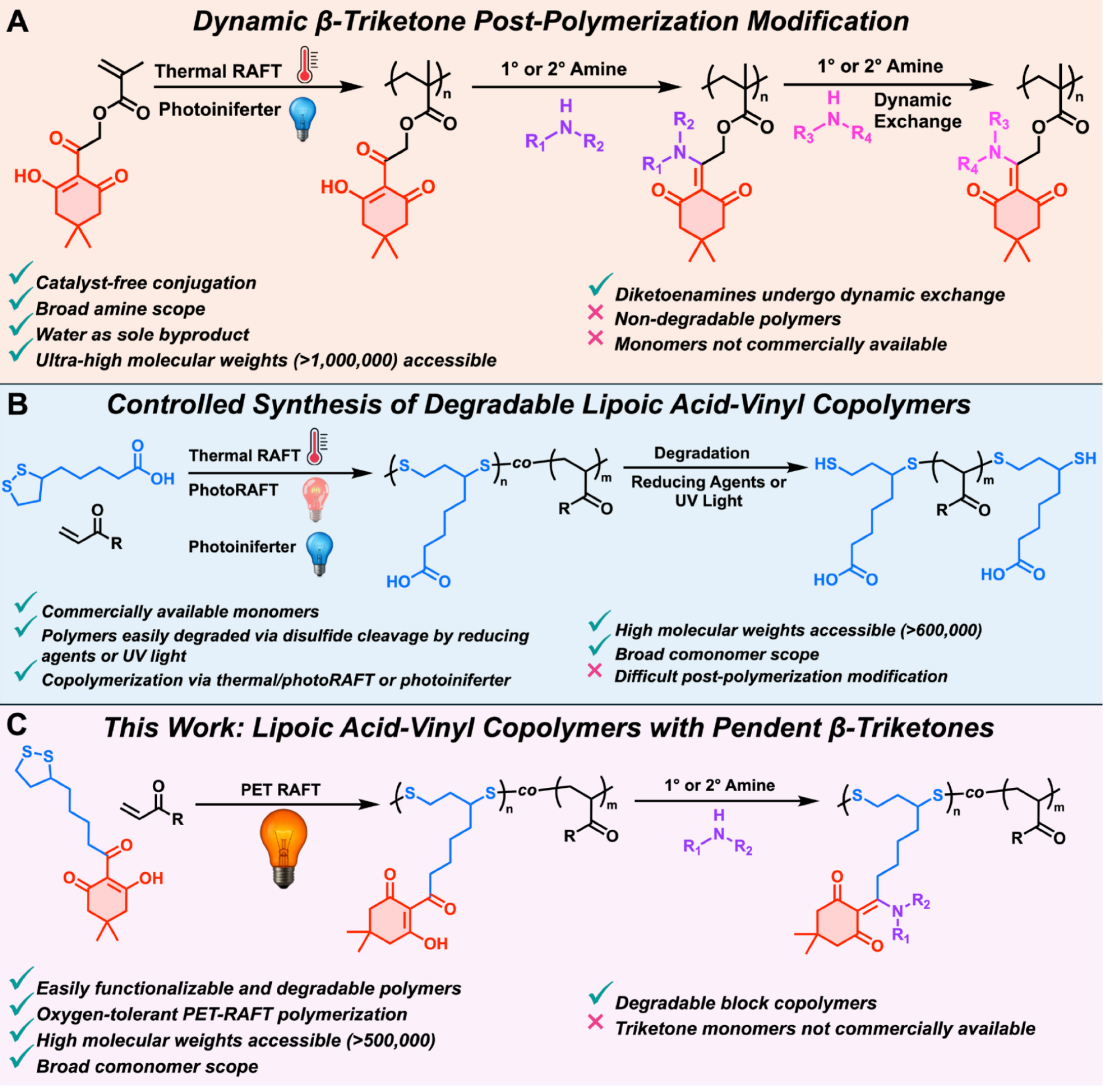
(A) Controlled synthesis of β-triketone (TK)-functionalized vinyl monomers as a click-type platform for modular postpolymerization modification, but on nondegradable polymers.^30^ (B) Controlled copolymerization of lipoic acid and its derivatives via thermal/photo RAFT and photoiniferter techniques, leading to degradable poly­(disulfide)­s with targetable molecular weights and low dispersity (*Đ*), however with difficult postpolymerization modification (PPM).^36,37,39^ (C) This work: copolymerization of triketone-lipoic acid (TKLA) vinyl monomers to obtain copolymers with modular side chains for click-like PPM that is orthogonal to subsequent degradation of the copolymers.

One effective strategy for introducing degradability into vinyl polymer backbones is the incorporation of cleavable disulfide linkages.^31^ α-Lipoic acid (LA), a naturally occurring and readily available 1,2-dithiolane, can undergo radical ring-opening copolymerization with vinylic monomers to generate polymers containing repeating disulfide units that cleave under biologically relevant reducing conditions or upon light exposure.^32^ LA has been incorporated into vinyl polymers using conventional free-radical polymerization, leveraging the radical ring-opening of 1,2-dithiolanes. However, this affords limited control over composition, molecular weight and dispersity (*Đ*).^33–35^ Subsequently, thermal reversible addition–fragmentation chain-transfer (RAFT) and photoiniferter copolymerization with acrylates and acrylamides demonstrated improved molecular weight control, yet remained constrained by oxygen sensitivity, narrow monomer scope, modest degrees of polymerization, and relatively low LA incorporation.^36–38^ Recent advances in oxygen-tolerant, visible-light-driven RAFT polymerization have overcome these constraints, enabling well-defined LA–vinyl copolymers with broad comonomer compatibility, high LA incorporation (up to 68 mol %), and controlled molecular weights with degrees of polymerization exceeding 6,000 and molar masses approaching 700,000 g mol^–1^.^39^ While the pendant carboxylic acid of LA can serve as a functional handle for certain postpolymerization transformations, including conjugation of amines and alcohols^40,41^ or bottlebrushes derived from LA-based poly­(disulfide) backbones,^42^ such modifications require coupling agents, excess reagents, and often generate hazardous byproducts, while lacking the functional group tolerance and chemoselectivity characteristic of click-type ligation.^18^ Consequently, LA-derived backbones still lack modular, orthogonal handles for PPM, restricting their integration into platforms that require both precise functional tailoring and programmable end-of-life degradation.

Taken together, these developments highlight an important opportunity: integrating TK-enabled PPM with LA-based degradability would provide a unified platform in which modular functionalization and programmed disassembly are encoded at the monomer level. However, such integration has not been realized, owing in part to challenges in copolymerizing TK-containing monomers under mild photochemical conditions compatible with disulfide-rich systems.

Herein, we introduce triketone-lipoic acid (TKLA) as a dual-functional monomer that integrates the modular, catalyst-free PPM enabled by TK groups with the programmed degradability imparted by LA-derived disulfides within the backbone. Using photoinduced electron/energy-transfer reversible addition–fragmentation chain-transfer (PET-RAFT) polymerization, we accessed well-defined copolymers bearing pendant TK motifs and backbone disulfide linkages across a broad range of acrylate and acrylamide monomers. These materials undergo quantitative DKE ligation with diverse primary and secondary amines under mild conditions, enabling modular installation of hydrophilic, hydrophobic, charged, and bioactive substituents. Beyond efficient ligation, the resulting DKE linkages retain their intrinsic dynamic covalent behavior, undergoing associative transamination that enables exchange and remodeling of installed functionalities. In parallel, the disulfide-rich backbones undergo rapid, selective cleavage under reducing conditions, allowing controlled deconstruction while preserving—or transforming—the appended side-chain functionality. By uniting orthogonal PPM with backbone degradability at the monomer level, TKLA establishes a molecular design strategy for engineering polymer lifecycles and provides a versatile platform for sustainable materials, functional coatings, and stimuli-responsive systems.

## Results and Discussion

### Synthesis of Degradable Polymers Bearing Pendant Triketones

We first synthesized a 1,2-dithiolane-containing TKLA monomer in one step from commercially available DL-α-lipoic acid (LA) and dimedone (Scheme 1). TKLA was characterized via ^1^H and ^13^C NMR spectroscopy (Figures S1 and S2). ^1^H NMR spectroscopy revealed the presence of a highly deshielded enolic proton at 18.2 ppm characteristic of TKs,^43^ confirming successful TK installation. The TKLA monomer was obtained as a highly viscous oil that was prone to autopolymerization over time, consistent with other reported liquid LA derivatives.^42,44^ The autopolymerization was likely further exacerbated by the acidic enolic proton of the triketone moiety,^45^ leading to initiation of dithiolane ring-opening via a cationic mechanism under concentrated conditions.^46,47^ To mitigate this, stock solutions of TKLA were prepared in 1,4-dioxane or DMSO, and stored frozen at −20 °C to enhance the shelf life of the monomer.

**Scheme 1:**
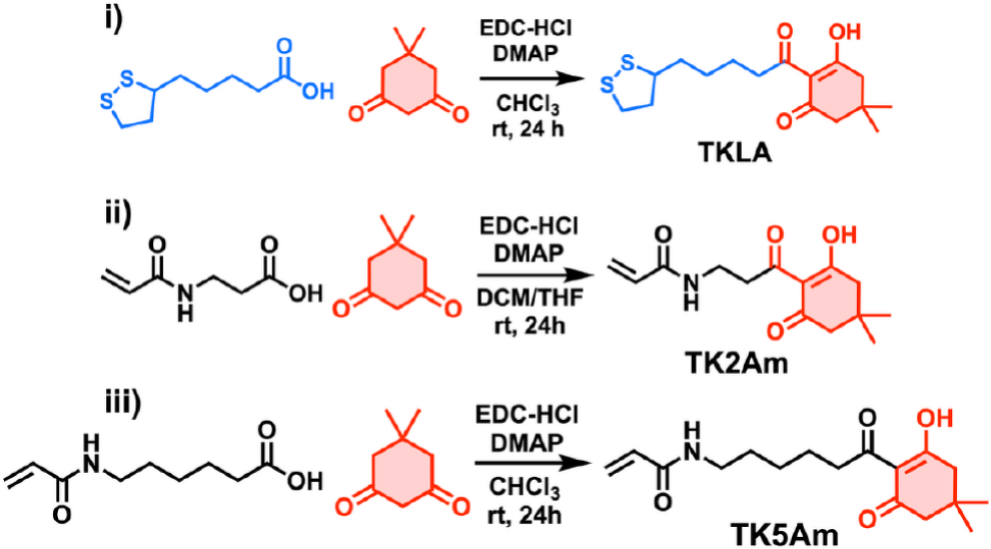
Synthesis of Monomers Containing *β*-Triketone (TK) Moiety^a^

a (i) Triketone lipoic acid (TKLA), (ii) triketone ethylene acrylamide (TK2Am), and (iii) triketone pentamethylene acrylamide (TK5Am).

We next sought to copolymerize TKLA in a controlled fashion with vinyl monomers using a photoinduced reversible-deactivation radical polymerization (RDRP) technique.^48–52^ Photochemical approaches are attractive because they can operate under oxygen-tolerant conditions and at ambient or subambient temperatures, as demonstrated in our previously reported methylene blue-mediated red light driven photoRAFT system,^53^ which enabled higher LA incorporation and higher degrees of polymerization in LA-vinyl copolymers than thermally initiated RAFT.^39^ However, the limited solvent scope of this system made it incompatible with hydrophobic TKLA and other nonpolar vinyl monomers. Therefore, we chose a PET-RAFT approach using zinc tetraphenylporphyrin (ZnTPP) as the photocatalyst, retaining the ability to use longer wavelengths (λ > 500 nm) that are compatible with 1,2-dithiolanes, operational simplicity, and oxygen tolerance.^54^ We began by exploring the copolymerization of TKLA with *N,N*-dimethylacrylamide (DMA) under green light irradiation (λ_max_ = 525 nm, 14 mW cm^–2^) in DMSO, using 2-(dodecylthiocarbonothioylthio)-2-methylpropanoic acid (DDMAT) as the chain transfer agent (CTA), at a ratio of [MA]/[LA]/[DDMAT] = 150/50/1, a total monomer concentration [M]_tot_ = 3.0 M, and 0.01 equiv ZnTPP relative to the CTA. The polymerization was carried out in a capped glass vial without prior deoxygenation. Under these conditions, the polymerization reached 86% conversion for DMA and 87% conversion for TKLA in 2 h, determined by ^1^H NMR spectroscopy (Entry 1, Table 1). The resulting P­(TKLA-*co*-DMA) copolymer exhibited low dispersity (*Đ* = 1.25), and its molar mass as determined by size-exclusion chromatography (SEC) relative to poly­(methyl methacrylate) (PMMA) standards (*M*_n,app_ = 29,900 g mol^–1^) closely agreed with the theoretical number-average molar mass calculated from monomer conversion by ^1^H NMR spectroscopy (*M*_n,theory_ = 27,100 g mol^–1^).

**Table 1:** Optimization of Polymerization Conditions^a^

Entry	Solvent	Time (h)	λ_max_ (nm)	ZnTPP (equiv)	Comonomer	[M]_tot_ (M)	TKLA feed (mol %)	Vinyl Conv. (%)^d^	TKLA Conv.(%)^d^	*M*_n,theory_ (kg mol^–1^)^f^	*M*_n,app_ (kg mol^–1^)^g^	*Đ* ^g^
1	DMSO	2.0	525	0.01	DMA	3.0	25	86	87	27.1	29.9	1.25
2	DMSO/DMF	4.0	525	0.02	DMA	3.0	25	63	74	21.4	19.7	1.26
3	DMSO/Dioxane	2.0	525	0.02	DMA	2.0	25	74	n.d.^e^	n.d.^e^	20.7	1.22
4^b^	DMSO/Dioxane^b^	2.0	525	0.02	DMA	2.0	25	77	n.d.^e^	n.d.^e^	19.6	1.23
5	DMSO	3.0	595	0.01	DMA	3.0	15	93	92	24.7	24.2	1.14
6^c^	DMSO	2.0	595	0.01	DMA	3.0	25	81	85	25.9	27.0	1.21
7^d^	DMSO	2.0	595	0.01	DMA	3.0	50	55	70	28.4	16.9	1.52
8	DMSO	2.0	595	0.01	TK2Am	3.0	15	55	69	31.5	24.0	1.32
9	DMSO	2.0	595	0.01	TK2Am	3.0	20	49	69	28.9	24.4	1.30
10	DMSO	2.0	595	0.01	TK5Am	3.0	20	31	55	21.6	15.8	1.38
11	DMSO^c^	2.0	595	0.01	TK5Am	3.0	15	49	77	33.2	25.0	1.27

a Reaction conditions: [DMA]/[TKLA]/[DDMAT]/[ZnTPP] = x/y/1/0.01–0.02, irradiated under 525 nm (14 mW cm^–2^) or 595 nm (7.0 mW cm^–2^) LED.

b 2-(Butylthiocarbonothioylthio)­propanoic acid BTPA was used as the chain transfer agent (CTA).

c Polymerization was run after deoxygenation via sparging with argon for 15 min and sealing the vial with a septum.

d Monomer conversion was determined by ^1^H NMR spectroscopy.

e TKLA conversion was not determined due to overlap of TKLA monomer peak with 1,4-dioxane (δ = 3.7 ppm).

f Theoretical number-average molar masses (*M*_n,theory_) were determined from the monomer conversion.

g Apparent number-average molar masses (*M*_n,app_) and dispersity (*Đ*) were determined by SEC using DMF + 50 mM LiBr relative to PMMA standards.

The copolymerization proceeded similarly in solvent mixtures of DMSO and DMF or 1,4-dioxane (50% v/v) (entries 2–4, Table 1), albeit to lower conversions likely due to diminished quenching of singlet oxygen at the lower DMSO content.^55,56^ The copolymerization in DMF proceeded more slowly, taking 4 h to reach comparable conversion. This slower polymerization in DMF has been previously attributed to coordination of the ZnTPP to the tertiary amide of DMF.^54,57^

To further improve control, we attempted to use orange light irradiation at lower intensity (λ_max_ = 595 nm, 7.0 mW cm^–2^), and a decreased feed of TKLA with respect to DMA (15 mol %). Under these conditions ([MA]/[LA]/[DDMAT]/[ZnTPP] = 170/30/1/0.01, total monomer concentration [M]_o_ = 3.0 M), the polymerization reached very high conversions for both comonomers in 3 h (93% for DMA, 92% for TKLA), with excellent agreement between *M*_n,app_ and *M*_n,theory_ and low *Đ* (entry 5, Table 1). 595 nm irradiation was likewise employed at 25 and 50 mol % feed TKLA (Entries 6 and 7, Table 1), however, with some loss over control for the latter copolymerization due to enhanced transfer reactions to backbone disulfide bonds at the high dithiolane feed.^39,58^

To increase the density of functionalizable TK units along the polymer backbones, we synthesized two additional acrylamide-based TK monomers for copolymerization with TKLA, incorporating spacers of different lengths between the amide and triketone functionalities: triketone ethylene acrylamide (TK2Am) and triketone pentamethylene acrylamide (TK5Am) bearing two- and five- carbon spacers, respectively (Scheme 1). Both TK acrylamides were characterized by ^1^H and ^13^C NMR spectroscopy (Figures S3–S8). Copolymerization of TKLA with either TK2Am or TK5Am under PET-RAFT conditions afforded well-defined copolymers with tunable TKLA incorporation, predictable molar masses, and low dispersities (entries 8–11, Table 1). ^1^H NMR spectroscopy of the purified copolymers confirmed quantitative retention of the pendant TK moieties, demonstrating full compatibility of the TK functionality with the PET-RAFT polymerization conditions (Figures S10–S13). Both comonomers enabled high-density incorporation of TK units along disulfide-degradable backbones. Collectively, these results establish a general strategy for accessing highly functional, degradable copolymers that combine dense, click-type functionalization handles with programmable backbone deconstruction. Having established a system for the controlled synthesis of degradable copolymers with TK side chains, we investigated copolymerization kinetics of TKLA with DMA using optimized conditions ([DMA]/[TKLA]/[DDMAT]/[ZnTPP] = 170/30/1/0.01, [M]_tot_ = 3.0 M, using yellow light irradiation (λ_max_ = 595 nm, 7.0 mW cm^–2^). SEC analysis revealed clean, monomodal shifts of traces to lower elution times, indicating progressive, uniform chain growth over the course of polymerization (Figure 2B). Linear pseudo-first-order kinetic plots for both TKLA and DMA indicated constant radical concentration throughout the polymerization (Figure 2C). Moreover, a linear increase in *M*_n,app_ values with total monomer conversion was observed, agreeing closely with *M*_n,theory_ (Figure 2D).

**Figure 2:**
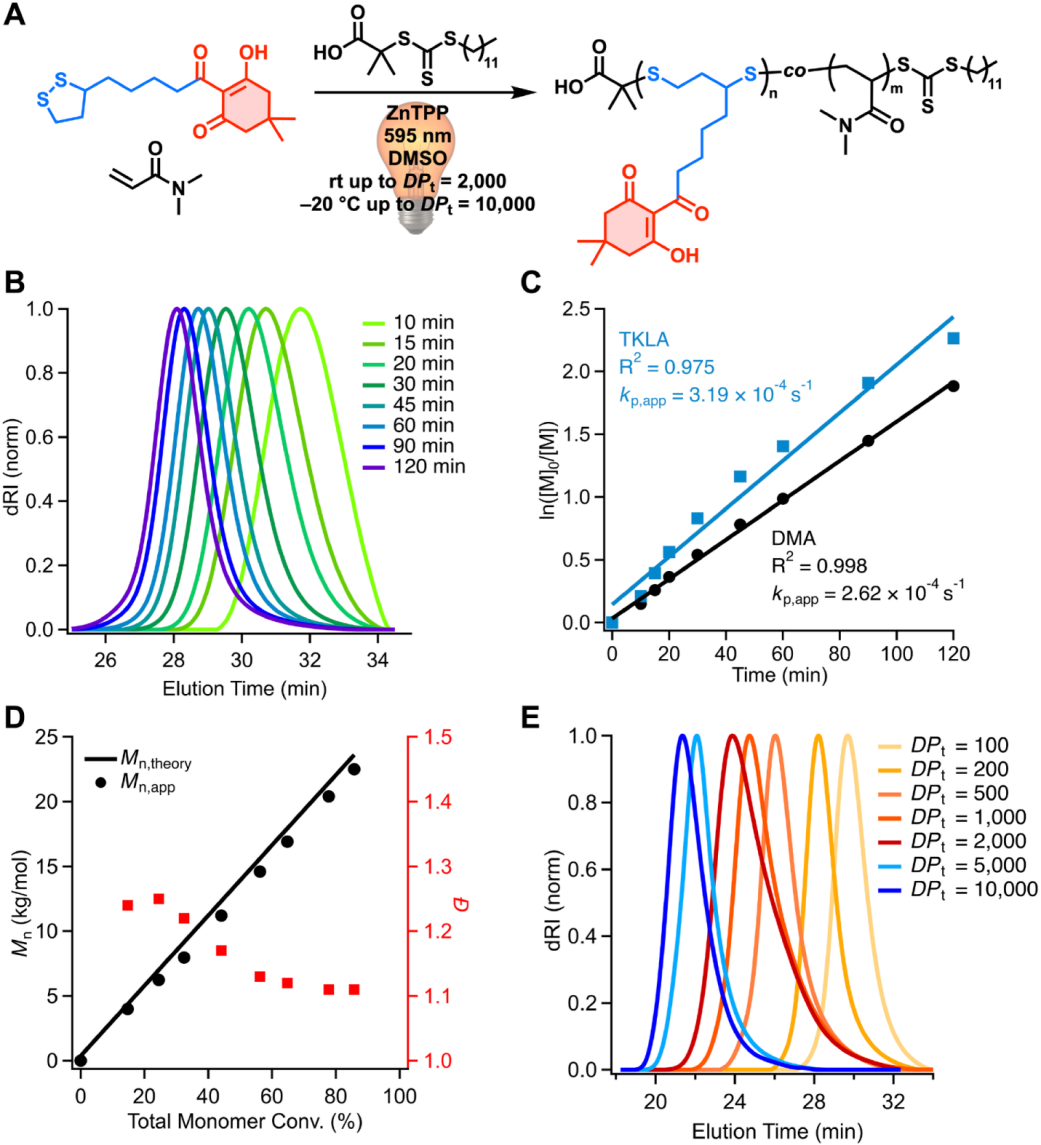
(A) Reaction scheme for the PET-RAFT copolymerization of TKLA with DMA. Reaction conditions: [DMA]/[TKLA/[DDMAT]/[ZnTPP] = 170/30/1/0.01. (B) Evolution of SEC traces of P­(TKLA-*co*-DMA) taken over the course of polymerization, showing time points from 0 to 120 min. (C) Pseudo first-order kinetic plot for TKLA and DMA conversion. Apparent rate constants: *k*_p,app_ = (3.19 ± 0.03) × 10^–4^ s^–1^ for LA (*R* ^2^ = 0.975), and *k*_p,app_ = (2.62 ± 0.02) × 10^–4^ s^–1^ for DMA (*R* ^2^ = 0.998). (D) P­(TKLA*-co*-DMA) *M*_n,app_ versus total monomer conversion showing good agreement with *M*_n,theory_ while maintaining low *Đ*. (E) SEC traces of P­(TKLA*-co*-DMA) with different *DP*_t_ values. Polymerizations targeting DP_t_ = 5,000 and 10,000 were deoxygenated and carried out in the freezer at −20 °C.

Likewise, the copolymerization kinetics of TKLA with TK2Am showcased controlled polymerization behavior (Figure S15).

To demonstrate the ability to obtain degradable TK-functionalized copolymers at predictable molecular weights, 15 mol % TKLA was copolymerized with DMA at targeted degrees of polymerization (*DP*_t_) ranging from 100 to 2,000 by varying the monomer-to-CTA ratio. At these *DP*_t_ values, polymerizations were carried out in capped vials without deoxygenation. The resulting P­(TKLA-*co*-DMA) copolymers showed low to moderate dispersities (*Đ* = 1.14–1.56), with measured molar masses (*M*_n,app_) in agreement with theoretical values and monomodal SEC traces (entries 1–5, Table 2; Figure 2E). At *DP*_t_ = 2,000, conversions of both monomers were markedly reduced relative to lower *DP*_t_ values (entry 5, Figure 2), likely due to the low ZnTPP concentration diminishing the effective oxygen tolerance of the system. Higher molar masses, reaching up to 400,000 g mol^–1^, were achieved at *DP*_t_ = 5,000 and 10,000 using at 10 mol % TKLA feed under slightly modified conditions, including prior deoxygenation, polymerization at −20 °C, and lower light intensity (3.0 mW cm^–2^) (entries 6 and 7, Table 2). Notably, polymerization at reduced temperature led to enhanced TKLA incorporation in the resulting copolymers (Figure S16), consistent with the temperature-dependent equilibrium behavior previously reported for radical polymerizations of 1,2-dithiolanes.^34,39,59^

**Table 2:** Varying Targeted Degrees of Polymerization^a^

Entry	Time (h)	*DP*_t_	[M]_tot_ (M)	TKLA feed (mol %)	Vinyl Conv. (%)^c^	TKLA Conv. (%)^c^	*M*_n,theory_ (kg mol^–1^)^d^	*M*_n,app_ (kg mol^–1^)^e^	*Đ* ^e^
1	2.0	100	3.0	15	88	90	11.8	10.0	1.19
2	3.0	200	3.0	15	93	92	24.7	24.2	1.14
3	1.0	500	3.0	15	71	78	49.1	41.7	1.23
4	1.0	1,000	3.0	15	60	69	84.6	58.3	1.37
5	1.0	2,000	3.0	15	36	48	108	72.3	1.56
6^b^	8.0	5,000	4.0	10^b^	62	78	405	264	1.52
7	8.0	10,000	4.0	10^b^	49	68	661	396	1.67

a Reaction conditions: [DMA]/[TKLA]/[DDMAT]/[ZnTPP] = 0.85x/0.15y/1/0.01, irradiated under 595 nm LED (7.0 mWcm^‑2^) in DMSO solvent at rt.

b Reaction conditions: [DMA]/[TKLA]/[DDMAT]/[ZnTPP] = 0.90x/0.10y/1/0.01, irradiated under 595 nm LED (3.0 mW cm^‑2^) in DMSO solvent at −20 °C, and deoxygenated via sparging with argon for 10 min.

c Monomer conversions determined by ^1^H NMR spectroscopy.

d Theoretical number-average molar masses (*M*_n,theory_) were determined from the monomer conversion.

e Apparent number-average molar masses (*M*_n,app_) and dispersity (*Đ*) were determined by SEC using DMF + 50 mM LiBr relative to PMMA standards.

Together, these results demonstrate controlled access to high molar mass, degradable TK-functionalized copolymers, representing a significant advance in the design of macromolecules with easily modifiable side chains and on-demand degradability.

### Comonomer Scope

TKLA was further copolymerized with a broad range of acrylamide- and acrylate-based monomers, including those bearing polar, nonpolar, charged, zwitterionic, and peptidic functionalities (Figure 3). Acrylates and acrylamides were chosen on account of efficient cross-propagation with the 1,2-dithiolane of LA in RAFT polymerization, in contrast to other vinyl monomers such as methacrylates or styrenics.^33,36,39^ All copolymerizations were performed at 15 mol % TKLA feed, *DP*_t_ = 200, and under yellow light irradiation (λ_max_ = 595 nm, 7.0 mW cm^–2^). Polymerizations were carried out in DMSO, except for zwitterionic carboxybetaine acrylamide (CBAm) and sulfobetaine acrylate (SBA), which required trifluoroethanol (TFE) to solubilize TKLA, the comonomers, and the resulting copolymers. Copolymerization with *n*-butyl acrylate (BA) was carried out in a DMF/DMSO mixture (1:1 v/v) to prevent precipitation of the copolymer observed in 100% DMSO. Furthermore, copolymerizations of TKLA with α-bromoisobutyryl acrylamide (BiBAm), CBAm, TK5Am, BA, and SBA were deoxygenated to enhance monomer conversion at the low DMSO content. In all cases, polymers were obtained with moderate-to-high monomer conversions and molar masses in good agreement with theoretical values (Table S3).

**Figure 3:**
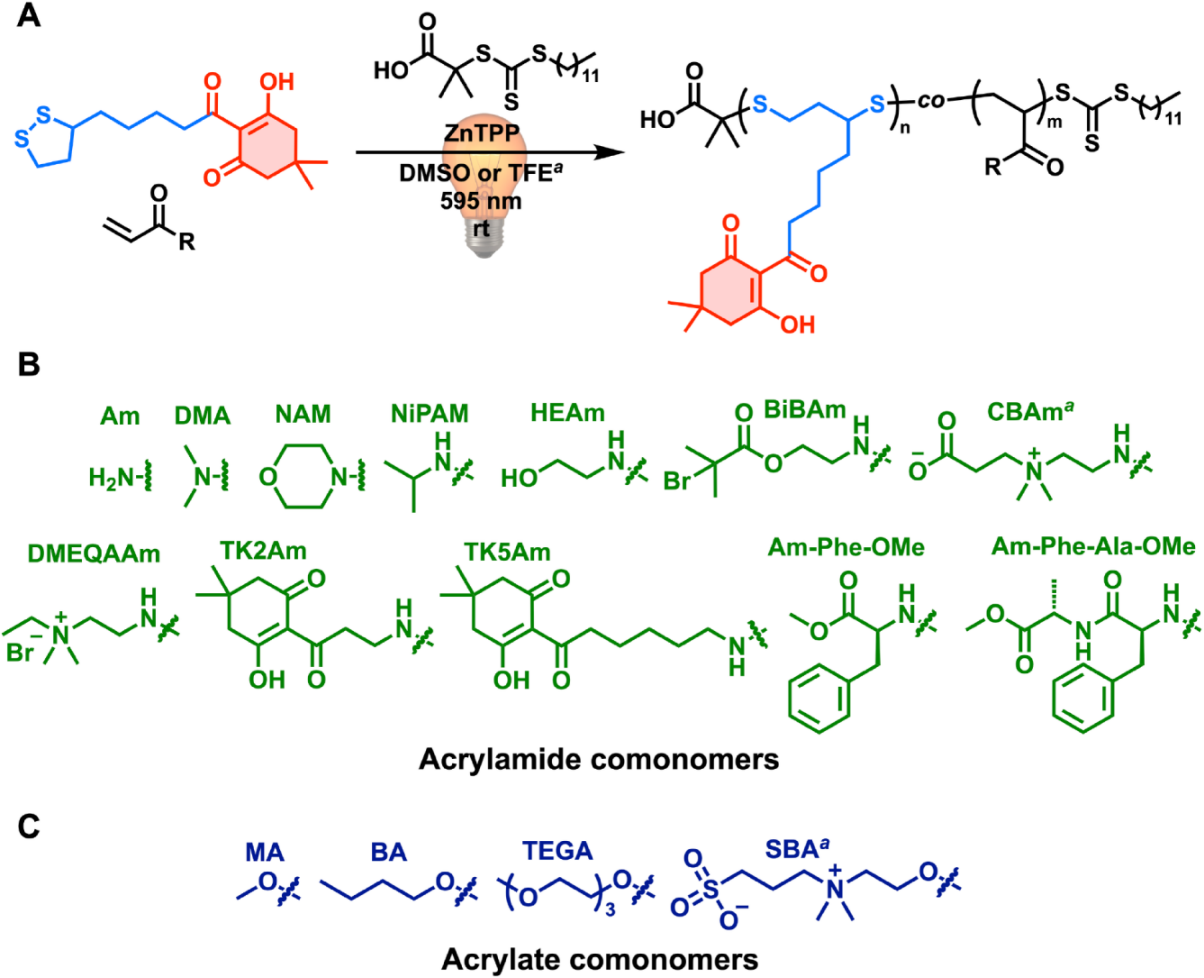
(A) Reaction scheme for the PET-RAFT copolymerization of TKLA with various vinyl monomers (B) Scope of acrylamide comonomers. (C) Scope of acrylate comonomers.

In addition, we attempted copolymerizations of TKLA with methyl acrylate (MA) at high *DP*_t_ values of 5,000, 10,000, and 20,000. As with DMA, these reactions were conducted at 10 mol % TKLA feed at −20 °C under 595 nm irradiation (3.0 mW cm^–2^) with prior deoxygenation (Table S3). Remarkably, we achieved molar masses approaching 600,000 g mol^–1^, comparable to our previous synthesis of LA-vinyl copolymers using photoRAFT polymerization,^39^ however with the additional feature of functionalization enabled by TK pendant groups.

All TKLA-vinyl copolymers were characterized by ^1^H NMR spectroscopy, which revealed the characteristic highly deshielded enolic TK proton (δ ∼ 18.2 ppm), confirming retention of the TK functionality and incorporation of degradable TKLA units along the backbone (Figures S16–S33). Collectively, these results highlight the compatibility of the PET RAFT ring-opening copolymerization of TKLA with a large range of vinyl monomers to yield highly functional and degradable copolymers that can be diversified postpolymerization via amine ligation. Notably, incorporation of zwitterionic and highly polar acrylamide comonomers afforded water-soluble polymers with backbone deconstruction encoded by disulfides, offering a useful entry point to highly functional degradable materials for biomedical applications.

### Postpolymerization Modification with Amines

After the successful synthesis of well-defined, degradable copolymers bearing TK side chains, we explored the scope of the PPM via DKE formation upon reaction with amines. Copolymers with TK moieties installed on each repeat unit, specifically P­(TKLA*-co*-TK2Am) and P­(TKLA*-co*-TK5Am), were reacted with aliphatic primary and secondary amines bearing various functional groups (Figure 4A). The condensation reactions were carried out in chloroform or DMF, without additional catalysts or coupling reagents, at room temperature. Formation of DKE functional groups was confirmed by ^1^H NMR spectroscopy (Figure 4B). After functionalization with benzylamine, the highly deshielded enolic TK resonances at 18.2 ppm (TKLA) and 17.7 ppm (TK2Am) disappeared (Figure 4B, top), accompanied by the appearance of the characteristic N–H resonance of the DKE moiety at 13.5 ppm (Figure 4B, bottom). We achieved quantitative PPM with a wide range of amines, including those with saturated, unsaturated, and aromatic moieties.

**Figure 4:**
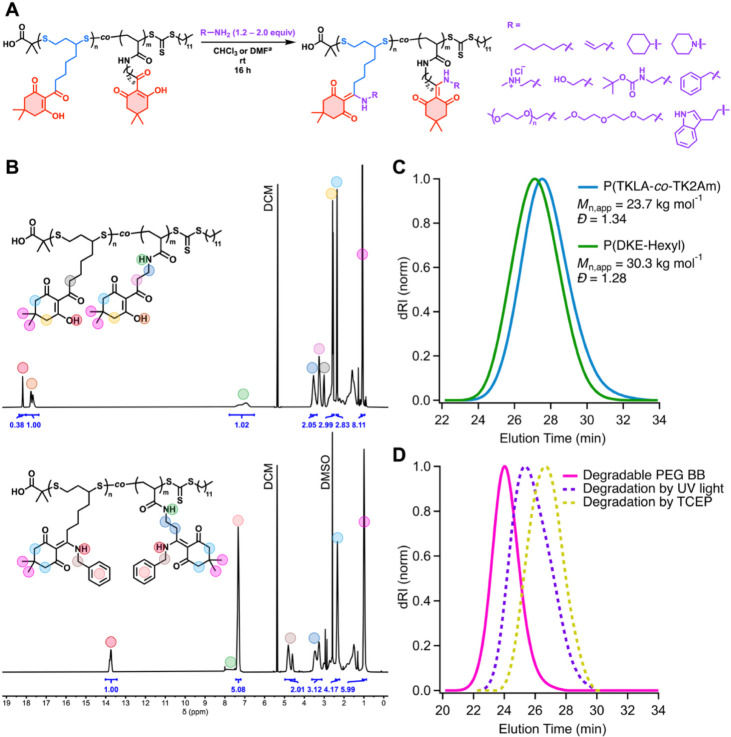
(A) Reaction scheme and amine scope of P­(TKLA*-co*-TK2Am) and P­(TKLA*-co*-TK5Am) functionalization. *^a^* Functionalization with *N,N*-dimethylethylenediamine, poly­(ethylene glycol) amine, and tryptamine was carried out in DMF. (B) ^1^H NMR spectra of P­(TKLA*-co*-TK2Am) (top) and P­(DKE-benzyl) (bottom), taken in DCM-*d*_2_. (C) Overlay of SEC traces of P­(TKLA-*co*-TK2Am) and of P­(DKE-hexyl), showing an increase in molar mass with no degradation postmodification. (D) SEC trace of polyethylene glycol (PEG) bottlebrush (BB) obtained by conjugation of poly­(ethylene glycol) amine (mPEG-NH_2_) to P­(TKLA*-co*-TK5Am), and SEC traces corresponding to the BB after degradation with tris-2-carboxyethyl phosphine hydrochloride (TCEP) and 370 nm UV light.

Importantly, amine functionalization proceeded without detectable polymer backbone degradation and resulted in a consistent shift in the SEC traces toward lower elution times, indicating an increase of molar mass and successful installation of side chain functionality (Figure 4C). Furthermore, functionalization with hydroxylamine showed the chemoselectivity of the reaction toward amines and its orthogonality to hydroxyl groups. Reaction with *N,N-*dimethylethylenediamine, followed by protonation of the tertiary amine side chains, allowed for facile installation of charged tertiary ammonium groups on the polymer (Figure S43), further highlighting the versatility of this PPM strategy.

An important feature of this PPM approach is its dynamicity and reversibility, with the ability of DKE linkages to undergo associative transamination with amines at elevated temperature.^26,30^ This contrasts with typical click-type postpolymerization modification protocols that employ static or “one-way” functionalization,^19,21^ limiting its use for the construction of polymers with recyclability, stimuli responsiveness, or self-healing properties.^6^ To prove the dynamic nature of the DKE side chains, we carried out an exchange reaction on P­(TKLA-*co*-TK5Am) with a 2-hydroxyethyl substituent (P­(DKE-hydroxyethyl). The transamination was conducted in the presence of 10 equiv. benzylamine at 50 °C in DMF solvent for 18 h to ensure equilibrium was established. We achieved 84% conversion to the *N*-benzyl DKE, evident by ^1^H NMR spectroscopy (Figure S50). The N–H proton in the starting material (δ = 13.4 ppm) diminished, with the concurrent emergence of the N–H proton at δ = 13.6 ppm corresponding to the *N*-benzyl DKE product. The observed conversion is consistent with an equilibrium-controlled transamination process, with a modest thermodynamic preference for the ethanolamine-derived diketoenamine.

Accordingly, this result highlights the ability to further reconfigure the DKE side chains of the polymer, even after the initial functionalization of the TK.

We also explored the potential to obtain degradable bottlebrush polymers (BBs) by conjugating methoxy poly­(ethylene glycol) amine (mPEG-NH_2_, *M*_n,app_ = 2.01 kg mol^–1^) to the side chain of P­(TKLA-*co*-TK5Am). The obtained BB possessed a significantly higher molar mass (*M*_n,app_ = 109,000 g mol^–1^, *Đ* = 1.16) compared to the precursor polymer (*M*_n,app_ = 25,000 g mol^–1^, *Đ* = 1.27), with a monomodal SEC trace (Figure 4D). This BB was then subsequently degraded using 100 mM tris-2-carboxyethyl phosphine hydrochloride (TCEP) in water/DMF mixture (1/3 v/v) and 370 nm UV irradiation, leading to a shift to higher elution times of SEC traces (Figure 4D). The UV degradability of the copolymers arises from photolytic cleavage of backbone disulfide bonds,^39,60^ along with possible radical generation from the trithiocarbonate chain ends.^61^ This highlights the dual-pathway functionality of the TKLA copolymers, with ability for both click-like functionalization and on-demand degradation.

Next, we tested the functionalization on HMW P­(TKLA-*co*-MA) and P­(TKLA-*co*-DMA). Using benzylamine and 2-[2-(2-methoxyethoxy)­ethoxy]­ethylamine (mPEG3-amine), we modified HMW P­(TKLA-*co*-MA). In both cases, complete conversion of the TK into DKE was observed (Figure 5D and Figure S52), and a clean shift to lower elution time of SEC traces before and after modification (Figure 5B).

**Figure 5:**
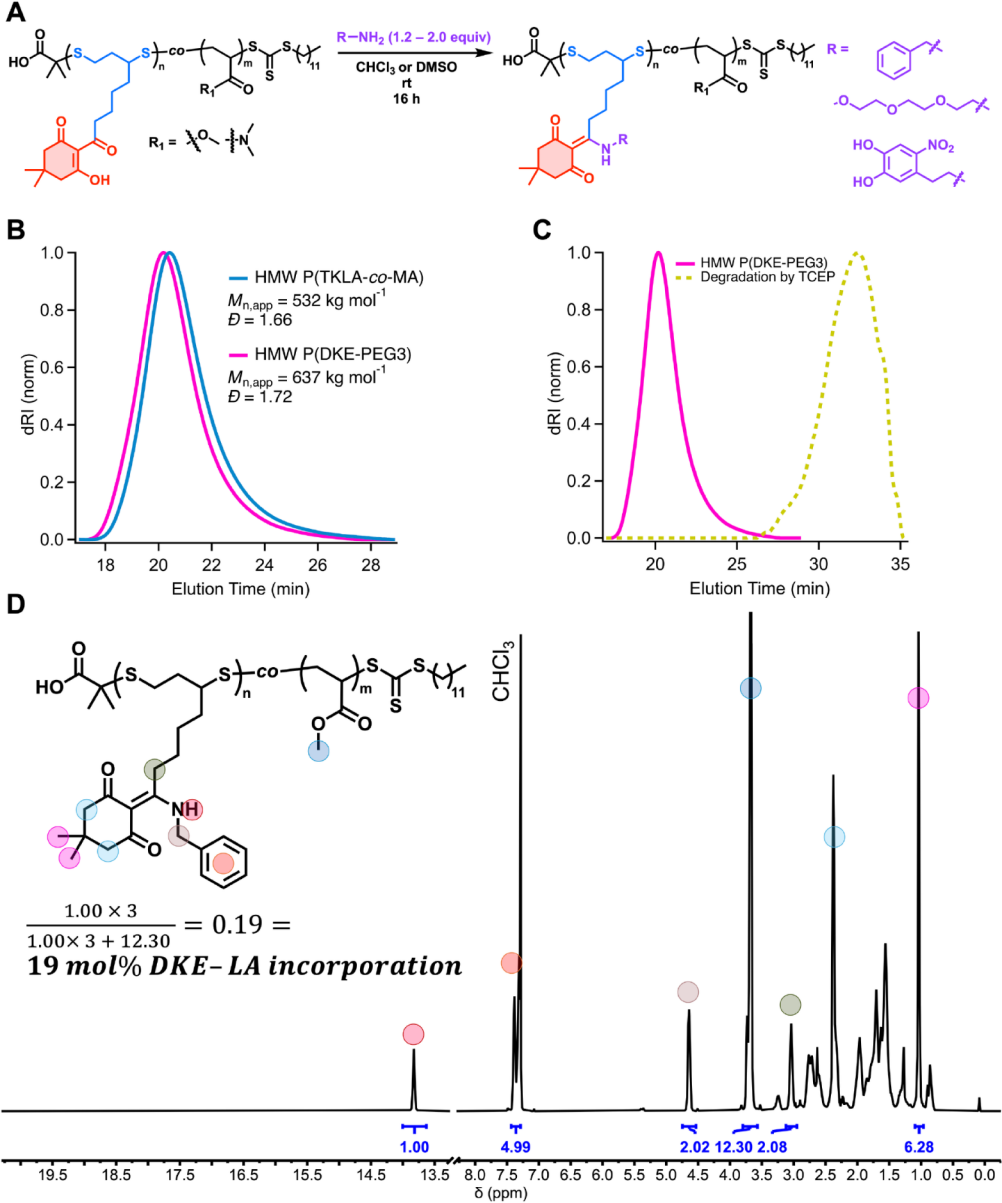
(A) Reaction scheme and amine scope of HMW P­(TKLA-*co*-MA) and P­(TKLA-*co*-DMA) functionalization. *^a^* Functionalization with 6-nitrodopmamine hemisulfate was carried out in DMSO with triethylamine added. (B) Overlay of SEC traces of HMW P­(TKLA-*co*-MA) and of HMW P­(DKE-mPEG3), showing increase in molar mass no degradation postmodification. (C) SEC traces corresponding to HMW P­(DKE-mPEG3) before and after degradation at 50 °C in water/DMF using TCEP. (D) ^1^H NMR spectrum of HMW P­(DKE-benzyl), showing complete conversion of TK into DKE, and higher incorporation of TKLA (19 mol %) compared to the feed used in copolymerization (10 mol %), owing to the low temperature conditions.

The HMW P­(DKE-mPEG_3_) was then subjected to degradation by 100 mM TCEP in water/DMF (1/3 v/v) at 50 °C for 48 h. Gratifyingly, the original molar mass of the polymer (*M*_n,app_ = 637,000 g mol^–1^) was reduced to below 1% of the original value (*M*_n,app_ = 3,150 g mol^–1^), with a clear shift to higher elution time evident by SEC (Figure 5C). This substantial decrease in molar mass is likely a result of some degradation of thioether linkages at elevated temperature via a retro-Michael addition, which proceeds more readily in polar solvents such as DMF and NMP.^37,62^ Moreover, the degradation is complemented by higher incorporation of S–S diads due to the low temperature conditions used in polymerization.

Additionally, HMW P­(TKLA-*co*-DMA) was functionalized with 6-nitrodopamine hemisulfate (ND) via DKE formation to afford a water-soluble, HMW polymer bearing nitrocatechol pendant groups. Nitrocatechols are well-known for their strong affinity toward metal oxide surfaces and their use in adhesive and surface-functional materials,^63,64^ while also serving as biomimetic motifs inspired by catechol-rich biological systems.^65,66^

The resulting conjugates combine high molecular weight, approaching that of natural macromolecules such as mucins,^67^ with efficient incorporation of nitrocatechol functionality along a degradable polymer backbone. Importantly, this PPM approach circumvents challenges associated with the direct polymerization of catechol-containing monomers^66^ and enables straightforward access to degradable, nitrocatechol-functional polymers.^68^

These features highlight the potential of the TKLA platform for applications in surface functionalization and biomimetic materials where high molecular weight, dense functionalization, and controlled degradability are desirable.

### Chain End Fidelity and Block Copolymer Synthesis

The synthesis of diblock copolymers is a hallmark of RDRP,^69–75^ which is otherwise not possible with conventional free radical polymerization methods. We envisioned applying our PET-RAFT ring-opening copolymerization approach to prepare polymers with a degradable block that was also easily modifiable postpolymerization. First, we prepared a low molar mass P­(TKLA-*co*-DMA) macroinitiator, by targeting a *DP* of 100 with 15 mol % feed of TKLA (*M*_n,app_ = 7,880 g mol^–1^, *Đ* = 1.15).

The macroinitiator was chain extended with two vinyl monomers: MA and *N*-acryloyl morpholine (NAM), yielding block copolymers P­(TKLA-*co*-DMA)-*b-*PMA (*M*_n,app_ = 44,500 g mol^–1^, *Đ* = 1.11) and P­(TKLA-*co*-DMA)-*b-*PNAM (*M*_n,app_ = 81,500 g mol^–1^, *Đ* = 1.14). SEC analysis revealed clean shifts from the macroinitiator to the block copolymers, with minimal tailing and lack of low-molecular-weight shoulders in the traces (Figure 6A). The successful chain extensions support excellent trithiocarbonate (TTC) ω-chain end fidelity, further evidenced by ^1^H NMR end group analysis of P­(TKLA-*co*-DMA), that shows integration of the methine proton α to the TTC moiety (δ = 5.23 ppm, 1H) agreeing well with the integration of methyl protons (δ = 0.87 ppm, 3H) of the dodecyl group (Figure 6C).

**Figure 6:**
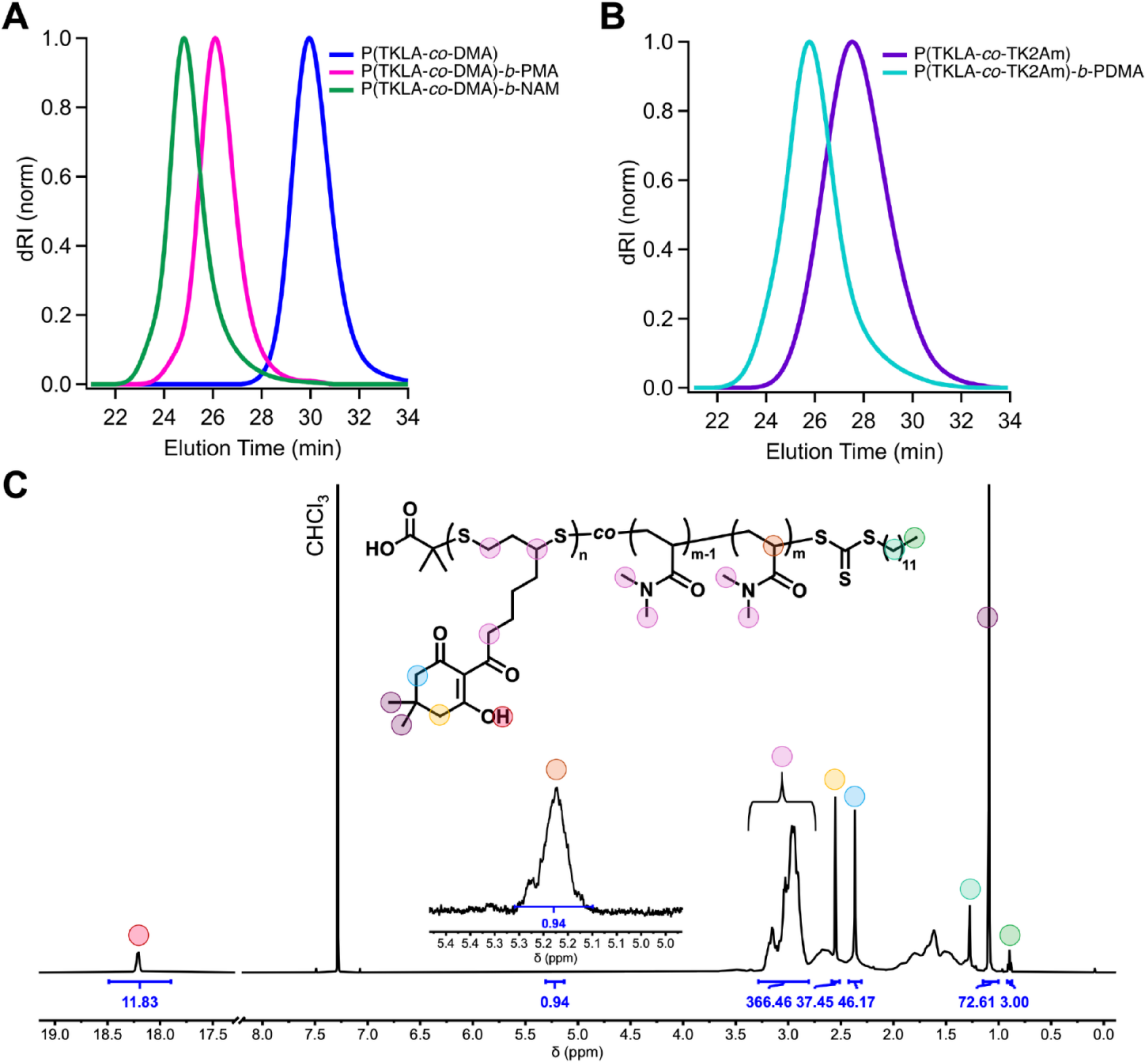
(A) SEC traces corresponding to chain extension of P­(TKLA-*co*-DMA)-TTC macroinitiator (*DP*_t_ = 100, *M*_n,app_ = 7,880 g mol^–1^) with MA and NAM, generating respective block copolymers P­(TKLA-*co*-DMA)-*b*-PMA (*M*_n,app_ = 44,500 g mol^–1^, *Đ* = 1.11) and P­(TKLA-*co*-DMA)-*b*-PNAM (*M*_n,app_ = 81,500 g mol^–1^, *Đ* = 1.14). (B) SEC traces corresponding to chain extension of P­(TKLA-*co*-TK2Am)-TTC macroinitiator (*M*_n,app_ = 24,400 g mol^–1^, *Đ* = 1.30) with DMA, yielding amphiphilic diblock P­(TKLA-*co*-TK2Am)-*b*-PDMA (*M*_n,app_ = 51, 800 g mol^–1^, *Đ* = 1.24) (C) ^1^H NMR end group analysis of P­(TKLA-*co*-DMA)-TTC, showing retention of trithiocarbonate (TTC) ω-chain end fidelity.

A diblock with a high TK-density degradable block was also prepared via chain extension of P­(TKLA-*co*-TK2Am) (*M*_n,app_ = 24,400 g mol^–1^, *Đ* = 1.30) with DMA, yielding amphiphilic P­(TKLA-*co*-TK2Am)-*b*-PDMA (*M*_n,app_ = 51,800 g mol^–1^, *Đ* = 1.24). Analysis by SEC confirmed a clean chain extension without unreacted macroinitiator (Figure 6B). The TK units on the hydrophobic block were then conjugated with *n*-hexylamine, yielding P­(DKE-hexyl)-*b*-PDMA with a hydrophobic core that could be self-assembled into well-defined micelles in water (Figure S59), for potential encapsulation of hydrophobic cargoes followed by on-demand release via degradation of the disulfides.

Disulfide-containing amphiphilic block copolymers have been recently presented for the construction of micellar nanoparticles (NPs) for the entrapment and on-demand delivery of hydrophobic drugs.^76,77^ We further explored the synthesis of micellar nanoparticles with functionalizable and degradable cores by chain extending commercially available poly­(ethylene glycol) methyl ether 2-(dodecylthiocarbonothioylthio)-2-methylpropionate (PEG-TTC, *M*_n,app_ = 6,000 g mol^–1^, *Đ* = 1.09) with TK2Am and 20 mol % feed of TKLA, at a TK block *DP*_t_ = 200 (Figure 7A). The resulting PEG-*b*-P­(TKLA-*co*-TK2Am) (*M*_n,app_ = 21,000, *Đ* = 1.29) had a relatively high 33 mol % incorporation of TKLA (Figure S60), and a clean shift of the SEC traces between macroinitiator and diblock copolymer (Figure 7B). This block copolymer was degraded in reducing conditions, using aqueous TCEP (100 mM) in water/DMF mixture (1/3 v/v) at rt. This led to a shift to higher elution times of SEC traces with the copolymer degrading to 25% of its original molar mass (*M*_n,app_ = 6,140 g mol^–1^, *Đ* = 1.55; Figure S63). PEG-*b*-P­(TKLA-*co*-TK2Am could also be conveniently self-assembled into micelles by dissolution in DMSO and dropwise addition into ultrapure water, forming micellar NPs with an intensity weighted mean hydrodynamic size (Z-average) of 46 nm measured by dynamic light scattering (DLS) (Figure 7C), and a relatively narrow polydispersity index (PDI = 0.176), typical of micellar self-assemblies used in drug delivery.^77–79^ Additionally, the modifiable TK block allows for tuning of NP size. We demonstrated this by conjugating *n*-hexylamine to PEG-*b*-P­(TKLA-*co*-TK2Am), resulting in PEG-*b*-P­(DKE-hexyl) (*M*_n,app_ = 24,900 g mol^–1^, *Đ* = 1.28). When NPs were formed by self-assembly of PEG-*b*-P­(DKE-hexyl), their size was larger than that of PEG-*b*-P­(TKLA-*co*-TK2Am) NPs (Z-average = 78 nm) (Figure 7D), while remaining narrowly dispersed (PDI = 0.196). Moreover, no signs of aggregation or precipitation were observed after several months of storage at rt indicating good colloidal and hydrolytic stability of the micelles (Table S4; Figure S62). These results underscore the effectiveness of this PPM strategy to yield amphiphilic diblock copolymers with conjugatable sites, enabling property tuning suited for construction of degradable drug or cargo carriers.

**Figure 7:**
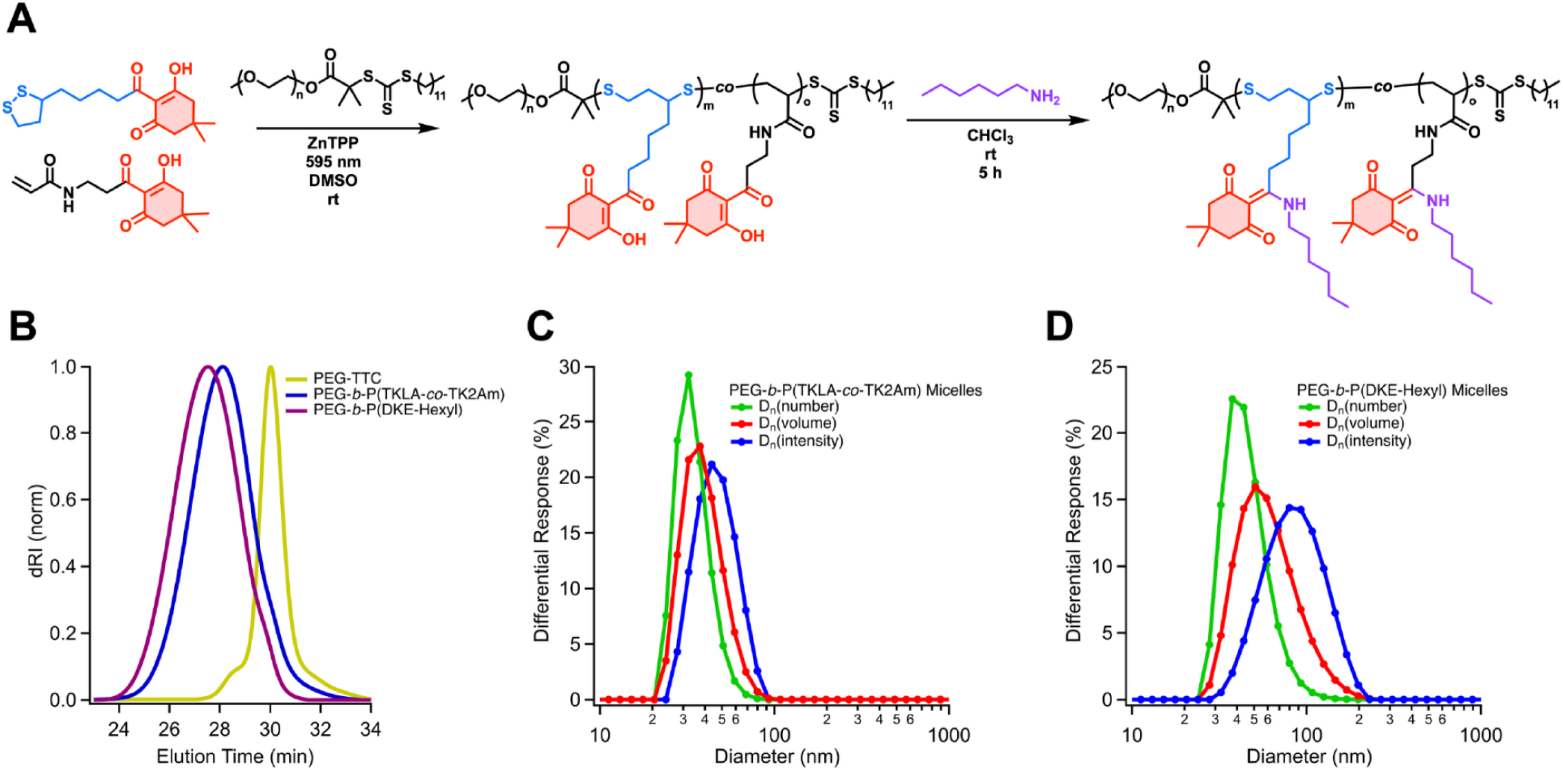
(A) Reaction scheme for the chain extension of PEG-TTC with TK2Am and TK5Am, followed by conjugation of *n*-hexylamine to the degradable TK block. (B) Overlay of SEC traces corresponding to PEG-TTC (*M*_n,app_ = 6,000 g mol^–1^, *Đ* = 1.09), PEG-*b*-P­(TKLA-*co*-TK2Am) (*M*_n,app_ = 21,000, *Đ* = 1.29), and PEG-*b*-P­(DKE-hexyl) (*M*_n,app_ = 24,900 g mol^–1^, *Đ* = 1.28). (C) Number-, volume-, and intensity-average hydrodynamic diameters of micelles as measured by dynamic light scattering DLS of self-assembled PEG-*b*-P­(TKLA-*co*-TK2Am) and (D) PEG-*b*-P­(DKE-hexyl).

## Conclusion

In summary, we introduce TKLA as a dual-functional 1,2-dithiolane monomer that unites click-type, catalyst-free amine ligation with programmable backbone deconstruction in a single polymer platform. Through ZnTPP-catalyzed PET-RAFT ring-opening copolymerization with a broad range of vinyl comonomers, TKLA enables controlled access to degradable copolymers bearing pendant β-triketone (TK) handles, including high-molecular-weight materials with molar masses approaching 600,000 g mol^–1^. Postpolymerization modification proceeds quantitatively through the catalyst-free condensation of TK with amines, enabling modular installation of hydrophilic, hydrophobic, charged, and bioactive substituents. Importantly, the resulting diketoenamine (DKE) motifs are not static, but enable postsynthetic remodeling via associative transamination under appropriate conditions, providing access to dynamically reconfigurable polymer side chains. Orthogonal to side-chain modification, the disulfides along the polymer backbone undergo selective and efficient cleavage under reducing environments, allowing controlled polymer deconstruction without compromising installed functionalities. Finally, we further highlight the versatility of this approach through the preparation of degradable amphiphilic block copolymers that can be postfunctionalized and self-assembled into micellar nanostructures, opening avenues for the design of responsive polymer architectures with tunable size, composition, and degradation behavior. Collectively, this dual-pathway molecular design strategy, combining dynamic click-type functionalization with programmed backbone degradability, provides a general framework for engineering polymer lifecycles and expands the scope of sustainable, functional, and adaptive macromolecular materials.

## Supplementary Material



## Notes

Materials, instrumentation, synthetic procedures, supplementary tables, and supplementary figures containing additional characterization, including polymerization kinetics, SEC data, and NMR spectra (PDF)
