# Supplementary material for: Dual-Pathway Strategy for Click-Type Functionalization and Programmable Polymer Deconstruction
Source: Macromolecules. 2026 Feb 18;59(5):2767–79. doi: 10.1021/acs.macromol.6c00380 (PMC12981018; doi:10.1021/acs.macromol.6c00380)
Supplement: Supplementary file 1 [file ma6c00380_si_001.pdf]

Supporting Information

Dual-Pathway Strategy for Click-Type Functionalization and Programmable Polymer Deconstruction

Ivan O. Levkovsky, Lucca Trachsel, Hironobu Murata, Krzysztof Matyjaszewski*

Department of Chemistry, Carnegie Mellon University, Pittsburgh, Pennsylvania 15213, USA

*E-mail: km3b@andrew.cmu.edu

Materials	S2
Instrumentation	S3
Synthesis	S4
Supplementary Tables	S15
Supplementary Figures	S17
References	S53

Materials

All reagents and solvents were purchased at the highest commercial grade and used as received unless otherwise noted. The following monomers were obtained from MilliporeSigma: acrylamide (Am, ≥99%), *N*,*N*-dimethylacrylamide (DMA, 99%, contains 500 ppm MEHQ), 4-acryloylmorpholine (NAM, 97%, contains 1,000 ppm MEHQ), *N*-isopropylacrylamide (NiPAM, 97%), *N*-hydroxyethyl acrylamide (HEAm, 97%, contains 1,000 ppm MEHQ), methyl acrylate (MA, 99%, contains 100 PPM MEHQ), *n*-butyl acrylate (BA, ≥99%, contains 10-60 ppm MEHQ). 3-((2-(Acryloyloxy)ethyl)dimethylammonio)propane-1-sulfonate (sulfobetaine acrylate, SBA, 98%) and 2-[2-(2-methoxyethoxy)ethoxy]ethyl acrylate (TEGA, >90.0%, stabilized with MEHQ) were purchased from Tokyo Chemical Industry. All liquid monomers were passed through a plug of basic alumina to remove inhibitors and acidic impurities and were stored at –20 °C. 1,4-Dioxane (ACS grade, Fisher) was passed through a column of basic alumina to remove adventitious peroxides prior to polymerization.

DL-*α*-Lipoic Acid (99%) was purchased from AmBeed. 6-acrylamidohexanoic acid (>98.0%) was purchased from TCI. Methoxy poly(ethylene glycol) amine (mPEG-NH2, avg. M_n = 2,010 g mol^{-1}) was purchased from BroadPharm. Poly(ethylene glycol) methyl ether 2-(dodecylthiocarbonothioylthio)-2-methylpropionate (PEG-TTC, avg. M_n = 6,000 g mol^{-1}) was purchased from MilliporeSigma. 2-(Butylthiocarbonothioylthio)propanoic acid (BTPA) was purchased from Boron Molecular and used as received.

2-(Dodecylthiocarbonothioylthio)-2-methylpropanoic acid (DDMAT),[1] *N*-ethyl-*N*,*N*-dimethylethyl quaternary ammonium acrylamide (DMEQAAm),[2] carboxybetaine acrylamide (CBA),[3] and 6-nitrodopamine hemisulfate were synthesized as reported previously.[4]

Deuterated chloroform ($CDCl_3$), deuterated dimethylsulfoxide (DMSO-d_6), deuterated dichloromethane (DCM-d_2), deuterated acetone (acetone-d_6), deuterated benzene (benzene-d_6), and deuterium oxide (D_2O) were obtained from Cambridge Isotope Laboratories.

Instrumentation

Polymerizations were conducted in a EvoluChem™ PhotoRedOx Box (HepatoChem) using green (525 nm, 14 mW cm^{-2}) or yellow (595 nm, 3.0–7.0 mW cm^{-2}) Kessil PR160L LEDs. Light intensity was measured at the location of the reaction vessels using a PM100D optical power meter (Thorlabs).

Degradation of PEG-BB was performed in EvoluChem™ PhotoRedOx Box (HepatoChem) using a UV PR160L-type LED (Kessil, 370 nm, 7.0 mW cm^{-2}). Light intensity was measured at the location of the reaction vessels using a PM100D optical power meter (Thorlabs).

^{1}H, ^{13}C, and 2D ^{1}H-^{13}C heteronuclear single-quantum correlation (HSQC) NMR spectra were collected using a Bruker Advance 500 MHz NMR spectrometer. $CDCl_3$, DMSO-d_6, DCM-d_2, acetone-d_6, benzene-d_6, and D_2O were used as solvents, and the residual solvent signal served as a reference. Monomer conversion was determined using ^{1}H NMR spectroscopy using ethylene carbonate as an internal standard.

Apparent number-average molar masses ($M_{n,app}$) and dispersities ($Đ$) were measured relative to poly(methyl methacrylate) (PMMA) standards using an Agilent 1260 Infinity II isocratic pump, a column set containing 3 PSS analytical columns (GRAM Lux 10^2, 10^3, 10^4, 10^5 Å pore sizes) and Agilent 1260 Infinity II refractive index (RI) detector with DMF + 50 mM LiBr as eluent at 50 °C with a flow rate of 1 mL min^{-1}.

Aqueous SEC-MALS characterizations for polymers not soluble in DMF including P(TKLA-*co*-CBAm), P(TKLA-*co*-DMEQAAm), and P(TKLA-*co*-SBA) were performed using Agilent SEC system (1260 Infinity II) equipped with UV detector, DAWN HELEOS-II (Wyatt) MALS detector and Optilab T-rEX (Wyatt Technology) RI detector. Measurements of P(TKLA-*co*-CBAm) and P(TKLA-*co*-SBA) were performed using a SUPREMA Lux 3000Å, 8 × 300 mm, 10 µm and guard column (PSS) at a flow rate of 0.5 mL min-1 with Dulbecco's Phosphate Buffered Saline and 0.02wt% sodium azide as eluent. Measurement of cationic P(TKLA-*co*-DMEQAAm) homopolymer was performed using a NOVEMA Max Lux 3000Å, 8 × 300 mm, 10 µm and guard column (PSS) at a flow rate of 0.5 mL min-1 with the mixture of 10 mM sodium phosphate (pH 4.5) and 100 mM sodium chloride as eluent.

Micelle sizes of self-assembled P(DKE-hexyl)-*b*-PDMA, PEG-*b*-P(TKLA-*co*-TK2Am), and PEG-*b*-P(DKE-hexyl) were determined using a Zetasizer Nano from Malvern Instruments, Ltd.

Synthesis

Synthesis of triketone-lipoic acid (TKLA)

In a 1 L round-bottom flask, α-lipoic acid (7.17 g, 34.8 mmol, 1.00 equiv) was dissolved in chloroform (200 mL), followed by the addition of dimedone (4.87 g, 34.8 mmol, 1.00 equiv) and 4-(dimethylamino)pyridine (DMAP, 6.37 g, 52.13 mmol, 1.50 equiv). Separately, 1-(3-dimethylaminopropyl)-3-ethylcarbodiimide hydrochloride (EDC-HCl, 8.00 g, 41.7 mmol, 1.2 equiv) was dissolved in 60 mL chloroform, and added dropwise under stirring to the reaction mixture, which was then stirred for 24 h at room temperature. Subsequently, an equal volume of 2 M HCl (260 mL) was added, and the reaction solution was strongly stirred for 1 h. After separation of the phases, the organic phase was washed with 2 M HCl (3 x 200 mL) and brine (1 x 200 mL), dried over anhydrous magnesium sulfate, filtered, and concentrated under vacuum. The crude, slightly opaque yellow oil was purified using silica column chromatography using ethyl acetate: hexanes (1:3 v/v) to yield a viscous clear yellow oil (8.64 g, 26.3 mmol, 75.7%). After characterization, a stock solution of the product was prepared in DMSO or 1,4-dioxane for use in polymerizations (500 mg mL^{-1}), and stored at –20 °C. ^{1}H NMR (500 MHz, $CDCl_3$): δ 18.15 (s, 1H), 3.56 (dq, *J* = 8.5, 6.4 Hz, 1H), 3.22 – 3.05 (m, 2H), 3.05 – 2.97 (m, 2H), 2.52 (s, 2H), 2.44 (dtd, *J* = 13.0, 6.6, 5.4 Hz, 1H), 2.33 (s, 2H), 1.90 (dq, *J* = 12.6, 6.9 Hz, 1H), 1.77 – 1.59 (m, 4H), 1.58 – 1.41 (m, 2H), 1.06 (s, 6H); ^{13}C NMR (126 MHz, benzene-d_6): δ 205.07, 197.11, 193.48, 111.88, 56.12, 52.11, 46.12, 40.05, 39.78, 38.12, 29.73, 28.85, 27.41, 24.41.

Synthesis of 3-acrylamidopropanoic acid

β-alanine (20.0 g, 225 mmol, 1.00 equiv) was dissolved in 100 mL water in a 500 mL round bottom flask, and the solution was cooled in an ice-water bath. To this, sodium hydroxide (18.0 g, 450 mmol, 2.00 equiv) was added. The reaction flask was flushed with argon, followed by dropwise addition of acryloyl chloride (22.0 mL, 225 mmol, 1.20 equiv). After stirring for 3 h, the pH of the reaction solution was lowered with HCl to <2, while stirring in an ice-water bath. The acidic solution was saturated with sodium chloride and then extracted with ethyl acetate (4 x 100 mL). The organic extracts were dried over anhydrous magnesium sulfate and concentrated under vacuum. The resulting crude product was recrystallized from ethyl acetate to yield a white crystalline solid (21.0 g, 147 mmol, 65.4 %). ^{1}H NMR (500 MHz, DMSO-d_6): δ 12.21 (s, 1H), 8.16 (t, *J* = 5.7 Hz, 1H), 6.21 (dd, *J* = 17.1, 10.1 Hz, 1H), 6.07 (dd, *J* = 17.1, 2.3 Hz, 1H), 5.56 (dd, *J* = 10.1, 2.3 Hz, 1H), 3.36 – 3.25 (m, 2H), 2.42 (t, *J* = 6.8 Hz, 2H); ^{13}C NMR (126 MHz, DMSO-d_6): δ 173.29, 165.09, 132.10, 125.47, 35.29, 34.19.

Synthesis of triketone ethylene acrylamide (TK2Am)

3-acrylamidopropanoic acid (9.75 g, 68.1 mmol, 1.00 equiv) and EDC-HCl (15.67 g, 81.7 mmol, 1.20 equiv) were added to a 500 mL round bottom flask, followed by 80 mL THF. To this suspension, a solution of dimedone (9.55 g, 68.1 mmol, 1.00 equiv) and DMAP (12.48 g, 103 mmol, 1.50 equiv) 80 mL DCM was added dropwise. The reaction was allowed to stir for 24 h. Subsequently, 2 M HCl (200 mL) was added, and the reaction solution was strongly stirred for 20 min. After separation of the phases, the organic phase was washed with 6 M HCl (1 x 200 mL), 2 M HCl (1 x 200 mL), and brine (1 x 200 mL), dried over anhydrous magnesium sulfate, filtered, and concentrated under vacuum. The crude yellow solid was recrystallized from acetonitrile to yield a white crystalline solid (11.7 g, 44.1 mmol, 64.8%). ^{1}H NMR (500 MHz, $CDCl_3$): δ 17.69 (s, 1H), 6.34 – 6.11 (m, 2H), 6.04 (dd, *J* = 17.0, 10.3 Hz, 1H), 5.60 (dd, *J* = 10.3, 1.5 Hz, 1H), 3.67 (q, *J* = 6.0 Hz, 2H), 3.29 (t, *J* = 6.0 Hz, 2H), 2.54 (s, 2H), 2.34 (s, 2H), 1.07 (s, 6H) ^{13}C NMR (126 MHz, $CDCl_3$): δ 204.19, 196.60, 195.22, 165.30, 130.93, 126.22, 112.38, 112.36, 52.36, 46.22, 40.64, 34.83, 30.76, 28.13.

Synthesis of triketone-pentamethylene acrylamide (TK5Am)

In a 500 mL round bottom flask, 6-acrylamidopropanoic acid (5.89 g, 31.8 mmol, 1.00 equiv), dimedone (4.46 g, 31.8 mmol, 1.00 equiv), and DMAP (5.83 g, 1.5 equiv) were dissolved in 140 mL chloroform. Separately, EDC-HCl (7.32 g, 38.2 mmol, 1.20 equiv) was dissolved in 60 mL chloroform, and added dropwise to the reaction mixture, which was then stirred for 24 h at room temperature. Subsequently, an equal volume of 2 M HCl (140 mL) was added, and the reaction solution was strongly stirred for 1 h. After separation of the phases, the organic phase was washed with 2 M HCl (3 x 200 mL) and brine (1 x 200 mL), dried over anhydrous magnesium sulfate, filtered, and concentrated under vacuum yielding a yellow oil. The crude product was purified using silica column chromatography using ethyl acetate, yielding a light-yellow oil which crystallized upon cooling to –20 °C. The off-white crystals were dried under high vacuum to remove residual solvent (5.46 g, 17.8 mmol, 55.9%). ^1H NMR (500 MHz, $CDCl_3$): δ 18.15 (s, 1H), 6.24 (dd, J = 17.0, 1.6 Hz, 1H), 6.11 (dd, J = 17.0, 10.2 Hz, 2H), 5.59 (dd, J = 10.2, 1.6 Hz, 1H), 3.32 (td, J = 7.1, 5.8 Hz, 2H), 3.06 – 2.93 (m, 2H), 2.52 (s, 2H), 2.33 (s, 2H), 1.69 – 1.49 (m, 4H), 1.47 – 1.34 (m, 2H), 1.06 (s, 6H); ^{13}C NMR (126 MHz, $CDCl_3$): δ 205.25, 197.73, 195.14, 165.57, 131.05, 125.96, 111.90, 52.60, 46.76, 40.10, 39.30, 30.61, 29.14, 28.12, 26.58, 24.17.

General procedure for PET-RAFT copolymerization of TKLA with DMA

DMA (444 µL, 4.31 mmol, 170 equiv), TKLA (500 µL of 500 mg mL^{-1} stock solution in DMSO, 0.760 mmol, 30.0 equiv), DDMAT (88.0 µL of 105 mg mL^{-1} stock solution in DMSO, 25.4 µmol, 1.00 equiv), zinc tetraphenylporphyrin (ZnTPP, 127 µL of 1.35 mg mL^{-1} stock solution in DMSO, 0.254 µmol, 0.010 equiv), and ethylene carbonate (50 mg as an internal standard to determine monomer conversion) were added to a 4 mL shell vial, and diluted with DMSO (531 µL, [M]tot = 3.00 M). Prior to irradiation, the vial was vortexed for 20 s to homogenize the components, and a 15 µL aliquot was mixed with 600 µL acetone-d_6 to determine the initial monomer conversion (t = 0) by ^1H NMR spectroscopy. The polymerization mixture was then irradiated with yellow LEDs (595 nm, 7.0 mW cm^{-2}) for 3 h, and aliquots were taken at defined timepoints for analysis by ^1H NMR spectroscopy for monomer conversion and SEC with DMF + 50 mM LiBr as eluent for molar mass and dispersity ($Đ$). To purify the polymer, the viscous solution was diluted with chloroform and precipitated into ice-cold diethyl ether three times and finally dried under high vacuum resulting in a yellow-green powder ($M_{n,theory}$ = 24,200 g mol^{-1}, $M_{n,app}$ = 24,700 g mol^{-1}, $Đ$ = 1.14).

General procedure for PET-RAFT copolymerization of TKLA with TK2Am

TK2Am (1.02 g, 3.85 mmol, 160 equiv), TKLA (632 µL of 500 mg mL^{-1} stock solution in DMSO, 0.962 mmol, 40.0 equiv), DDMAT (87.0 µL of 105 mg mL^{-1} stock solution in DMSO, 24.1 µmol, 1.00 equiv), zinc tetraphenylporphyrin (ZnTPP, 121 µL of 1.35 mg mL^{-1} stock solution in DMSO, 0.241 µmol, 0.010 equiv), and ethylene carbonate (50 mg as an internal standard to determine monomer conversion) were added to a 4 mL shell vial, and diluted with DMSO (764 µL, [M]tot = 3.00 M). Prior to irradiation, the vial was vortexed for 20 s to homogenize the components, and a 15 µL aliquot was mixed with 600 µL acetone-d_6 to determine the initial monomer conversion (t = 0) by ^1H NMR spectroscopy. The polymerization mixture was then irradiated with yellow LEDs (595 nm, 7.0 mW cm^{-2}) for 2 h, and aliquots were taken at defined timepoints for analysis by ^1H NMR spectroscopy for monomer conversion and SEC with DMF + 50 mM LiBr as eluent for molar mass and dispersity ($Đ$). To purify the polymer, the viscous solution was diluted with chloroform and precipitated into ice-cold diethyl ether three times and finally dried under high vacuum resulting in a yellow powder ($M_{n,theory}$ = 31,500 g mol^{-1}, $M_{n,app}$ = 24,000 g mol^{-1}, $Đ$ = 1.32).

General procedure for PET-RAFT copolymerization of TKLA with TK5Am

TK5Am (749 mg, 2.44 mmol, 160 equiv), TKLA (400 µL of 500 mg mL^{-1} stock solution in DMSO, 0.609 mmol, 40.0 equiv), DDMAT (54.9 µL of 101 mg mL^{-1} stock solution in DMSO, 15.2 µmol, 1.00 equiv), zinc tetraphenylporphyrin (ZnTPP, 121 µL of 1.12 mg mL^{-1} stock solution in DMSO, 0.152 µmol, 0.010 equiv), and ethylene carbonate (50 mg as an internal standard to determine monomer conversion) were added to a 4 mL shell vial, and diluted with DMSO (764 µL, [M]tot = 3.00 M). The vial was vortexed for 20 s to homogenize the components, and a 15 µL aliquot was mixed with 600 µL acetone-d_6 to determine the initial monomer conversion (t = 0) by ^{1}H NMR spectroscopy. The vial was then fitted with a rubber septum, and the reaction was sparged with argon for 15 min prior to being irradiated with yellow LEDs (595 nm, 7.0 mW cm^{-2}) for 4 h. Aliquots were taken using a syringe at defined timepoints for analysis by ^{1}H NMR spectroscopy for monomer conversion and SEC with DMF + 50 mM LiBr as eluent for molar mass and dispersity (*Đ*). To purify the polymer, the viscous solution was diluted with chloroform and precipitated into ice-cold diethyl ether three times and finally dried under high vacuum resulting in a yellow powder ($M_{n,theory}$ = 33,200 g mol^{-1}, $M_{n,app}$ = 25,000 g mol^{-1}, *Đ* = 1.27).

Kinetic study of PET-RAFT copolymerization of TKLA with DMA

The kinetic analysis followed the general procedure for PET-RAFT copolymerization of TKLA with DMA. The reaction mixture was irradiated under yellow light (595 nm, 7.0 mW cm^{-2}) for 120 min. At defined time intervals (0, 10, 15, 20, 30, 45, 60, 90, 120 min) 10 µL aliquots were taken for analysis. Monomer conversion was determined by ^{1}H NMR spectroscopy, and molar mass and dispersity (*Đ*) were measured by SEC using DMF + 50 mM LiBr as eluent.

Kinetic study of PET-RAFT copolymerization of TKLA with TK2Am

The kinetic analysis followed the general procedure for PET-RAFT copolymerization of TKLA with MA. The reaction mixture was irradiated under yellow light (595 nm, 7.0 mW cm^{-2}) for 60 min. At defined time intervals (0, 10, 15, 30, 45, 60 min) 10 µL aliquots were taken for analysis. Monomer conversion was determined by ^{1}H NMR spectroscopy, and molar mass and dispersity (*Đ*) were measured by SEC using DMF + 50 mM LiBr as eluent.

Copolymerization of TKLA with DMA targeting varying degrees of polymerization

For DP_T = 100–2,000, target degree of polymerization (DP_T) values were varied by adjusting the DDMAT to total monomer ratio, with DMA : TKLA molar ratio = 8.5:1.5, and ZnTPP at 0.01 equiv with respect to DDMAT). For higher DP_T = 1,000 and 2,000, DDMAT was added as a more dilute stock solution (20.2 mg mg mL^{-1} in DMSO), and DMSO was added accordingly to ensure [M]tot = 3.00 M. Ethylene carbonate (50 mg) was added as an internal standard to determine monomer conversion. Polymerizations were irradiated under yellow light (595 nm, 7.0 mW cm^{-2}) in a capped vial without prior deoxygenation, until monomer conversion was observed to significantly slow or stop by ^{1}H NMR spectroscopy. The copolymers were analyzed by SEC using DMF + 50 mM LiBr as eluent.

For DP_T = 5,000 and 10,000 to obtain high-molecular weight (HMW) P(TKLA-*co*-DMA), DMA : TKLA molar ratio was set to 9 : 1. DDMAT was added as a more dilute stock solution (1.12 mg mL^{-1} in DMSO), along with ZnTPP (0.01 equiv with respect to DDMAT, 0.240 mg mL^{-1} in DMSO). DMSO was added accordingly to ensure [M]tot = 4.00 M. Ethylene carbonate (50 mg) was added as an internal standard to determine monomer conversion. The reaction mixtures were then deoxygenated by sealing the vial with a septum, and sparging with argon for 10 min. The polymerizations were carried out in a freezer at –20 °C, irradiating with yellow light (595 nm, 3.0 mW cm^{-2}) until DMA monomer concentration was depleted to the point where

the reaction mixture froze (8 h). The copolymers were analyzed by by SEC using DMF + 50 mM LiBr as eluent. HMW P(TKLA-*co*-DMA) was purified by first diluting the polymerization mixtures with chloroform, precipitation into cold diethyl ether 3 times, and drying under high vacuum.

Copolymerization of TKLA with TK2Am targeting varying degrees of polymerization

Target degree of polymerization (DP_T = 100, 200, 500) values were varied by adjusting the DDMAT to total monomer ratio, with DMA : TKLA molar ratio = 8.5:1.5, and ZnTPP at 0.01 equiv with respect to DDMAT). DMSO was added accordingly to ensure [M]tot = 3.00 M. Ethylene carbonate (50 mg) was added as an internal standard to determine monomer conversion. Polymerizations were irradiated under yellow light (595 nm, 7.0 mW cm^{-2}) in a capped vial without prior deoxygenation, until monomer conversion was observed to significantly slow or stop by ^1H NMR spectroscopy. The copolymers were analyzed by SEC using DMF + 50 mM LiBr as eluent.

Synthesis of high molecular weight (HMW) P(TKLA-*co*-MA)

In a 2 mL HPLC vial, MA (186 µL, 2.05 mmol, 9.00 × 10^3 equiv), TKLA (150 µL of 500 mg mL^{-1} stock solution in DMSO, 0.230 mmol, 1.00 × 10^3 equiv), DDMAT (74.0 µL of 1.12 mg mL^{-1} stock solution in DMSO, 0.228 µmol, 1.00 equiv), ZnTPP (6.50 µL of 0.240 mg mL^{-1} stock solution in DMSO, 2.28 × 10^{-3} µmol, 0.01 equiv), and ethylene carbonate (50 mg as an internal standard to determine monomer conversion) were combined. DMSO (154 µL, [M]tot = 4.00 M) was added, and the reaction mixtures were deoxygenated by sealing the vial with a septum and sparging with argon for 10 min. The polymerization was carried out in a freezer at –20 °C, irradiating with yellow light (595 nm, 3.0 mW cm^{-2}) until MA monomer concentration was depleted to the point where the reaction mixture froze (4 h). The copolymers were analyzed by by SEC using DMF + 50 mM LiBr as eluent. HMW P(TKLA-*co*-MA) was purified by first diluting the polymerization mixtures with chloroform, precipitation into cold diethyl ether : hexanes mixture (1:1 v/v) 3 times, and drying under high vacuum to yield a white solid ($M_{n,theory}$ = 633,000 g mol^{-1}, $M_{n,app}$ = 426,000 g mol^{-1}, $Đ$ = 1.44).

Target degree of polymerization (DP_T = 2,000; 5,000; 10,000; 20,000) values were varied by adjusting the DDMAT to total monomer ratio, with DMA : TKLA molar ratio = 9:1, and ZnTPP at 0.01 equiv with respect to DDMAT).

Scope of PET-RAFT copolymerization of TKLA with various acrylate and acrylamide monomers

<u>P(TKLA-*co*-Am)</u>

Acrylamide (Am, 123 mg, 1.73 mmol, 170 equiv), TKLA (250 µL of 400 mg mL^{-1} stock solution in DMSO, 0.304 mmol, 30.0 equiv), BTPA (116 µL of 20.8 mg mL^{-1} stock solution in DMSO, 10.2 µmol, 1.00 equiv), zinc tetraphenylporphyrin (ZnTPP, 51.0 µL of 1.35 mg mL^{-1} stock solution in DMSO, 0.102 µmol, 0.010 equiv), and ethylene carbonate (50 mg as an internal standard to determine monomer conversion) were added to a 2 mL HPLC vial, and diluted with DMSO (340 µL, [M]tot = 4.00 M). Prior to irradiation, the vial was vortexed for 20 s to homogenize the components, and a 10 µL aliquot was mixed with 600 µL DMSO-d_6 to determine the initial monomer conversion (t = 0) by ^1H NMR spectroscopy. The polymerization mixture was then irradiated with yellow LEDs (595 nm, 7.0 mW cm^{-2}) for 3 h, and aliquots were taken at defined timepoints for analysis by ^1H NMR spectroscopy for monomer conversion and SEC with DMF + 50 mM LiBr as eluent for molar mass and dispersity ($Đ$). To purify the polymer, the reaction mixture was diluted with potassium hydroxide solution (100 mM) and dialyzed against deionized water using a regenerated cellulose membrane (3.5 kDa molecular weight cutoff). After lyophilization, the polymer was obtained as a white solid. ($M_{n,theory}$ = 20,000 g mol^{-1}, $M_{n,app}$ = 8,700 g mol^{-1}, $Đ$ = 1.25).

P(TKLA-*co*-NAM)

N-acryloyl morpholine (NAM, 174 µL, 1.37 mmol, 170 equiv), TKLA (200 µL of 400 mg mL^{-1} stock solution in DMSO, 0.244 mmol, 30.0 equiv), DDMAT (147 µL of 20.2 mg mL^{-1} stock solution in DMSO, 8.07 µmol, 1.00 equiv), zinc tetraphenylporphyrin (ZnTPP, 40.8 µL of 1.35 mg mL^{-1} stock solution in DMSO, 0.081 µmol, 0.010 equiv), and ethylene carbonate (50 mg as an internal standard to determine monomer conversion) were added to a 2 mL HPLC vial, and diluted with DMSO (180 µL, [M]tot = 3.00 M). Prior to irradiation, the vial was vortexed for 20 s to homogenize the components, and a 10 µL aliquot was mixed with 600 µL acetone-d_6 to determine the initial monomer conversion (t = 0) by ^{1}H NMR spectroscopy. The polymerization mixture was then irradiated with yellow LEDs (595 nm, 7.0 mW cm^{-2}) for 2 h, and aliquots were taken at defined timepoints for analysis by ^{1}H NMR spectroscopy for monomer conversion and SEC with DMF + 50 mM LiBr as eluent for molar mass and dispersity (*Đ*). To purify the polymer, the reaction mixture was diluted with chloroform and precipitated into ice-cold diethyl ether three times and finally dried under high vacuum resulting in a light-green powder ($M_{n,theory}$ = 33,300 g mol^{-1}, $M_{n,app}$ = 25,300 g mol^{-1}, *Đ* = 1.20).

P(TKLA-*co*-NiPAM)

N-isopropyl acrylamide (NiPAM, 156 mg, 1.37 mmol, 170 equiv), TKLA (200 µL of 400 mg mL^{-1} stock solution in DMSO, 0.244 mmol, 30.0 equiv), DDMAT (147 µL of 20.2 mg mL^{-1} stock solution in DMSO, 8.07 µmol, 1.00 equiv), zinc tetraphenylporphyrin (ZnTPP, 40.8 µL of 1.35 mg mL^{-1} stock solution in DMSO, 0.081 µmol, 0.010 equiv), and ethylene carbonate (50 mg as an internal standard to determine monomer conversion) were added to a 2 mL HPLC vial, and diluted with DMSO (354 µL, [M]tot = 3.00 M). Prior to irradiation, the vial was vortexed for 20 s to homogenize the components, and a 10 µL aliquot was mixed with 600 µL acetone-d_6 to determine the initial monomer conversion (t = 0) by ^{1}H NMR spectroscopy. The polymerization mixture was then irradiated with yellow LEDs (595 nm, 7.0 mW cm^{-2}) for 2 h, and aliquots were taken at defined timepoints for analysis by ^{1}H NMR spectroscopy for monomer conversion and SEC with DMF + 50 mM LiBr as eluent for molar mass and dispersity (*Đ*). To purify the polymer, the reaction mixture was diluted with chloroform and precipitated into ice-cold diethyl ether three times and finally dried under high vacuum resulting in a light-green powder ($M_{n,theory}$ = 33,300 g mol^{-1}, $M_{n,app}$ = 25,300 g mol^{-1}, *Đ* = 1.20).

P(TKLA-*co*-HEAm)

Hydroxyethyl acrylamide (HEAm, 143 µL , 1.37 mmol, 170 equiv), TKLA (200 µL of 400 mg mL^{-1} stock solution in DMSO, 0.244 mmol, 30.0 equiv), DDMAT (147 µL of 20.2 mg mL^{-1} stock solution in DMSO, 8.07 µmol, 1.00 equiv), zinc tetraphenylporphyrin (ZnTPP, 40.8 µL of 1.35 mg mL^{-1} stock solution in DMSO, 0.081 µmol, 0.010 equiv), and ethylene carbonate (50 mg as an internal standard to determine monomer conversion) were added to a 2 mL HPLC vial, and diluted with DMSO (211 µL, [M]tot = 3.00 M). Prior to irradiation, the vial was vortexed for 20 s to homogenize the components, and a 10 µL aliquot was mixed with 600 µL DMSO-d_6 to determine the initial monomer conversion (t = 0) by ^{1}H NMR spectroscopy. The polymerization mixture was then irradiated with yellow LEDs (595 nm, 7.0 mW cm^{-2}) for 2 h, and aliquots were taken at defined timepoints for analysis by ^{1}H NMR spectroscopy for monomer conversion and SEC with DMF + 50 mM LiBr as eluent for molar mass and dispersity (*Đ*). To purify the polymer, the reaction mixture was diluted with methanol and precipitated into ice-cold diethyl ether three times and finally dried under high vacuum resulting in a light-green powder ($M_{n,theory}$ = 24,900 g mol^{-1}, $M_{n,app}$ = 24,900 g mol^{-1}, *Đ* = 1.19).

P(TKLA-*co*-BiBAm)

α-Bromoisobutyryl acrylamide (BiBAm, 280 µL , 1.37 mmol, 170 equiv), TKLA (200 µL of 400 mg mL^{-1} stock solution in DMSO, 0.244 mmol, 30.0 equiv), DDMAT (28.3 µL of 105 mg mL^{-1} stock solution in DMSO, 8.07 µmol, 1.00 equiv), zinc tetraphenylporphyrin (ZnTPP, 40.8 µL of 1.35 mg mL^{-1} stock solution in DMSO, 0.081 µmol, 0.010 equiv), and ethylene carbonate (50 mg as an internal standard to determine monomer conversion) were added to a 2 mL HPLC vial. The final [M]tot was 2.95 M, and no additional DMSO was added. The vial was vortexed for 20 s to homogenize the components, and a 15 µL aliquot was mixed with 600 µL acetone-d_6 to determine the initial monomer conversion (t = 0) by ^{1}H NMR spectroscopy. The vial was then fitted with a rubber septum, and the reaction was sparged with argon for 10 min prior to being irradiated with yellow LEDs (595 nm, 7.0 mW cm^{-2}) for 2 h. Aliquots were taken at defined timepoints for analysis by ^{1}H NMR spectroscopy for monomer conversion and SEC with DMF + 50 mM LiBr as eluent for molar mass and dispersity (*Đ*). To purify the polymer, the reaction mixture was diluted with chloroform and precipitated into ice-cold diethyl ether three times and finally dried under high vacuum resulting in a yellow powder ($M_{n,theory}$ = 40,700 g mol^{-1}, $M_{n,app}$ = 22,800 g mol^{-1}, *Đ* = 1.30).

P(TKLA-*co*-CBAm)

Carboxybetaine acrylamide (CBAm, 118 mg, 0.550 mmol, 170 equiv), TKLA (80 µL of 400 mg mL^{-1} stock solution in DMSO, 0.100 mmol, 30.0 equiv), DDMAT (11.3 µL of 105 mg mL^{-1} stock solution in DMSO, 3.25 µmol, 1.00 equiv), zinc tetraphenylporphyrin (ZnTPP, 16.8 µL of 1.35 mg mL^{-1} stock solution in DMSO, 0.033 µmol, 0.010 equiv), and ethylene carbonate (50 mg as an internal standard to determine monomer conversion) were added to a 2 mL HPLC vial, and diluted wit 2,2,2-trifluoroethanol (TFE, 217 µL, [M]tot = 2.00 M). The vial was vortexed for 20 s to homogenize the components, and a 10 µL aliquot was mixed with 600 µL D_2O to determine the initial monomer conversion (t = 0) by ^{1}H NMR spectroscopy. The vial was then fitted with a rubber septum, and the reaction was sparged with argon for 10 min prior to being irradiated with yellow LEDs (595 nm, 7.0 mW cm^{-2}) for 1h. Aliquots were taken at defined timepoints for analysis by ^{1}H NMR spectroscopy for monomer conversion and SEC with DMF + 50 mM LiBr as eluent for molar mass and dispersity (*Đ*). To purify the polymer, the reaction mixture was diluted with deionized water and dialyzed against deionized water using a regenerated cellulose membrane (3.5 kDa molecular weight cutoff). After lyophilization, the polymer was obtained as a white solid ($M_{n,theory}$ = 40,700 g mol^{-1}, $M_{n,abs}$ = 53,700 g mol^{-1}, *Đ* = 1.28).

P(TKLA-*co*-Am-Phe-OMe)

Acrylamide-phenylalanine methyl ester (Am-Phe-OMe, 322 mg , 1.37 mmol, 170 equiv), TKLA (200 µL of 400 mg mL^{-1} stock solution in DMSO, 0.244 mmol, 30.0 equiv), DDMAT (147 µL of 20.2 mg mL^{-1} stock solution in DMSO, 8.07 µmol, 1.00 equiv), zinc tetraphenylporphyrin (ZnTPP, 40.8 µL of 1.35 mg mL^{-1} stock solution in DMSO, 0.081 µmol, 0.010 equiv), and ethylene carbonate (50 mg as an internal standard to determine monomer conversion) were added to a 2 mL HPLC vial, and diluted with DMSO (354 µL, [M]tot = 1.50 M). Prior to irradiation, the vial was vortexed for 20 s to homogenize the components, and a 10 µL aliquot was mixed with 600 µL DMSO-d_6 to determine the initial monomer conversion (t = 0) by ^{1}H NMR spectroscopy. The polymerization mixture was then irradiated with yellow LEDs (595 nm, 7.0 mW cm^{-2}) for 8 h, and aliquots were taken at defined timepoints for analysis by ^{1}H NMR spectroscopy for monomer conversion and SEC with DMF + 50 mM LiBr as eluent for molar mass and dispersity (*Đ*). To purify the polymer, the reaction mixture was diluted with acetone and precipitated into ice-cold diethyl ether three times and finally dried under high vacuum resulting in a yellow powder ($M_{n,theory}$ = 46,500 g mol^{-1}, $M_{n,app}$ = 37,900 g mol^{-1}, *Đ* = 1.70).

P(TKLA-*co*-Am-Phe-Ala-OMe)

Acrylamide-phenylalanine-alanine methyl ester (Am-Phe-Ala-OMe, 420 mg, 1.37 mmol, 170 equiv), TKLA (200 µL of 400 mg mL^{-1} stock solution in DMSO, 0.244 mmol, 30.0 equiv), DDMAT (147 µL of 20.2 mg mL^{-1} stock solution in DMSO, 8.07 µmol, 1.00 equiv), zinc tetraphenylporphyrin (ZnTPP, 40.8 µL of 1.35 mg mL^{-1} stock solution in DMSO, 0.081 µmol, 0.010 equiv), and ethylene carbonate (50 mg as an internal standard to determine monomer conversion) were added to a 2 mL HPLC vial, and diluted with DMSO (354 µL, [M]tot = 1.40 M). Prior to irradiation, the vial was vortexed for 20 s to homogenize the components, and a 10 µL aliquot was mixed with 600 µL DMSO-d_6 to determine the initial monomer conversion (t = 0) by ^{1}H NMR spectroscopy. The polymerization mixture was then irradiated with yellow LEDs (595 nm, 7.0 mW cm^{-2}) for 8 h, and aliquots were taken at defined timepoints for analysis by ^{1}H NMR spectroscopy for monomer conversion and SEC with DMF + 50 mM LiBr as eluent for molar mass and dispersity (*Đ*). To purify the polymer, the reaction mixture was diluted with acetone and precipitated into ice-cold diethyl ether three times and finally dried under high vacuum resulting in a yellow powder ($M_{n,theory}$ = 54,400 g mol^{-1}, $M_{n,app}$ = 43,100 g mol^{-1}, *Đ* = 1.65).

P(TKLA-*co*-MA)

Methyl acrylate (MA, 125 µL, 1.37 mmol, 170 equiv), TKLA (200 µL of 400 mg mL^{-1} stock solution in DMSO, 0.244 mmol, 30.0 equiv), DDMAT (28.3 µL of 105 mg mL^{-1} stock solution in DMSO, 8.07 µmol, 1.00 equiv), zinc tetraphenylporphyrin (ZnTPP, 40.8 µL of 1.35 mg mL^{-1} stock solution in DMSO, 0.081 µmol, 0.010 equiv), and ethylene carbonate (50 mg as an internal standard to determine monomer conversion) were added to a 2 mL HPLC vial, and diluted with DMSO (147 µL, [M]tot = 3.00 M). Prior to irradiation, the vial was vortexed for 20 s to homogenize the components, and a 10 µL aliquot was mixed with 600 µL acetone-d_6 to determine the initial monomer conversion (t = 0) by ^{1}H NMR spectroscopy. The polymerization mixture was then irradiated with yellow LEDs (595 nm, 7.0 mW cm^{-2}) for 3 h, and aliquots were taken at defined timepoints for analysis by ^{1}H NMR spectroscopy for monomer conversion and SEC with DMF + 50 mM LiBr as eluent for molar mass and dispersity (*Đ*). To purify the polymer, the reaction mixture was diluted with chloroform and precipitated into ice-cold diethyl ether : hexanes mixture (1:1 v/v) 3 times, and drying under high vacuum to yield a white solid ($M_{n,theory}$ = 15,300 g mol^{-1}, $M_{n,app}$ = 15,000 g mol^{-1}, *Đ* = 1.18).

P(TKLA-*co*-BA)

n-Butyl acrylate (BA, 186 µL, 1.29 mmol, 170 equiv), TKLA (200 µL of 400 mg mL^{-1} stock solution in DMSO, 0.230 mmol, 30.0 equiv), DDMAT (27.4 µL of 105 mg mL^{-1} stock solution in DMSO, 7.61 µmol, 1.00 equiv), zinc tetraphenylporphyrin (ZnTPP, 40.8 µL of 1.35 mg mL^{-1} stock solution in DMSO, 0.076 µmol, 0.010 equiv), and ethylene carbonate (50 mg as an internal standard to determine monomer conversion) were added to a 2 mL HPLC vial, and diluted with DMF (106 µL, [M]tot = 3.00 M). The vial was vortexed for 20 s to homogenize the components, and a 10 µL aliquot was mixed with 600 µL acetone-d_6 to determine the initial monomer conversion (t = 0) by ^{1}H NMR spectroscopy. The vial was then fitted with a rubber septum, and the reaction was sparged with argon for 5 min prior to being irradiated with yellow LEDs (595 nm, 7.0 mW cm^{-2}) for 8 h. Aliquots were taken using a syringe at defined timepoints for analysis by ^{1}H NMR spectroscopy for monomer conversion and SEC with DMF + 50 mM LiBr as eluent for molar mass and dispersity (*Đ*). To purify the polymer, the reaction mixture was diluted with acetone and dialyzed against acetone using a regenerated cellulose membrane (3.5 kDa molecular weight cutoff). After removal of solvent under vacuum, the polymer was obtained as a yellow-green solid ($M_{n,theory}$ = 29,900 g mol^{-1}, $M_{n,app}$ = 25,400 g mol^{-1}, *Đ* = 1.13).

P(TKLA-*co*-TEGA)

Tetraethylene glycol methyl ether acrylate (TEGA, 284 μL, 1.37 mmol, 170 equiv), TKLA (200 μL of 400 mg mL^{-1} stock solution in DMSO, 0.244 mmol, 30.0 equiv), DDMAT (28.3 μL of 105 mg mL^{-1} stock solution in DMSO, 8.07 μmol, 1.00 equiv), zinc tetraphenylporphyrin (ZnTPP, 40.8 μL of 1.35 mg mL^{-1} stock solution in DMSO, 0.081 μmol, 0.010 equiv), and ethylene carbonate (50 mg as an internal standard to determine monomer conversion) were added to a 2 mL HPLC vial. The final [M]tot was 2.92 M, and no additional DMSO was added. The vial was vortexed for 20 s to homogenize the components, and a 10 μL aliquot was mixed with 600 μL acetone-d_6 to determine the initial monomer conversion (t = 0) by ^{1}H NMR spectroscopy. The polymerization mixture was then irradiated with yellow LEDs (595 nm, 7.0 mW cm^{-2}) for 3 h, and aliquots were taken at defined timepoints for analysis by ^{1}H NMR spectroscopy for monomer conversion and SEC with DMF + 50 mM LiBr as eluent for molar mass and dispersity (*Đ*). To purify the polymer, the reaction mixture was diluted with chloroform and precipitated into ice-cold diethyl ether : hexanes mixture (1:1 v/v) 3 times and drying under high vacuum to yield a white solid ($M_{n,theory}$ = 26,000 g mol^{-1}, $M_{n,app}$ = 24,000 g mol^{-1}, *Đ* = 1.21).

P(TKLA-*co*-SBA)

Sulfobetaine acrylate (SBA, 233 mg, 0.550 mmol, 170 equiv), TKLA (80 μL of 400 mg mL^{-1} stock solution in DMSO, 0.100 mmol, 30.0 equiv), DDMAT (11.3 μL of 105 mg mL^{-1} stock solution in DMSO, 3.25 μmol, 1.00 equiv), zinc tetraphenylporphyrin (ZnTPP, 16.8 μL of 1.35 mg mL^{-1} stock solution in DMSO, 0.033 μmol, 0.010 equiv), and ethylene carbonate (50 mg as an internal standard to determine monomer conversion) were added to a 2 mL HPLC vial, and diluted wit 2,2,2-trifluoroethanol (TFE, 217 μL, [M]tot = 2.00 M). The vial was vortexed for 20 s to homogenize the components, and a 10 μL aliquot was mixed with 600 μL D_2O to determine the initial monomer conversion (t = 0) by ^{1}H NMR spectroscopy. The vial was then fitted with a rubber septum, and the reaction was sparged with argon for 10 min prior to being irradiated with yellow LEDs (595 nm, 7.0 mW cm^{-2}) for 1h. Aliquots were taken at defined timepoints for analysis by ^{1}H NMR spectroscopy for monomer conversion and SEC with DMF + 50 mM LiBr as eluent for molar mass and dispersity (*Đ*). To purify the polymer, the reaction mixture was diluted with deionized water and dialyzed against deionized water using a regenerated cellulose membrane (3.5 kDa molecular weight cutoff). After lyophilization, the polymer was obtained as a white solid ($M_{n,theory}$ = 49,300 g mol^{-1}, $M_{n,abs}$ = 34,900 g mol^{-1}, *Đ* = 1.28).

Typical functionalization of P(TKLA-*co*-TK2Am) and P(TKLA-*co*-TK5Am) with amines resulting in P(DKE-R-amine) (see figure 4 in main text)

To a solution of P(TKLA-*co*-TK2Am) (40 mg, 129 μmol TK groups, 1.00 equiv) or P(TKLA-*co*-TK5Am) (40 mg, 124 μmol TK groups, 1.00 equiv) in 1.0 mL chloroform, was added the amine (2.00 equiv) and stirred for 16 h at room temperature. Note: the reactions also went to full conversion with 1.2 equiv of amine. When functionalizing with *N,N*-dimethyl-ethylene diamine (DMED), ethanolamine, mPEG-NH_2, and tryptamine, DMF was used in lieu of chloroform for better solubility of the amine and the resulting P(DKE) polymer.

To purify most P(DKE) polymers, the reaction solution was precipitated into ice-cold diethyl ether. Precipitation two times into ice-cold pentane was used instead for P(DKE-hexyl) and P(DKE-cyclohexyl).

P(DKE-DMED) was purified via dialysis against methanol acidified with HCl using a regenerated cellulose membrane (3.5 kDa molecular weight cutoff) to protonate the tertiary amine pendant groups.

PEG-bottlebrush polymer (PEG-BB) was purified via dialysis against deionized water using a regenerated cellulose membrane (50 kDa molecular weight cutoff).

Degradation of PEG-BB using TCEP

To 4 mL shell vial, PEG-BB (10 mg) was dissolved in DMF (0.75 mL), followed by the addition of deionized water (0.25 mL). TCEP was added (29 mg, [TCEP] = 100 mM) and the mixture was vortexed until all solids dissolved. The reaction was stirred at rt 25 °C for 24 h. For SEC analysis, a 100 µL aliquot was taken and diluted with 1.5 mL DMF, then centrifuged for 5 min to remove undissolved TCEP before injection into the SEC.

Degradation of PEG-BB using UV light

To 4 mL shell vial, PEG-BB (10 mg) was dissolved in DMF (1.0 mL), followed by the addition of deionized water (0.25 mL). The reaction mixture was then irradiated with UV LEDs (370 nm, 7.0 mW cm^{-2}) for 24 h. A 100 µL aliquot was taken and diluted with 1.5 mL THF for analysis by SEC.

Functionalization of HMW P(TKLA-*co*-DMA) and P(TKLA-*co*-MA)

HMW P(DKE-benzyl)

To a solution of HMW P(TKLA-*co*-MA) (20 mg, 29.8 µmol TK groups, 1.00 equiv; $M_{n,app}$ = 426,000 g mol^{-1}, $Đ$ = 1.44) in 1.00 mL chloroform, was added benzylamine (6.50 µL, 59.6 µmol, 2.00 equiv), and the reaction was stirred for 16 h. To purify the polymer, the reaction mixture was diluted with chloroform and precipitated into ice-cold diethyl ether : hexanes mixture (1:1 v/v) 3 times and drying under high vacuum to yield a white solid ($M_{n,app}$ = 435,000 g mol^{-1}, $Đ$ = 1.40).

HMW P(DKE-mPEG3)

To a solution of HMW P(TKLA-*co*-MA) (30 mg, 29.1 µmol TK groups, 1.00 equiv; $M_{n,app}$ = 532,000 g mol^{-1}, $Đ$ = 1.66) in 1.00 mL chloroform, was added 2-[2-(2-methoxyethoxy)ethoxy]ethylamine (mPEG3-amine, 15.3 µL, 58.2 µmol, 2.00 equiv), and the reaction was stirred for 16 h. To purify the polymer, the reaction mixture was diluted with chloroform and precipitated into ice-cold diethyl ether : hexanes mixture (1:1 v/v) 3 times and drying under high vacuum to yield a white solid ($M_{n,app}$ = 637,000 g mol^{-1}, $Đ$ = 1.72).

HMW P(DKE-nitrodopamine)

To a solution of HMW P(TKLA-*co*-DMA) (40 mg, 57.9 µmol TK groups, 1.00 equiv; $M_{n,app}$ = 396,000 g mol^{-1}, $Đ$ = 1.67) in 2.00 mL DMSO, was added triethylamine (41.2 µL, 174 µmol, 3.00 equiv) and 6-nitrodopamine hemisulfate (143 mg, 57.9 µmol, 2.00 equiv amine), and the reaction was stirred for 16 h. To purify the polymer, the reaction mixture was diluted with DMSO and dialyzed against deionized water using a regenerated cellulose membrane (50 kDa molecular weight cutoff) yielding a brown solid.

Degradation of HMW P(DKE-mPEG3) with TCEP

To 4 mL shell vial, HMW P(DKE-mPEG3) (10 mg) was dissolved in DMF (0.75 mL), followed by the addition of deionized water (0.25 mL). TCEP was added (29 mg, [TCEP] = 100 mM) and the mixture was vortexed until all solids dissolved. The reaction was stirred and heated in an oil bath at 50 °C for 48 h. For SEC analysis, a 100 µL aliquot was taken and diluted with 1.5 mL DMF, then centrifuged for 5 min to remove undissolved TCEP before injection into the SEC.

Synthesis of low molecular weight (LMW) P(TKLA-*co*-DMA) macroinitiator

DMA (587 µL, 5.69 mmol, 85 equiv), TKLA (660 µL of 500 mg mL^{-1} stock solution in DMSO, 1.00 mmol, 15.0 equiv), DDMAT (233 µL of 105 mg mL^{-1} stock solution in DMSO, 67.0 µmol, 1.00 equiv), zinc tetraphenylporphyrin (ZnTPP, 336 µL of 1.35 mg mL^{-1} stock solution in DMSO, 0.670 µmol, 0.010 equiv), and ethylene carbonate (50 mg as an internal standard to determine monomer conversion) were added to a 4 mL shell vial, and diluted with DMSO (416 µL, [M]tot = 3.00 M). Prior to irradiation, the vial was vortexed for 20 s to homogenize the components, and a 15 µL aliquot was mixed with 600 µL acetone-d_6 to determine the initial monomer conversion (t = 0) by ^{1}H NMR spectroscopy. The polymerization mixture was then irradiated with yellow LEDs (595 nm, 7.0 mW cm^{-2}) for 1 h, reaching 62 % conversion for DMA and 73 % for TKLA. The polymerization was stopped early to preserve chain-end fidelity. To purify the polymer, the viscous solution was diluted with chloroform and precipitated into ice-cold diethyl ether three times and finally dried under high vacuum resulting in a green powder ($M_{n,theory}$ = 8,820 g mol^{-1}, $M_{n,app}$ = 7,880 g mol^{-1}, $Đ$ = 1.15).

Chain extension of P(TKLA-*co*-DMA) with MA

MA (288 µL, 3.17 mmol, 500 equiv), P(TKLA-*co*-DMA) (50.0 mg, 6.35 µmol, 1.00 equiv; $M_{n,app}$ = 7,880 g mol^{-1}, $Đ$ = 1.15), zinc tetraphenylporphyrin (ZnTPP, 38.4 µL of 1.12 mg mL^{-1} stock solution in DMSO, 6.35 $\times$ 10^{-2} µmol, 0.010 equiv), and ethylene carbonate (50 mg as an internal standard to determine monomer conversion) were added to a 2 mL HPLC vial, and diluted with DMSO (1.21 mL, [M]tot = 2.00 M). Prior to irradiation, the vial was vortexed for 20 s to homogenize the components, and a 15 µL aliquot was mixed with 600 µL acetone-d_6 to determine the initial monomer conversion (t = 0) by ^{1}H NMR spectroscopy. The polymerization mixture was then irradiated with yellow LEDs (595 nm, 7.0 mW cm^{-2}) for 30 min, reaching 68% conversion. To purify the polymer, the viscous solution was diluted with chloroform and precipitated into ice-cold diethyl ether : hexanes mixture (1:1 v/v) 3 times and finally dried under high vacuum resulting in a light-yellow powder ($M_{n,theory}$ = 37,500 g mol^{-1}, $M_{n,app}$ = 44,500 g mol^{-1}, $Đ$ = 1.11).

Chain extension of P(TKLA-*co*-DMA) with NAM

NAM (400 µL, 3.17 mmol, 500 equiv), P(TKLA-*co*-DMA) (50.0 mg, 6.35 µmol, 1.00 equiv; $M_{n,app}$ = 7,880 g mol^{-1}, $Đ$ = 1.15), zinc tetraphenylporphyrin (ZnTPP, 38.4 µL of 1.12 mg mL^{-1} stock solution in DMSO, 6.35 $\times$ 10^{-2} µmol, 0.010 equiv), and ethylene carbonate (50 mg as an internal standard to determine monomer conversion) were added to a 2 mL HPLC vial, and diluted with DMSO (1.10 mL, [M]tot = 2.00 M). Prior to irradiation, the vial was vortexed for 20 s to homogenize the components, and a 15 µL aliquot was mixed with 600 µL acetone-d_6 to determine the initial monomer conversion (t = 0) by ^{1}H NMR spectroscopy. The polymerization mixture was then irradiated with yellow LEDs (595 nm, 7.0 mW cm^{-2}) for 30 min, reaching 89% conversion. To purify the polymer, the viscous solution was diluted with chloroform and precipitated into ice-cold diethyl ether : hexanes mixture (1:1 v/v) 3 times and finally dried under high vacuum resulting in a light-yellow powder ($M_{n,theory}$ = 71,500 g mol^{-1}, $M_{n,app}$ = 81,500 g mol^{-1}, $Đ$ = 1.14).

Chain extension of P(TKLA-*co*-TK2Am) with DMA

DMA (238 µL, 2.31 mmol, 500 equiv), P(TKLA-*co*-TK2Am) (111 mg, 4.63 µmol, 1.00 equiv; $M_{n,app}$ = 24,400 g mol^{-1}, $Đ$ = 1.30), zinc tetraphenylporphyrin (ZnTPP, 23.2 µL of 1.35 mg mL^{-1} stock solution in DMSO, 4.63 $\times$ 10^{-2} µmol, 0.010 equiv), and ethylene carbonate (50 mg as an internal standard to determine monomer conversion) were added to a 2 mL HPLC vial, and diluted with DMSO (845 µL, [M]tot = 2.00 M). Prior to irradiation, the vial was vortexed for 20 s to homogenize the components, and a 15 µL aliquot was mixed with 600 µL acetone-d_6 to determine the initial monomer conversion (t = 0) by ^{1}H NMR spectroscopy. The polymerization mixture was then irradiated with yellow LEDs (595 nm, 7.0 mW cm^{-2}) for 15 min, reaching 35% conversion. To purify the polymer, the viscous solution was diluted with chloroform and precipitated

into ice-cold diethyl ether 3 times and finally dried under high vacuum resulting in a white powder ($M_{n,theory}$ = 41, 700 g mol^{-1}, $M_{n,app}$ = 51, 800 g mol^{-1}, $Đ$ = 1.24).

Functionalization of P(TKLA-*co*-TK2Am)-*b*-PDMA with *n*-hexylamine

P(TKLA-*co*-TK2Am)-*b*-PDMA (40 mg, 93.1 µmol TK groups, 1.00 equiv; $M_{n,app}$ = 51, 800 g mol^{-1}, $Đ$ = 1.24) was dissolved in 1 mL of chloroform. *n*-Hexylamine (24.6 µL, 186 µmol, 2.00 equiv) was added, and the reaction was allowed to stir for 5 h. The polymer was purified via precipitation into ice-cold pentane 2 times and finally dried under high vacuum resulting in a white powder.

Self-assembly of P(DKE-hexyl)-*b*-PDMA

First, a stock solution of P(DKE-hexyl)-*b*-PDMA was prepared in DMSO (2.00 mg mL^{-1}). 100 µL of the stock solution were then added dropwise into HPLC-grade water (1.90 mL) under strong stirring, to reach a final polymer concentration of 100 µg/mL. After stirring for an additional 15 min, the formed micelles were characterized by DLS.

Synthesis of PEG-*b*-P(TKLA-*co*-TK2Am)

TK2Am (612 mg, 2.31 mmol, 160 equiv), TKLA (378 µL of 500 mg mL^{-1} stock solution in DMSO, 0.575 mmol, 40.0 equiv), 2-(dodecylthiocarbonothioylthio)-2-methylpropionate (PEG-TTC, 87.0 mg, 14.4 µmol, 1.00 equiv; $M_{n,app}$ = 6,000 g mol^{-1}, $Đ$ = 1.09), ZnTPP (78 µL of 1.35 mg mL^{-1} stock solution in DMSO, 0.014 µmol, 0.010 equiv), and ethylene carbonate (50 mg as an internal standard to determine monomer conversion) were added to a 2 mL HPLC vial, and diluted with DMSO (419 µL, [M]tot = 3.00 M). Prior to irradiation, the vial was vortexed for 20 s to homogenize the components, and a 15 µL aliquot was mixed with 600 µL acetone-d_6 to determine the initial monomer conversion (t = 0) by ^{1}H NMR spectroscopy. The polymerization mixture was then irradiated with yellow LEDs (595 nm, 7.0 mW cm^{-2}) for 2 h, reaching 23% conversion for TK2Am and 33% for TKLA. To purify the polymer, the viscous solution was diluted with chloroform and precipitated into ice-cold diethyl ether three times and finally dried under high vacuum resulting in a yellow powder ($M_{n,theory}$ = 20,100 g mol^{-1}, $M_{n,app}$ = 21,000 g mol^{-1}, $Đ$ = 1.29).

Functionalization of PEG-*b*-P(TKLA-*co*-TK2Am) with *n*-hexylamine

PEG-*b*-P(TKLA-*co*-TK2Am) (42 mg, 100 µmol TK groups, 1.00 equiv; $M_{n,app}$ = 21, 000 g mol^{-1}, $Đ$ = 1.29) was dissolved in 1 mL chloroform. *n*-Hexylamine (26.4 µL, 200 µmol, 2.00 equiv) was added, and the reaction was allowed to stir for 5 h. The polymer was purified via precipitation into ice-cold pentane 2 times and finally dried under high vacuum resulting in a white powder.

Self-assembly of PEG-*b*-P(TKLA-*co*-TK2Am)

First, a stock solution of PEG-*b*-P(TKLA-*co*-TK2Am) was prepared in DMSO (2.00 mg mL^{-1}). 200 µL of the stock solution were then added dropwise into HPLC-grade water (1.80 mL) under strong stirring, to reach a final polymer concentration of 200 µg/mL. After stirring for an additional 15 min, the formed micelles were characterized by DLS.

Self-assembly of PEG-*b*-P(DKE-hexyl)

First, a stock solution of PEG-*b*-P(DKE-hexyl) was prepared in DMSO (2.00 mg mL^{-1}). 100 µL of the stock solution were then added dropwise into HPLC-grade water (1.90 mL) under strong stirring, to reach a final polymer concentration of 100 µg/mL. After stirring for an additional 15 min, the formed micelles were characterized by DLS.

Supplementary Tables

Table S1. Copolymerization of TKLA with TK2Am at varying degrees of polymerization.[a]

Entry	Time (h)	DP_t	TK2Am Conv. (%)[b]	TKLA Conv. (%)[b]	$M_{n,theory}$ (kg mol^{-1})[c]	$M_{n,app}$ (kg mol^{-1})[d]	$Đ$[d]
1	3.0	100	37	57	11.2	8.97	1.29
2	2.0	200	64	76	36.8	30.4	1.36
3	2.0	500	62	78	72.7	52.6	1.57

[a]Reaction conditions: [TK2Am]/[TKLA]/[DDMAT]/[ZnTPP] = 0.85x/0.15y/1/0.01, $[M]_{tot}$ = 3.0 M, irradiated under 595 nm LED (7.0 mW cm^{-2}) in DMSO solvent at rt. [b]Monomer conversions determined by 1H NMR spectroscopy. [c]Theoretical number-average molar masses (Mn,theory) were determined from the monomer conversion. [e]Apparent number-average molar masses ($M_{n,app}$) and dispersity ($Đ$) were determined by SEC using DMF + 50 mM LiBr relative to PMMA standards.

Table S2. Vinyl comonomer scope[a]

Entry	Comonomer	Solvent	Time (h)	Total [M]	Vinyl Conv. (%)[c]	TKLA Conv. (%)[c]	$M_{n,theory}$ (kg mol^{-1})[d]	$M_{n,app}$ (kg mol^{-1})[d]	$M_{n,abs}$ (kg mol^{-1})[e]	$Đ$[e,f]
1	Am	DMSO	3.0	4.0	85	97	20.0	8.70	-	1.25
2	DMA	DMSO	3.0	3.0	93	92	24.2	24.7	-	1.14
3	NAM	DMSO	2.0	3.0	98	99	25.3	33.3	-	1.20
4	NiPAM	DMSO	2.0	3.0	79	98	28.2	24.8	-	1.16
5	HEAm	DMSO	2.0	3.0	81	92	24.9	24.9	-	1.19
6	BiBAm[b]	DMSO	2.0	2.0	71	90	40.7	22.8		1.30
7	CBAm[b]	TFE	1.0	2.0	89	>99	40.7	-	53.7	1.28
8	DMEQAAm	DMSO	2.0	2.0	77	92	41.0	-	13.6	1.44
9	TK2Am	DMSO	2.0	3.0	55	69	31.5	24.0	-	1.32
10	TK5Am[b]	DMSO	4.0	3.0	49	77	33.2	25.0	-	1.27
11	Am-Phe-OMe	DMSO	8.0	1.5	92	>99	46.5	37.9	-	1.70
12	Am-Phe-Ala-OMe	DMSO	8.0	1.4	86	>99	54.4	43.1	-	1.65
13	MA	DMSO	3.0	3.0	52	78	15.3	15.0	-	1.18
14	BA[b]	DMSO/DMF	8.0	3.0	92	>99	29.9	25.4	-	1.13
15	TEGA	DMSO	2.0	3.0	53	64	26.0	24.6	-	1.21
16	SBA[b]	TFE	1.0	2.0	89	99	49.3		34.9	1.24

[a]Reaction conditions: [DMA]/[TKLA]/[DDMAT]/[ZnTPP] = 170/30/1/0.01, irradiated under 595 nm LED (7.0 mW cm^{-2}) at rt. [b] Polymerization was run after deoxygenation via sparging with argon for 15 min and sealing the vial with a septum. [c] Monomer conversions determined by 1H NMR spectroscopy. [d]Theoretical number-average molar masses ($M_{n,theory}$) were determined from the monomer conversion. [e]Apparent number-average molar masses ($M_{n,app}$) and dispersity ($Đ$) were determined by SEC using DMF + 50 mM LiBr relative to PMMA standards. [f]For copolymers not soluble in DMF, aqueous SEC was used; absolute number-average molar masses ($M_{n,abs}$) and $Đ$ were determined by SEC-MALS using refractive index increment (dn/dc) measured offline in the corresponding aqueous buffer.

Table S3. Copolymerization of TKLA with MA targeting high degrees of polymerization.[a]

Entry	Time (h)	DP_t	MA Conv. (%)[b]	TKLA Conv. (%)[b]	$M_{n,theory}$ (kg mol^{-1})[c]	$M_{n,app}$ (kg mol^{-1})[d]	$Đ$[d]
1	2.0	2,000	19	63	71.5	69.1	1.29
2	2.0	5,000	53	87	328	242	1.32
3	2.0	10,000	49	77	633	426	1.44
4	4.0	20,000	41	62	1,040	532	1.66

[a]Reaction conditions: [MA]/[TKLA]/[DDMAT]/[ZnTPP] = 0.90x/0.10y/1/0.01, irradiated under 595 nm LED (2.0 mW cm^{-2}) in DMSO solvent at –20 °C, and deoxygenated via sparging with argon for 10 min. [b]Monomer conversions determined by 1H NMR spectroscopy. [c]Theoretical number-average molar masses ($M_{n,theory}$) were determined from the monomer conversion. [d]Apparent number-average molar masses ($M_{n,app}$) and dispersity ($Đ$) were determined by SEC using DMF + 50 mM LiBr relative to PMMA standards.

Table S4. Self-Assembly Data for amphiphilic block copolymers.

Copolymer	D_n(intensity) (nm)	D_n(number) (nm)	D_n(volume) (nm)	Z-average (nm)	PDI
P(DKE-hexyl)-*b*-PDMA	117	65	91	103	0.209
PEG-*b*-P(TKLA-*co*-TK2Am)	47	34	39	46	0.176
PEG-*b*-P(DKE-hexyl)	89	46	62	78	0.196
PEG-*b*-P(DKE-hexyl) after 3 months	81	46	60	70	0.135

Supplementary Figures

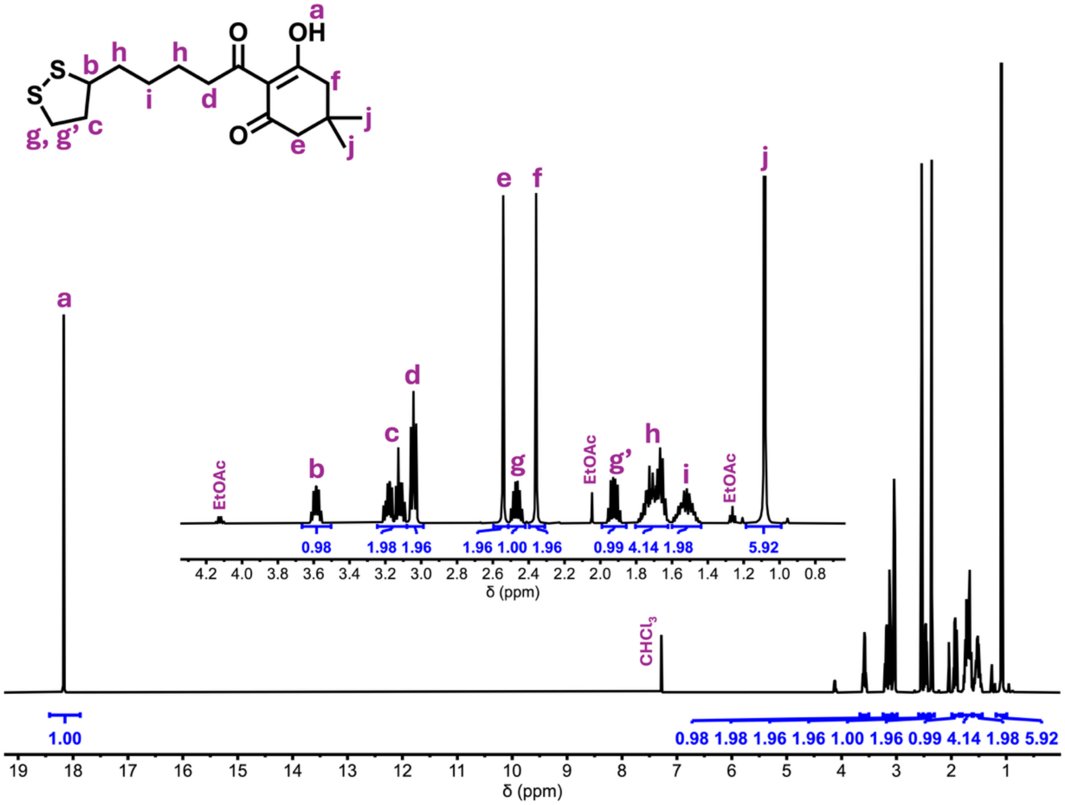

Figure S1. ^{1}H NMR spectrum of TKLA, recorded in $CDCl_3$.

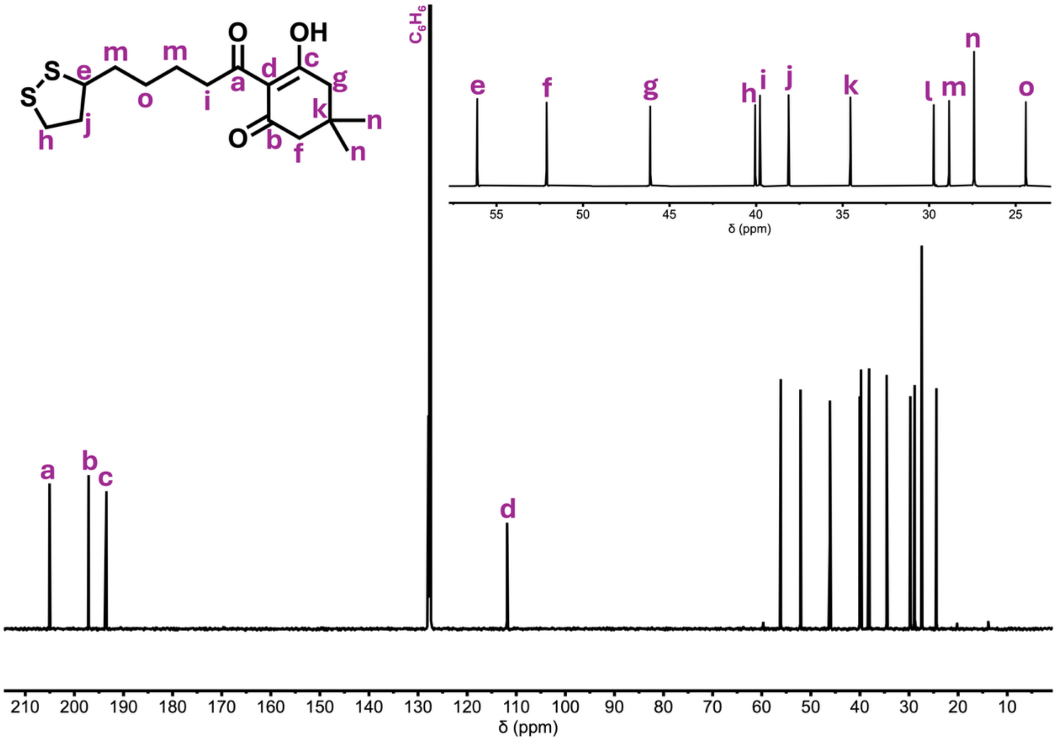

Figure S2. ^{13}C NMR of TKLA, recorded in benzene-d_6.

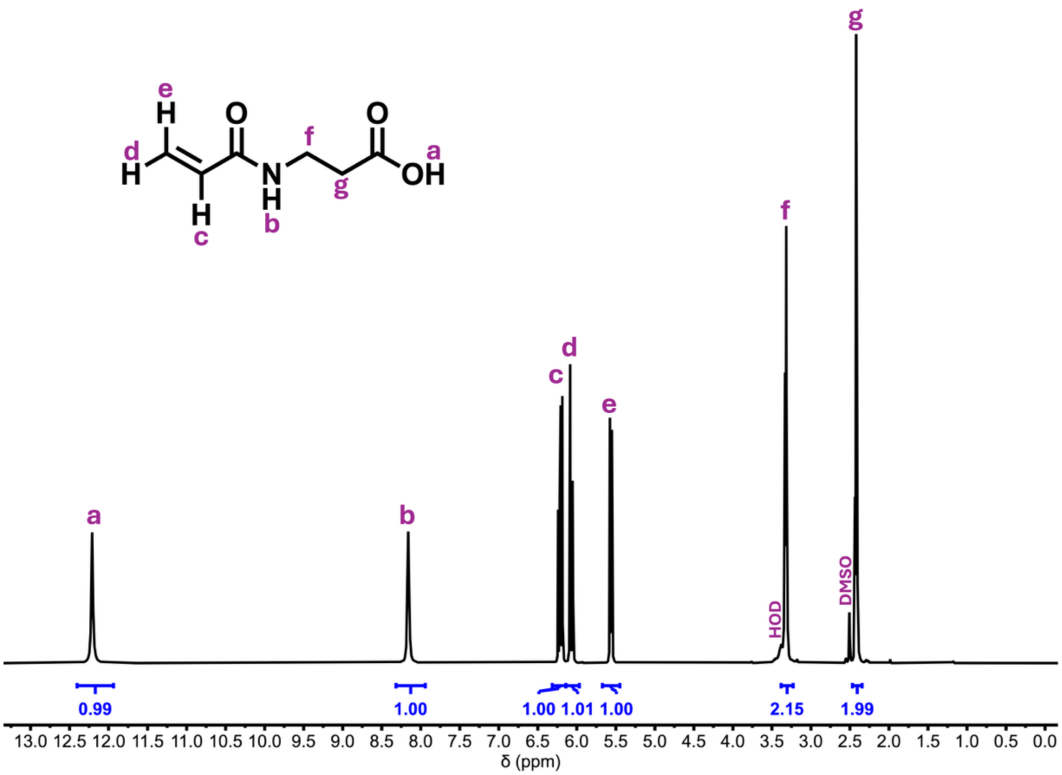

Figure S3. ^{1}H NMR of 3-acrylamidopropanoic acid, recorded in DMSO-d_6.

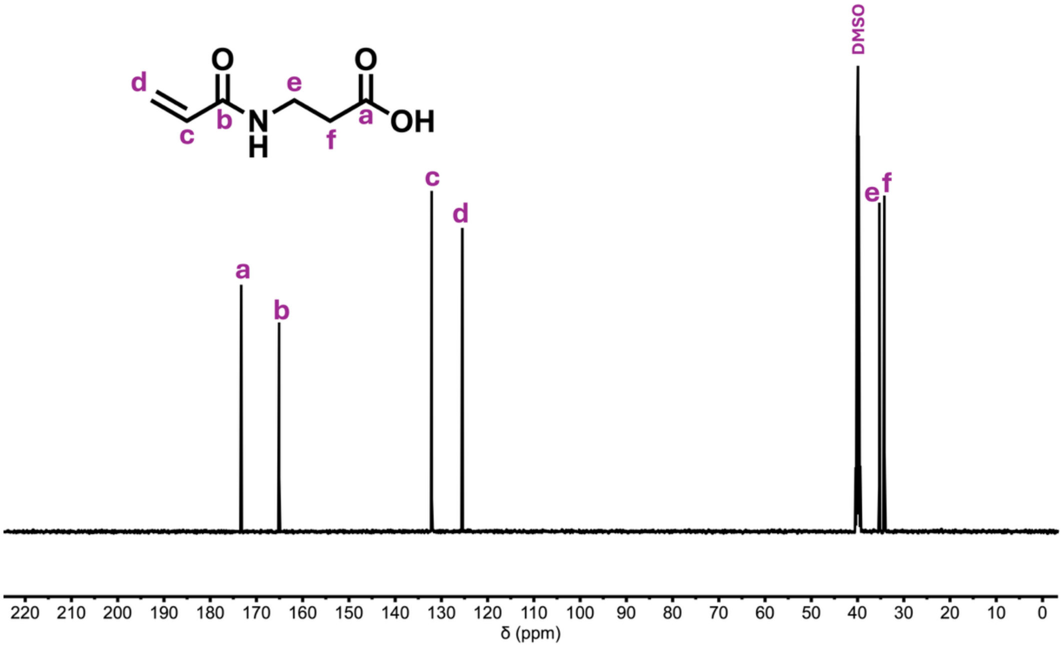

Figure S4. ^{13}C NMR of 3-acrylamidopropanoic acid, recorded in DMSO-d_6.

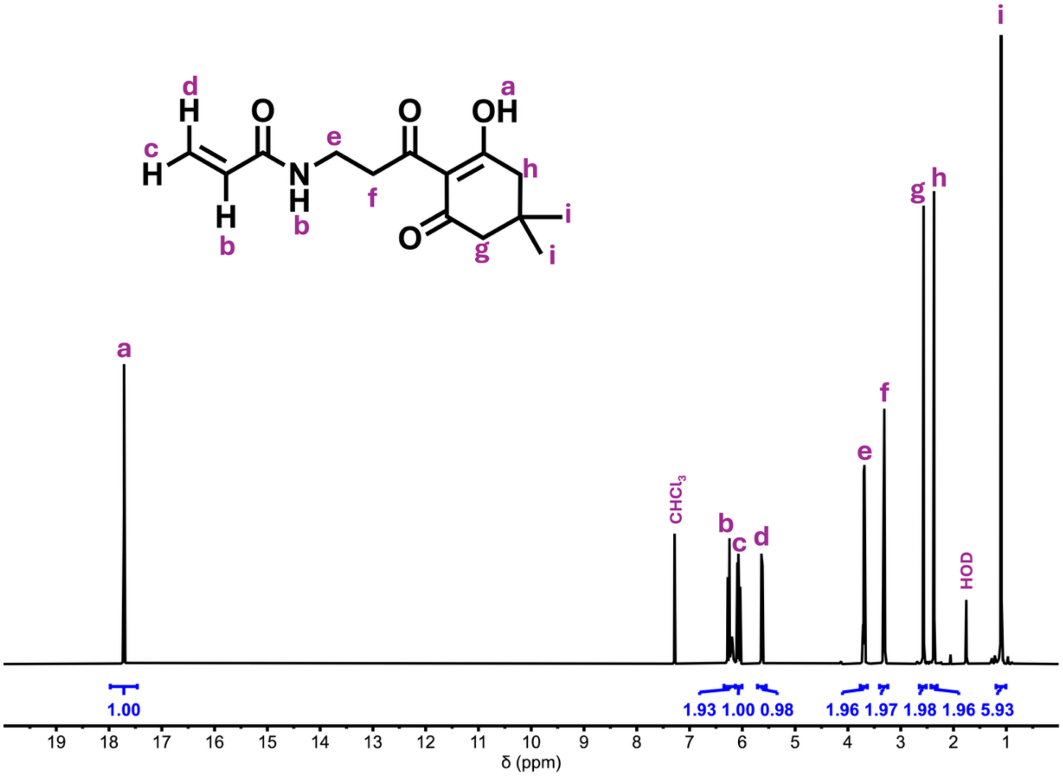

Figure S5. ^{1}H NMR of TK2Am, recorded in $CDCl_3$.

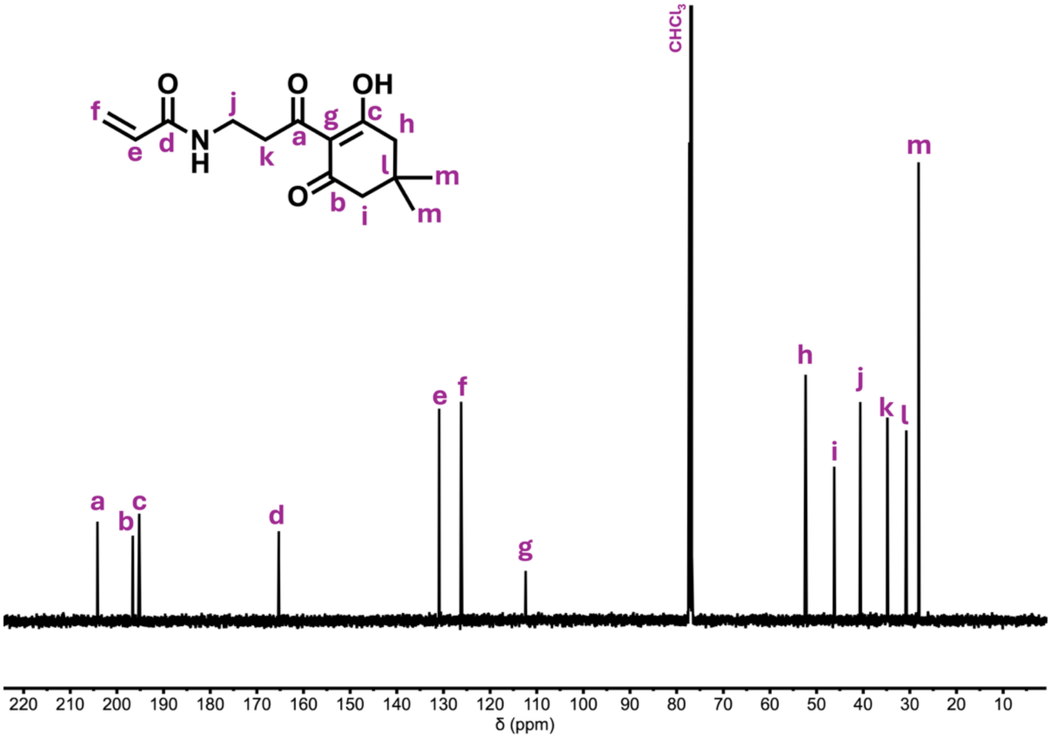

Figure S6. ^{13}C NMR of TK2Am, recorded in $CDCl_3$.

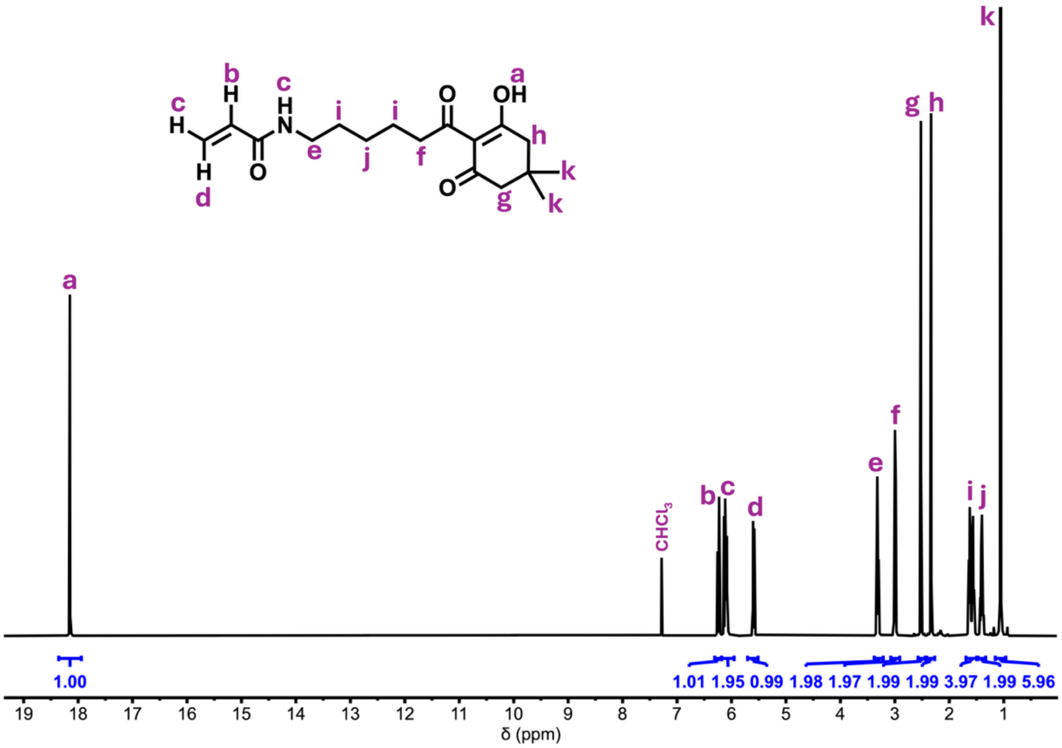

Figure S7. ^{1}H NMR of TK5Am, recorded in $CDCl_3$.

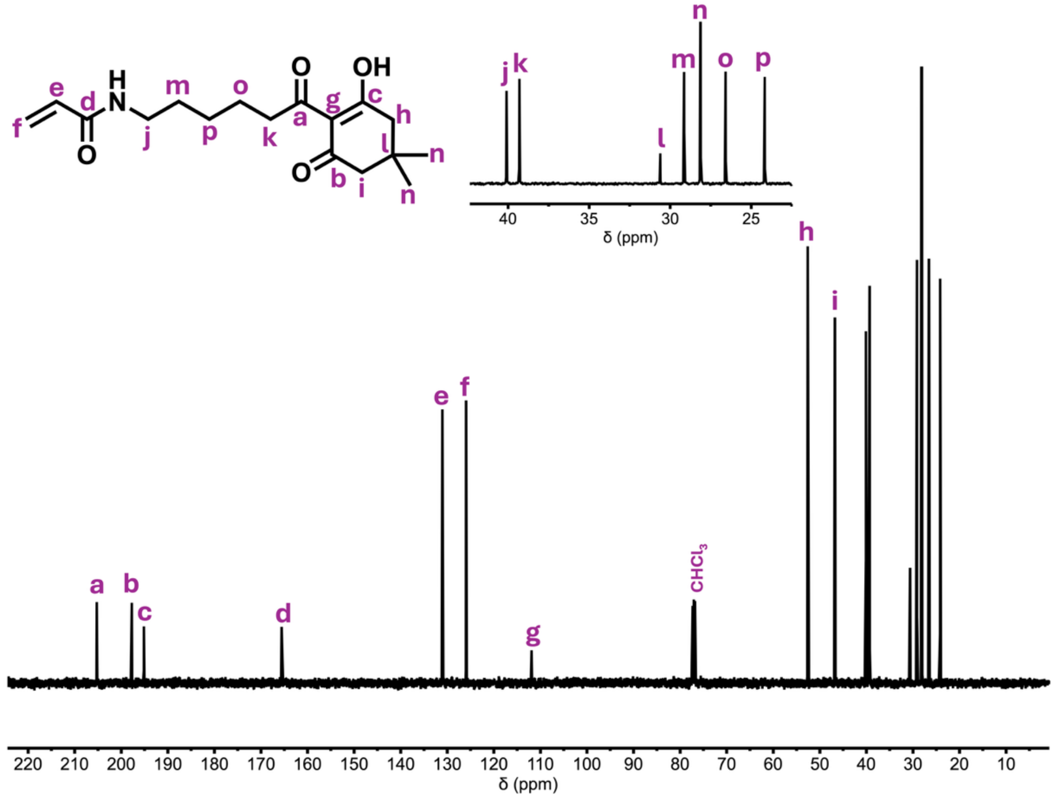

Figure S8. ^{13}C NMR of TK5Am, recorded in $CDCl_3$.

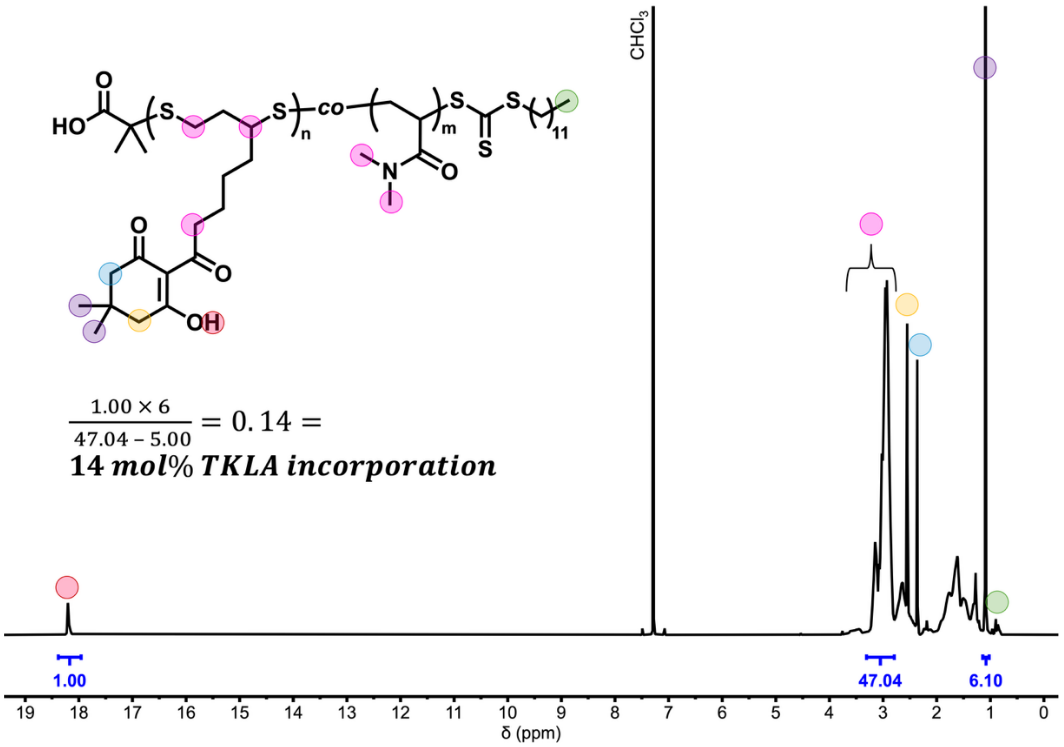

Figure S9. ^{1}H NMR of P(TKLA-*co*-DMA), recorded in $CDCl_3$.

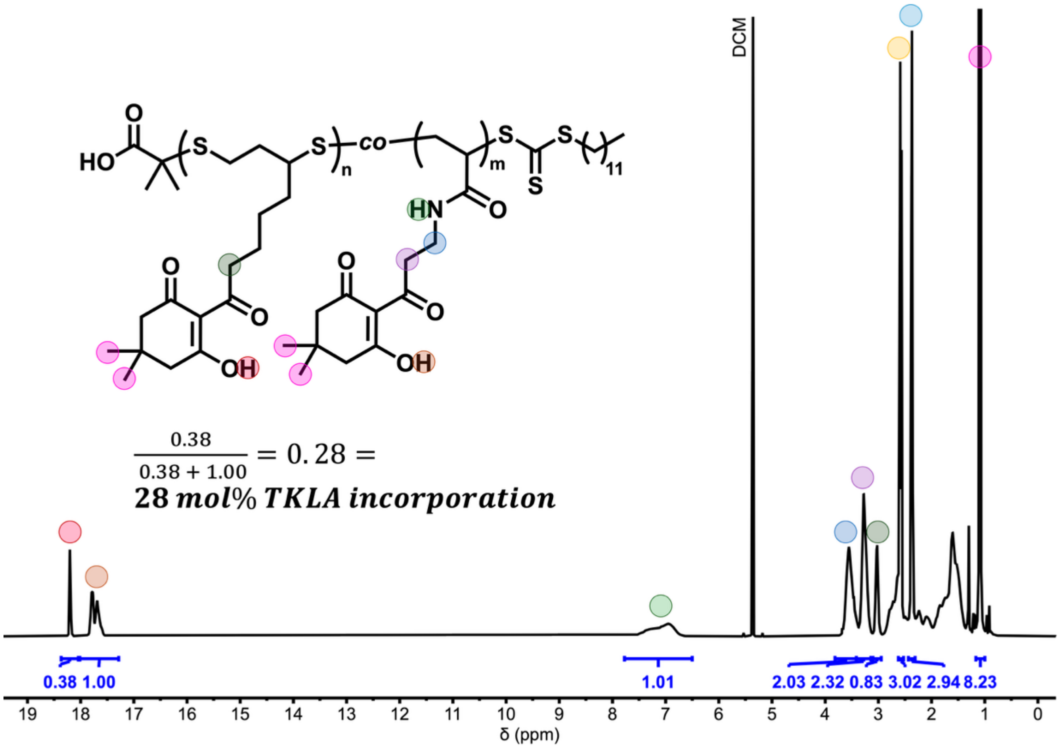

Figure S10. ^{1}H NMR of P(TKLA-*co*-TK2Am), recorded in DCM-d_2.

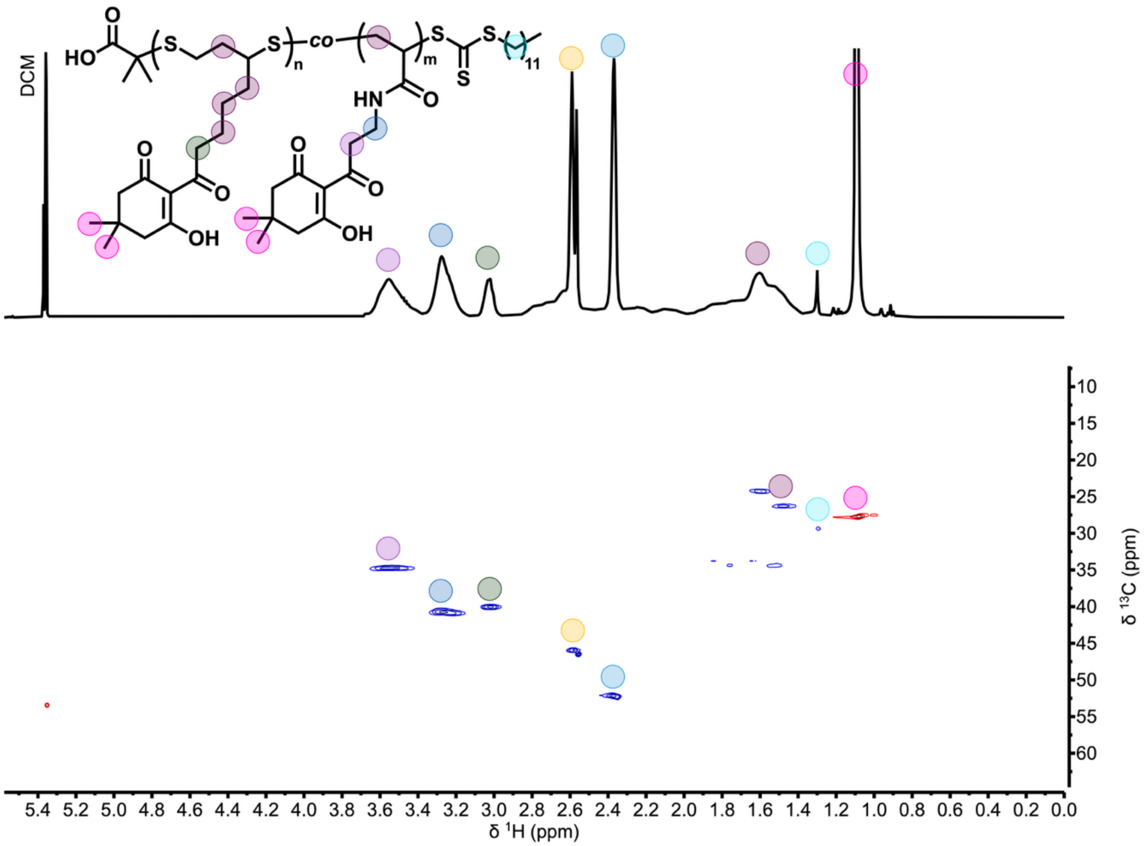

Figure S11. 2D ^{1}H-^{13}C heteronuclear single-quantum correlation (HSQC) NMR spectrum of P(TKLA-*co*-TK2Am), recorded in DCM-d_2.

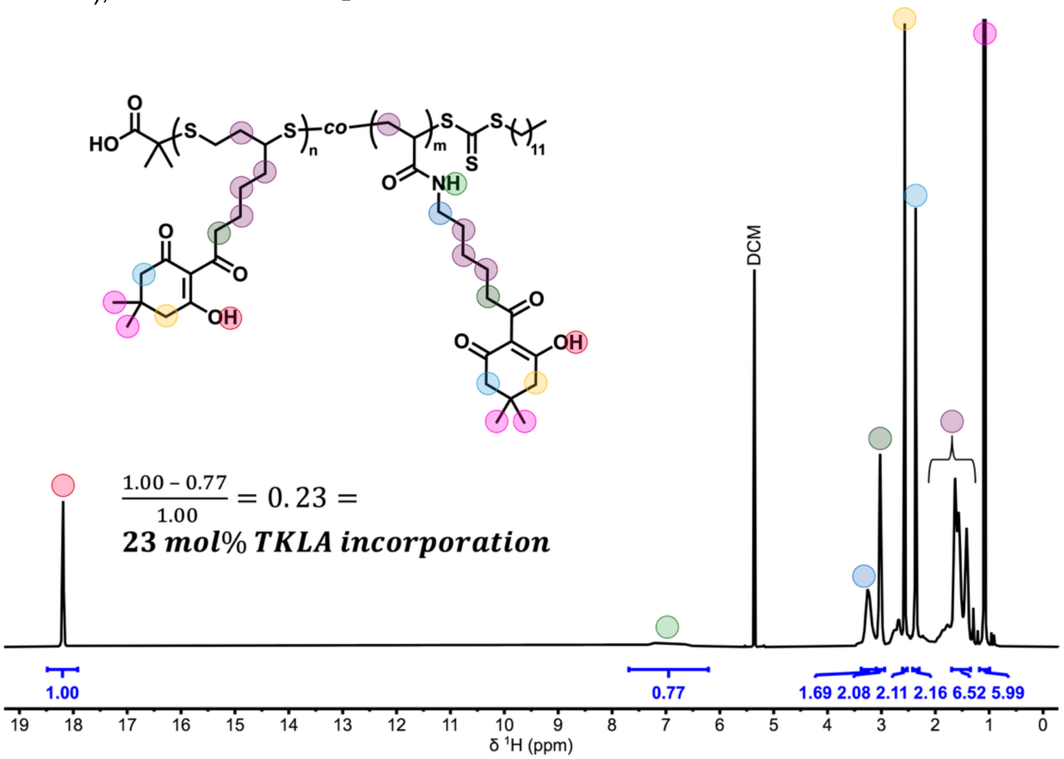

Figure S12. ^{1}H NMR of P(TKLA-*co*-TK5Am), recorded in DCM-d_2.

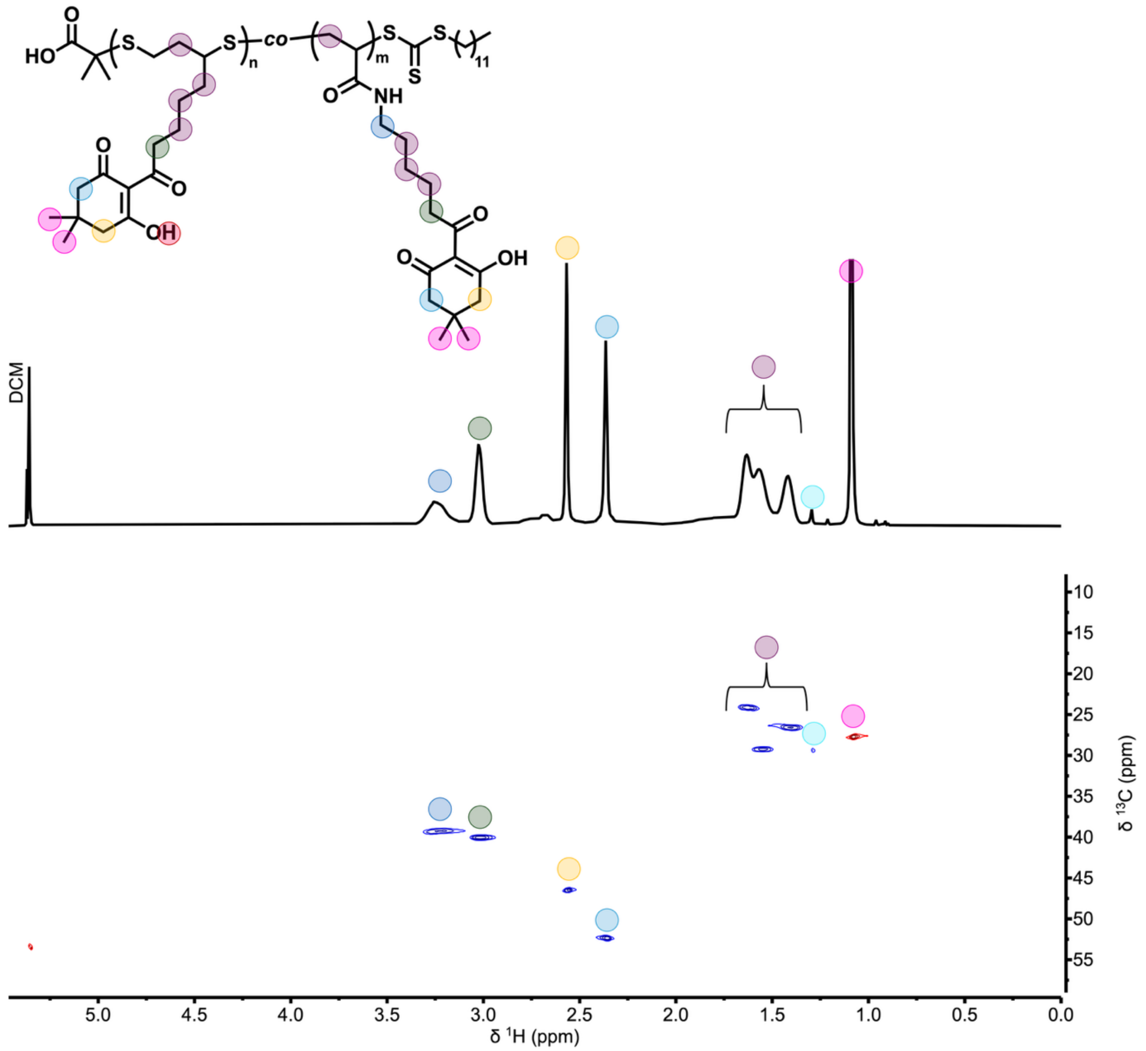

Figure S13. 2D ^{1}H-^{13}C HSQC NMR of P(TKLA-*co*-TK2Am), recorded in DCM-d_2.

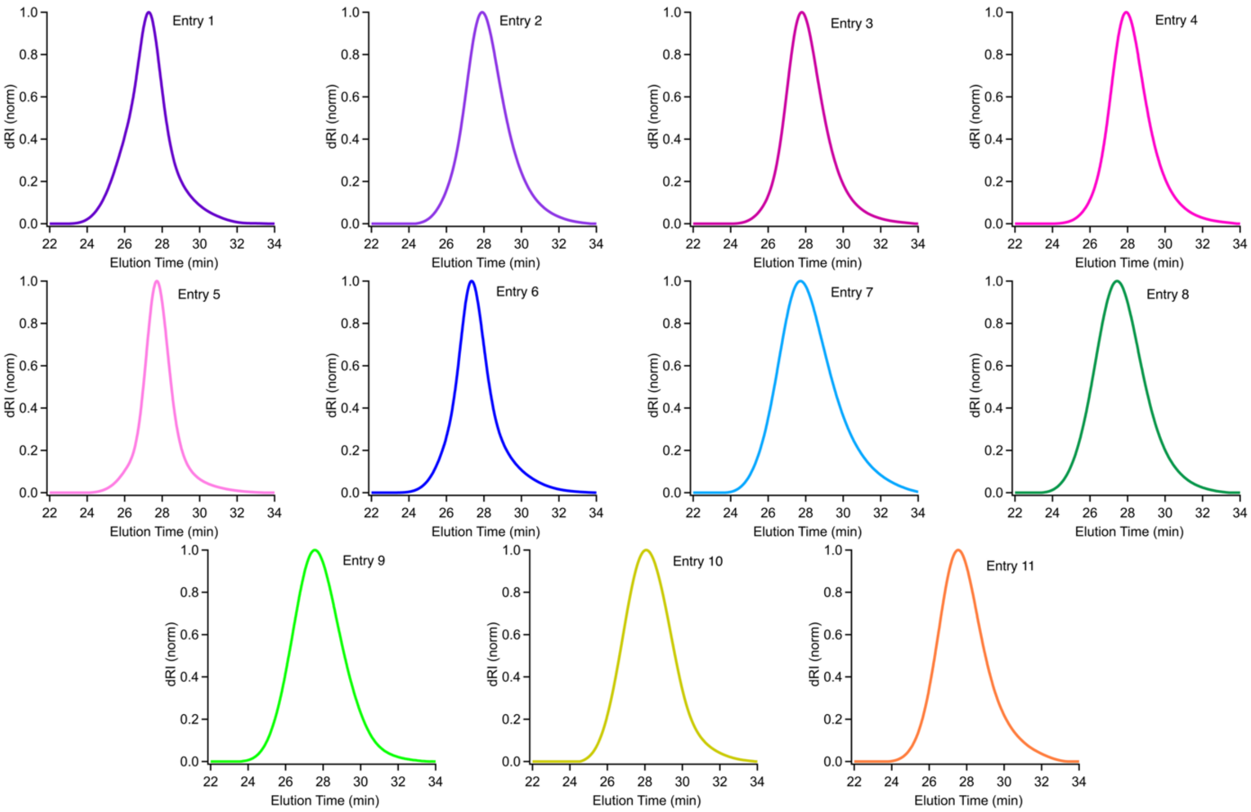

Figure S14. SEC traces corresponding to Table 1: Initial Screening of Polymerization Conditions.

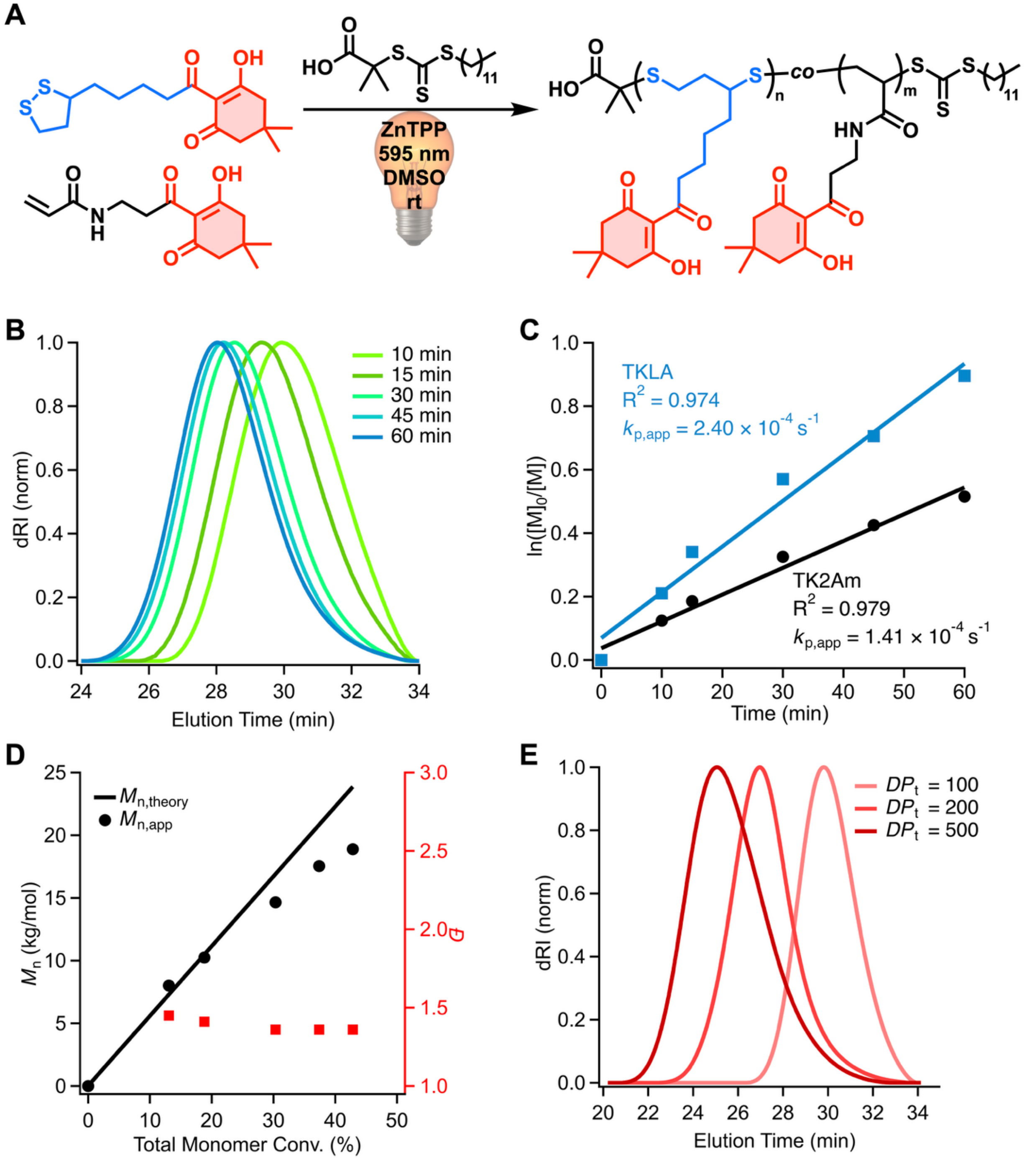

Figure S15. (A) Reaction scheme for the PET-RAFT copolymerization of TKLA with TK2Am. Reaction conditions: [TK2Am]/[TKLA/[DDMAT]/[ZnTPP] = 170/30/1/0.01. (B) Evolution of SEC traces of P(TKLA-*co*-TK2Am) taken over the course of polymerization, showing timepoints from 0 to 60 min. (C) Pseudo first-order kinetic plot for TKLA and TK2Am conversion. Apparent rate constants: $k_{p,app}$ = $(2.40 \pm 0.01) \times 10^{-4}$ s^{-1} for TKLA (R^2 = 0.974), and $k_{p,app}$ = $(2.62 \pm 0.01) \times 10^{-4}$ s^{-1} for TK2Am (R^2 = 0.979). (D) P(TKLA-*co*-TK2Am) $M_{n,app}$ versus total monomer conversion showing good agreement with $M_{n,theory}$ while maintaining low *Đ*. (E) SEC traces of P(TKLA-*co*-TK2Am) with different DP_t values.

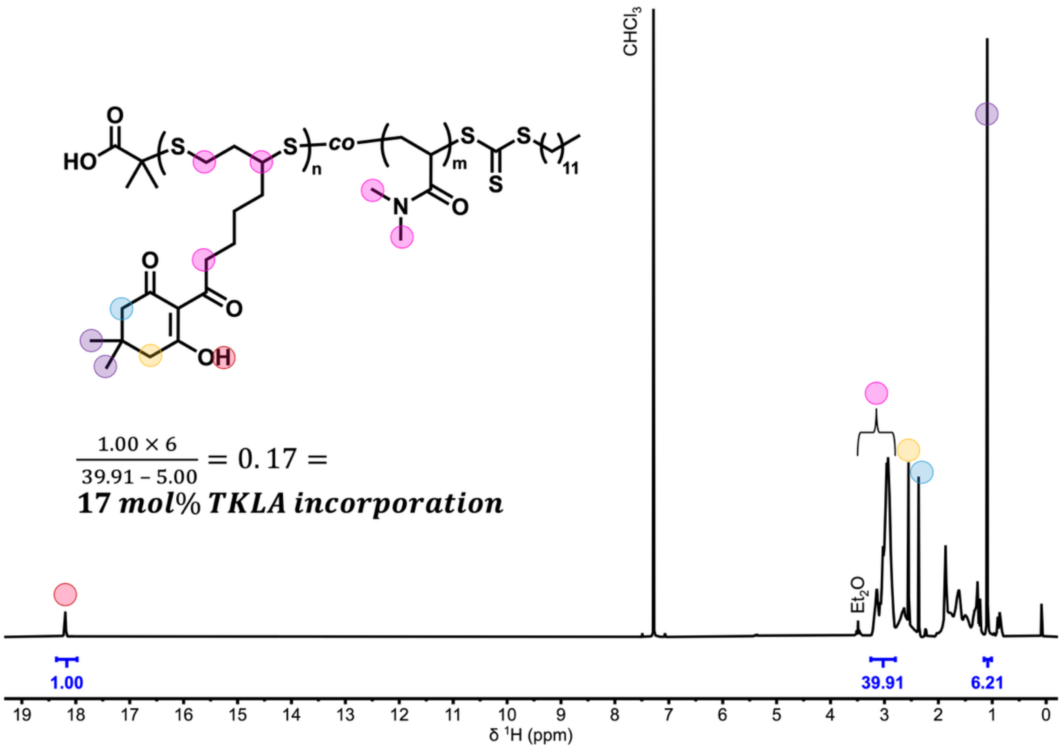

Figure S16. ^{1}H NMR of HMW P(TKLA-*co*-DMA), recorded in $CDCl_3$.

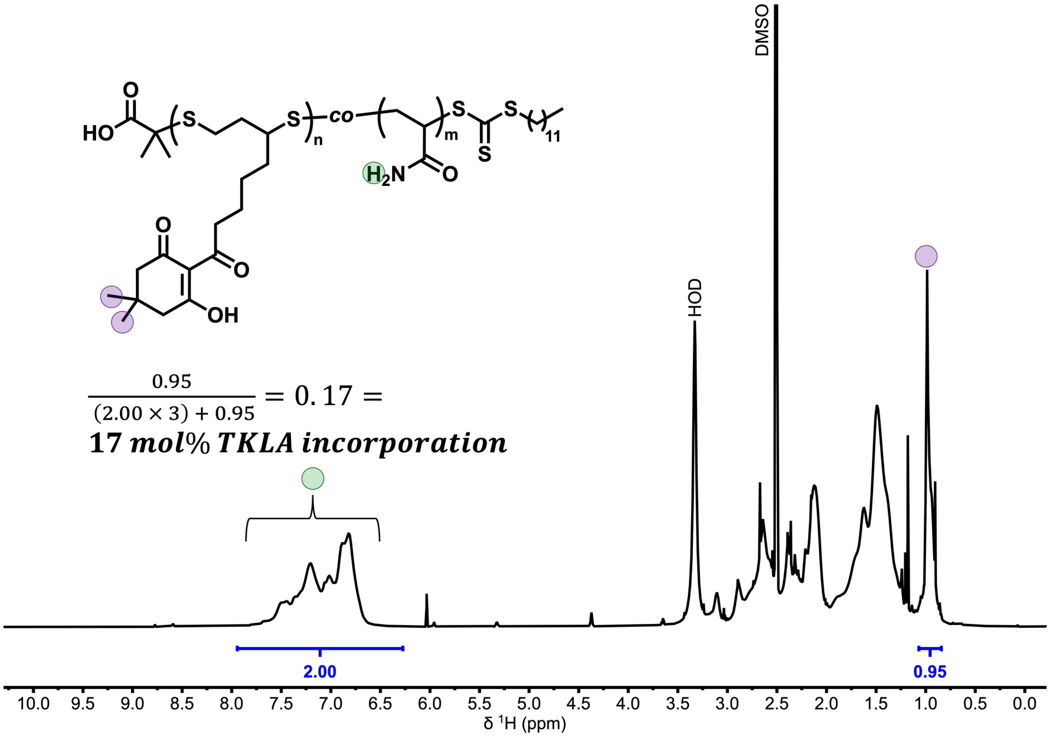

Figure S17. ^{1}H NMR of P(TKLA-*co*-Am), recorded in DMSO-*d*$_6$. The triketone enolic proton is not visible due to dynamic hydrogen bonding with the primary amide.

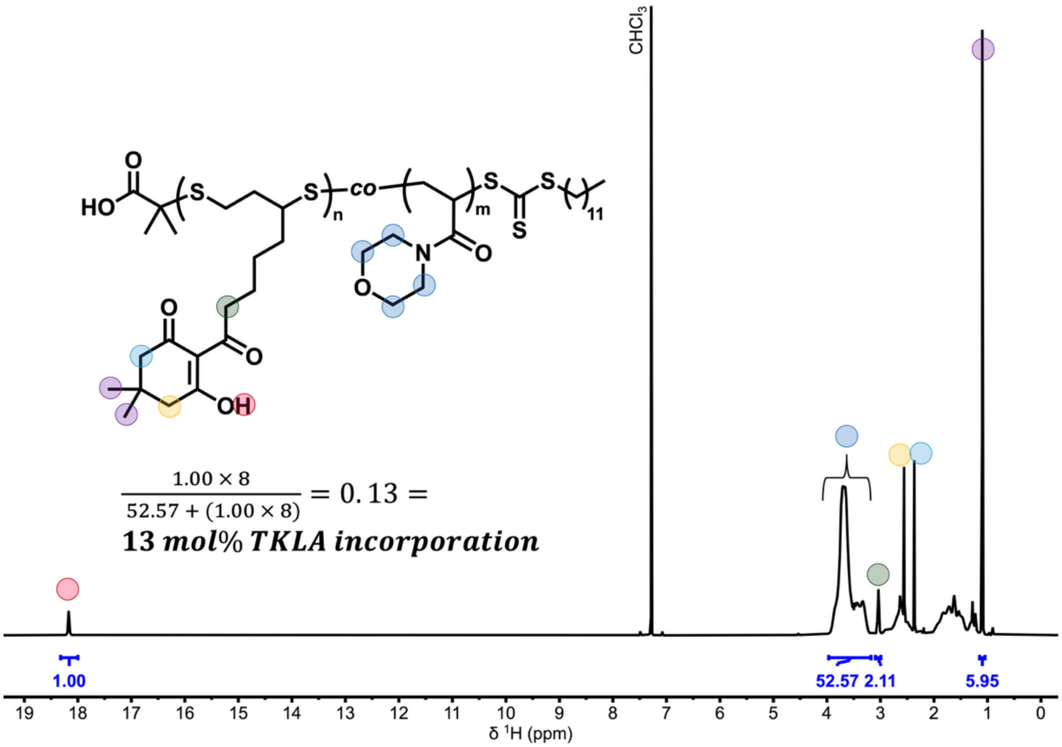

Figure S18. ^{1}H NMR of P(TKLA-*co*-NAM), recorded in $CDCl_3$.

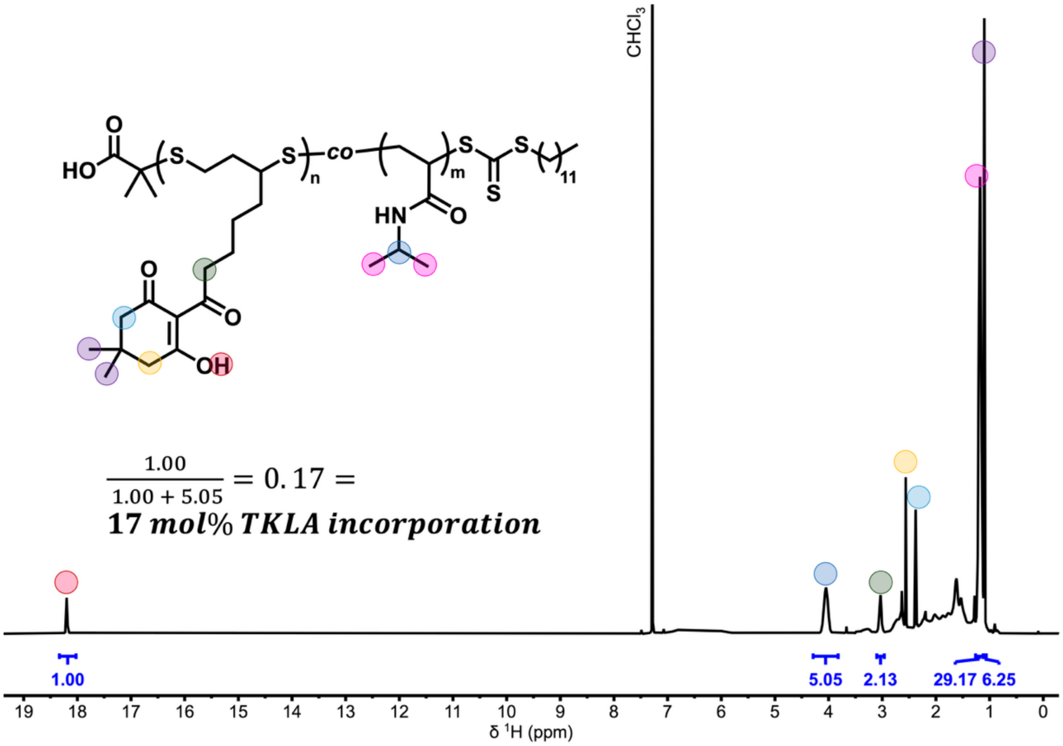

Figure S19. ^{1}H NMR of P(TKLA-*co*-NiPAM), recorded in $CDCl_3$.

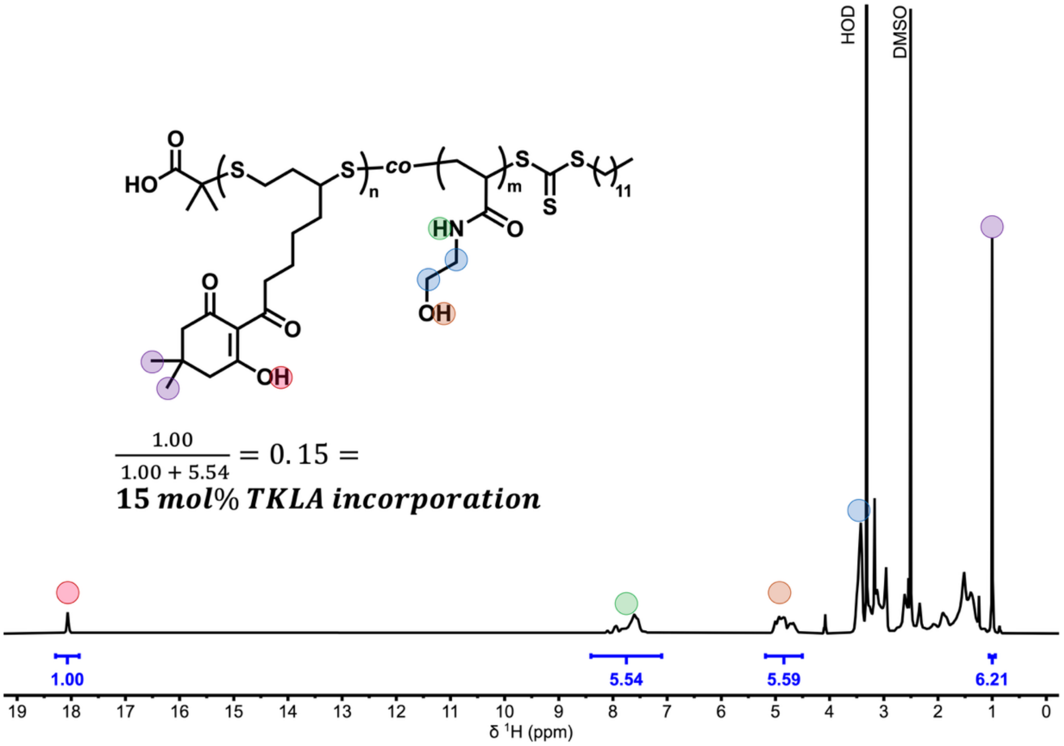

Figure S20. ^{1}H NMR of P(TKLA-*co*-HEAm), recorded in DMSO-d_6.

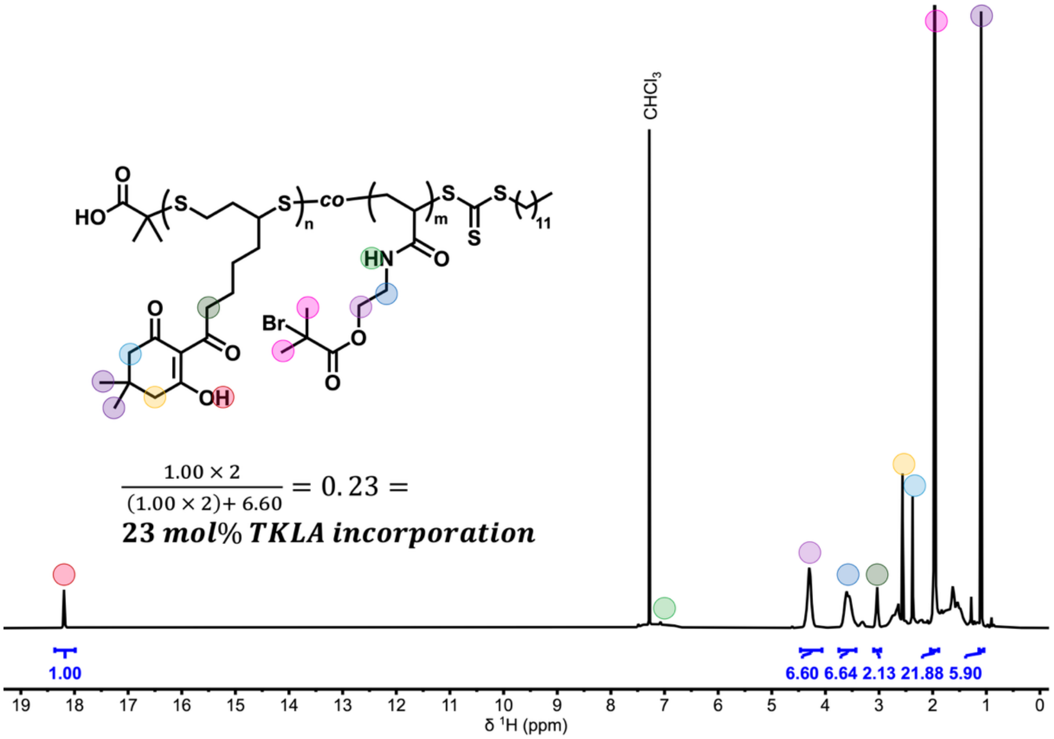

Figure S21. ^{1}H NMR of P(TKLA-*co*-BiBAm), recorded in $CDCl_3$.

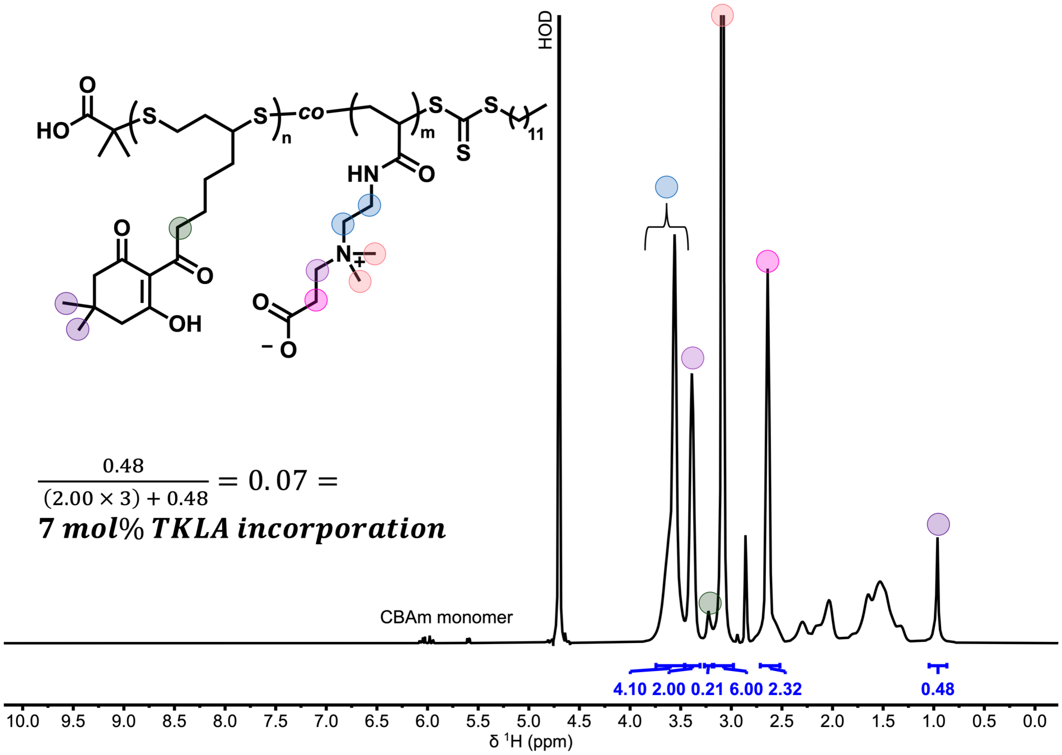

Figure S22. ^{1}H NMR of P(TKLA-*co*-CBAm), recorded in D_2O. The triketone enolic proton is not visible due to dynamic hydrogen bonding with the solvent.

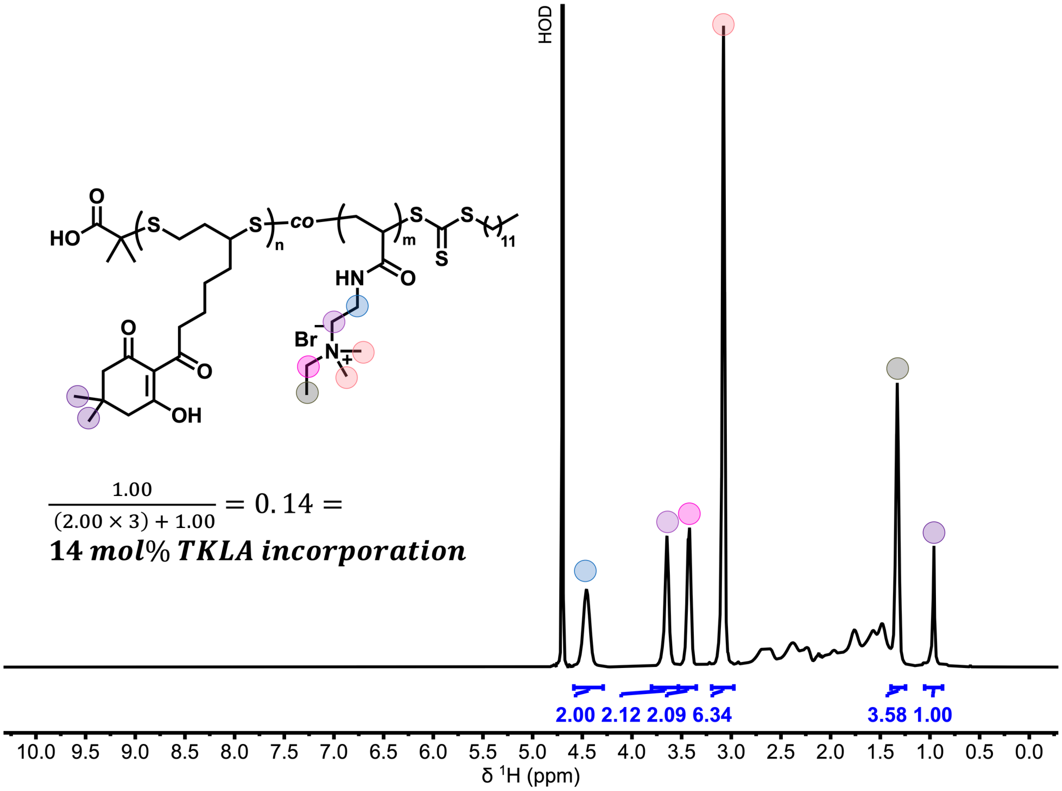

Figure S23. ^{1}H NMR of P(TKLA-*co*-DMEQAAm), recorded in D_2O. The triketone enolic proton is not visible due to dynamic hydrogen bonding with the solvent.

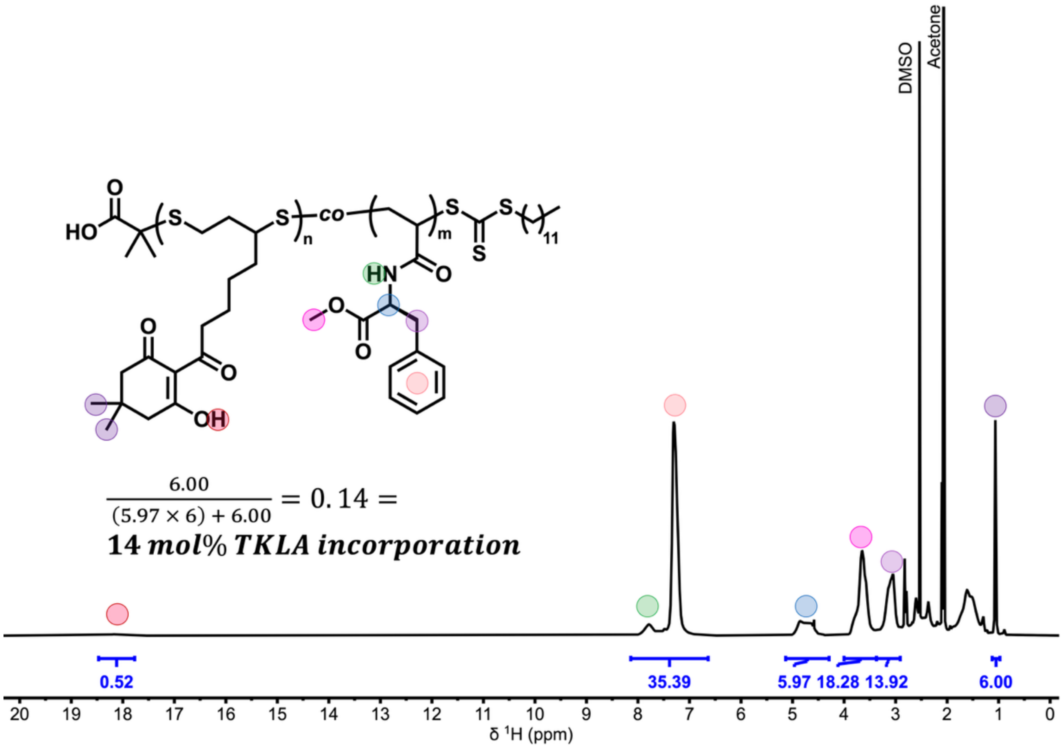

Figure S24. ^{1}H NMR of P(TKLA-*co*-Am-Phe-OMe), recorded in acetone-d_6. The peak corresponding to the TK (δ = 18.2) appears as a broad singlet with integration lower than the actual proton count, due to dynamic hydrogen bonding with the solvent.

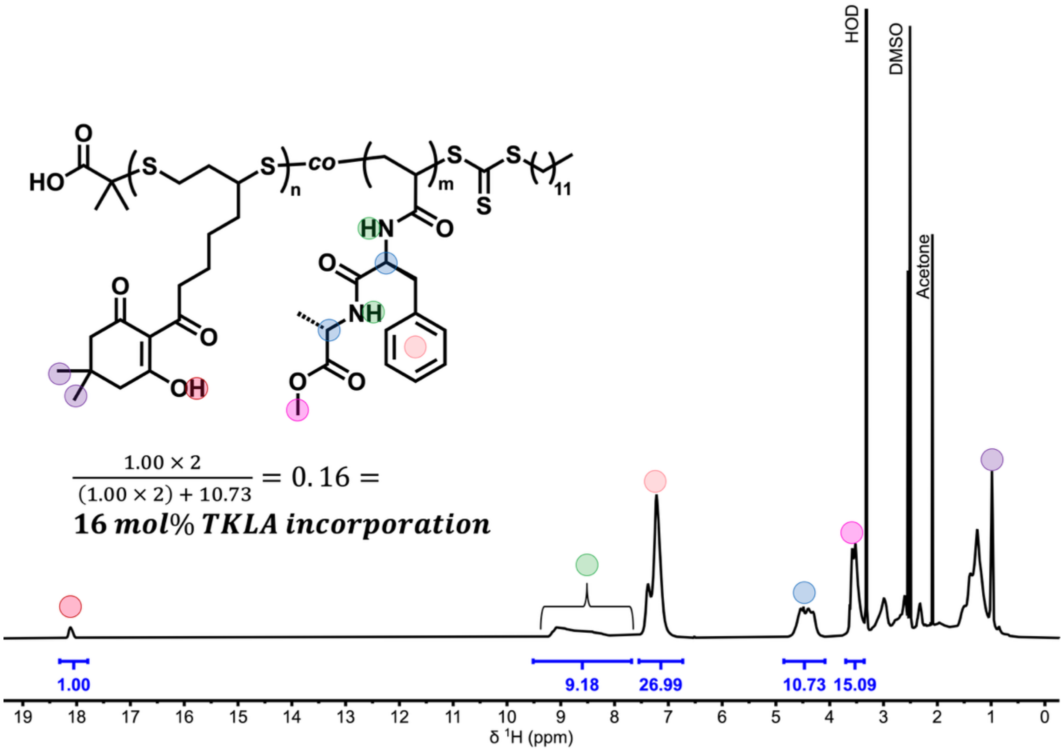

Figure S25. ^{1}H NMR of P(TKLA-*co*-Am-Phe-Ala-OMe), recorded in DMSO-d_6.

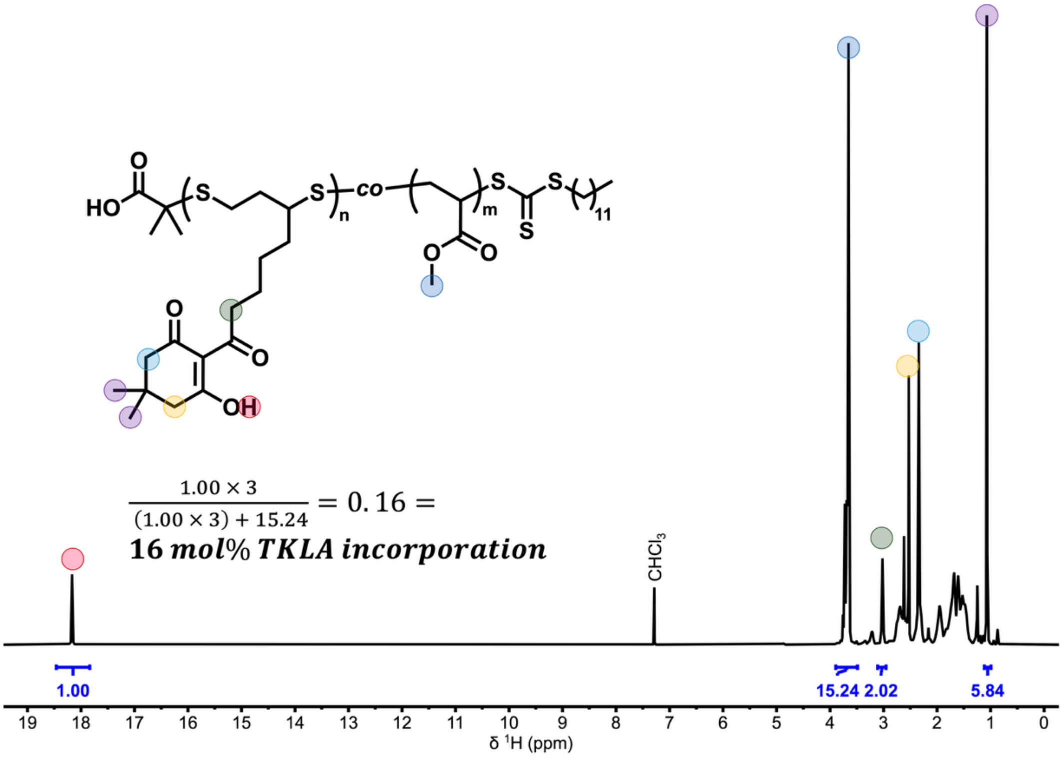

Figure S26. ^{1}H NMR of P(TKLA-*co*-MA), recorded in $CDCl_3$.

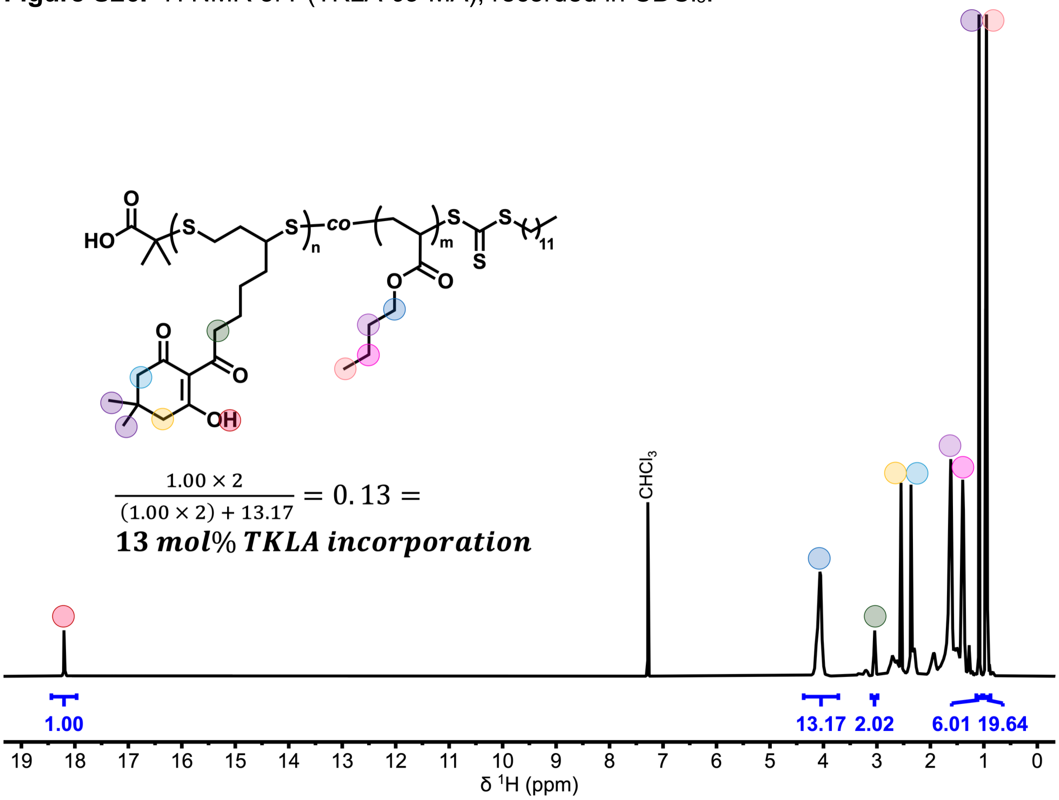

Figure S27. ^{1}H NMR of P(TKLA-*co*-BA), recorded in $CDCl_3$.

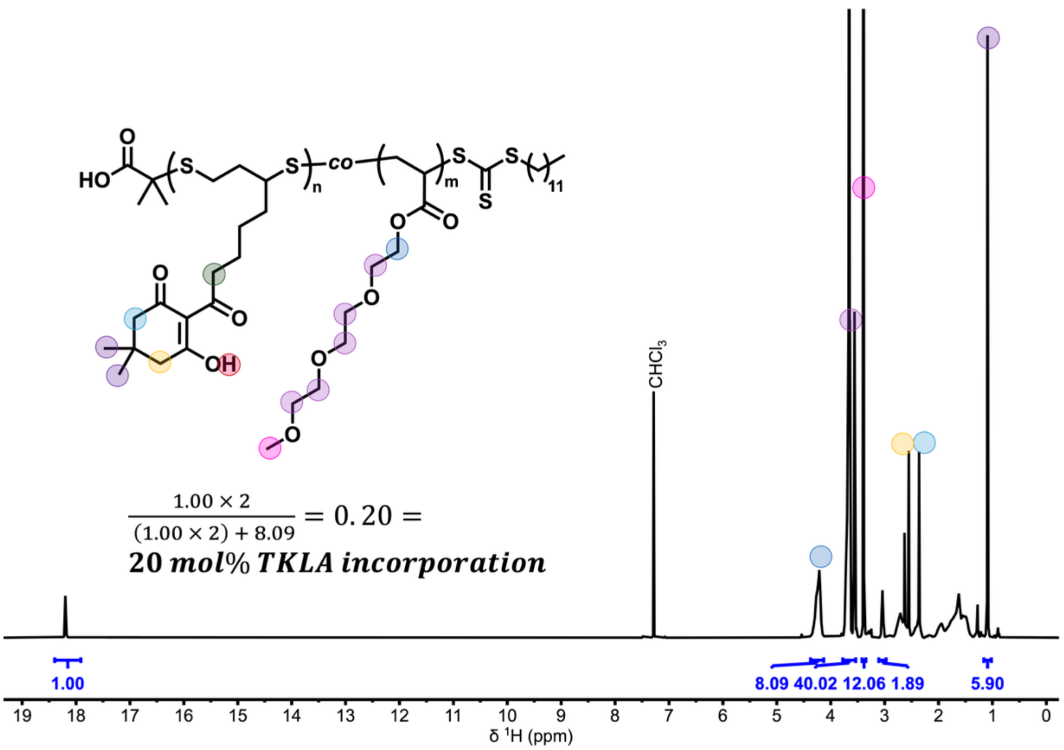

Figure S28. ^{1}H NMR of P(TKLA-*co*-TEGA), recorded in $CDCl_3$.

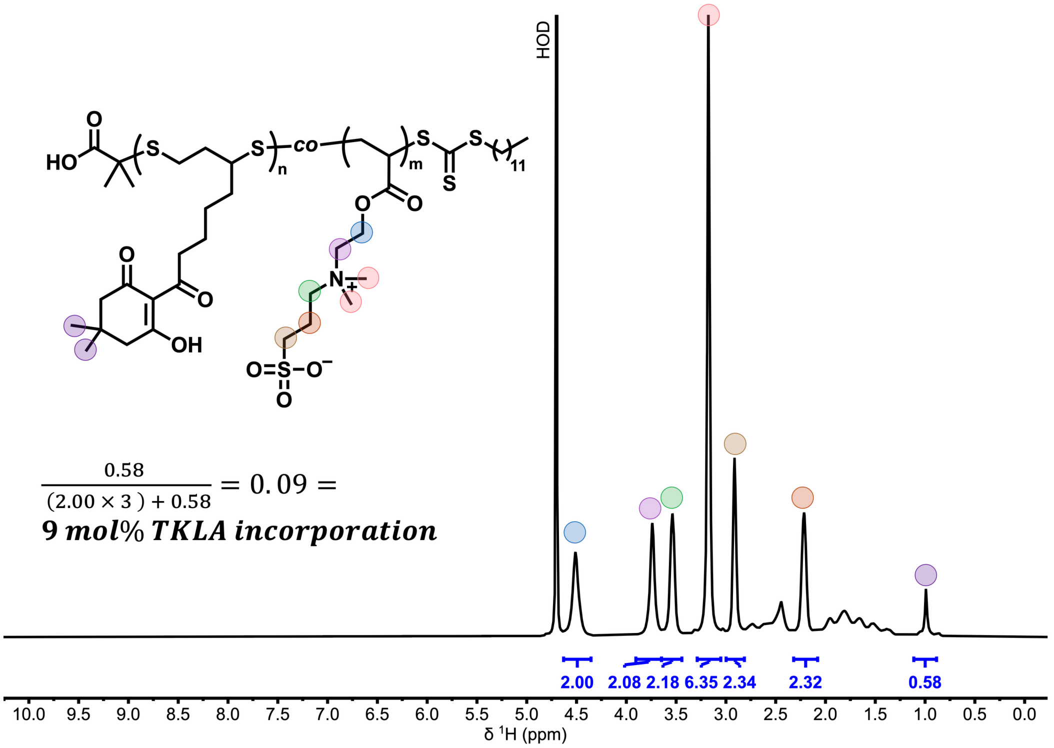

Figure S29. ^{1}H NMR of P(TKLA-*co*-SBA), recorded in D_2O. The triketone enolic proton is not visible due to dynamic hydrogen bonding with the solvent.

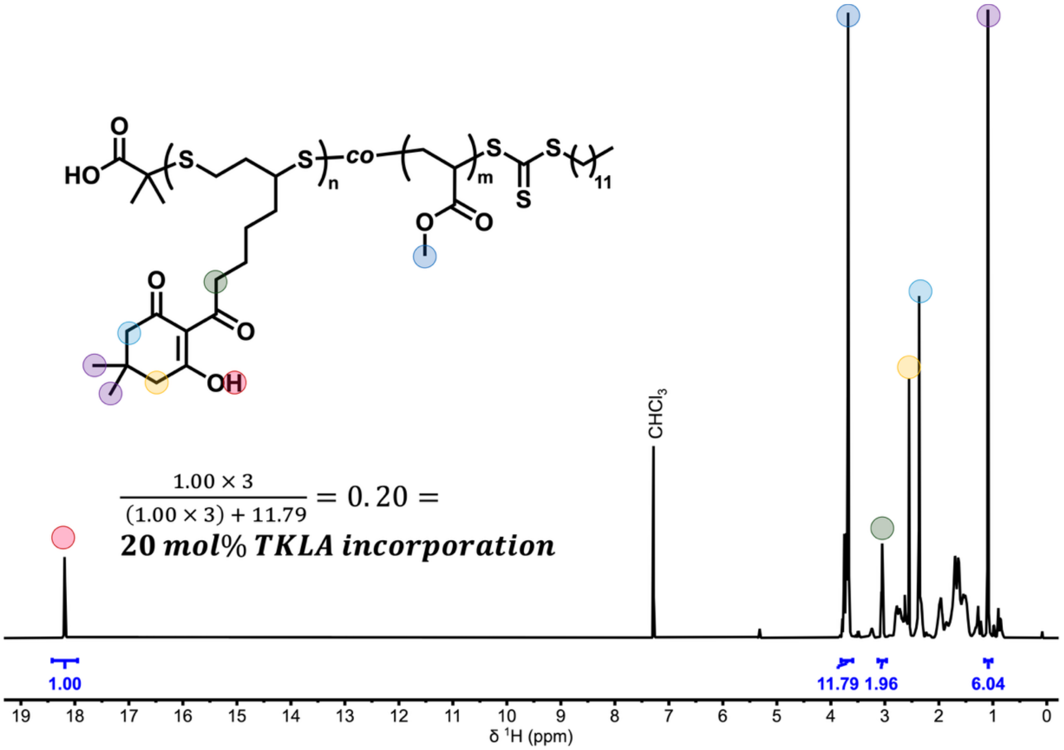

Figure S30. ^{1}H NMR of HMW P(TKLA-*co*-MA), recorded in $CDCl_3$.

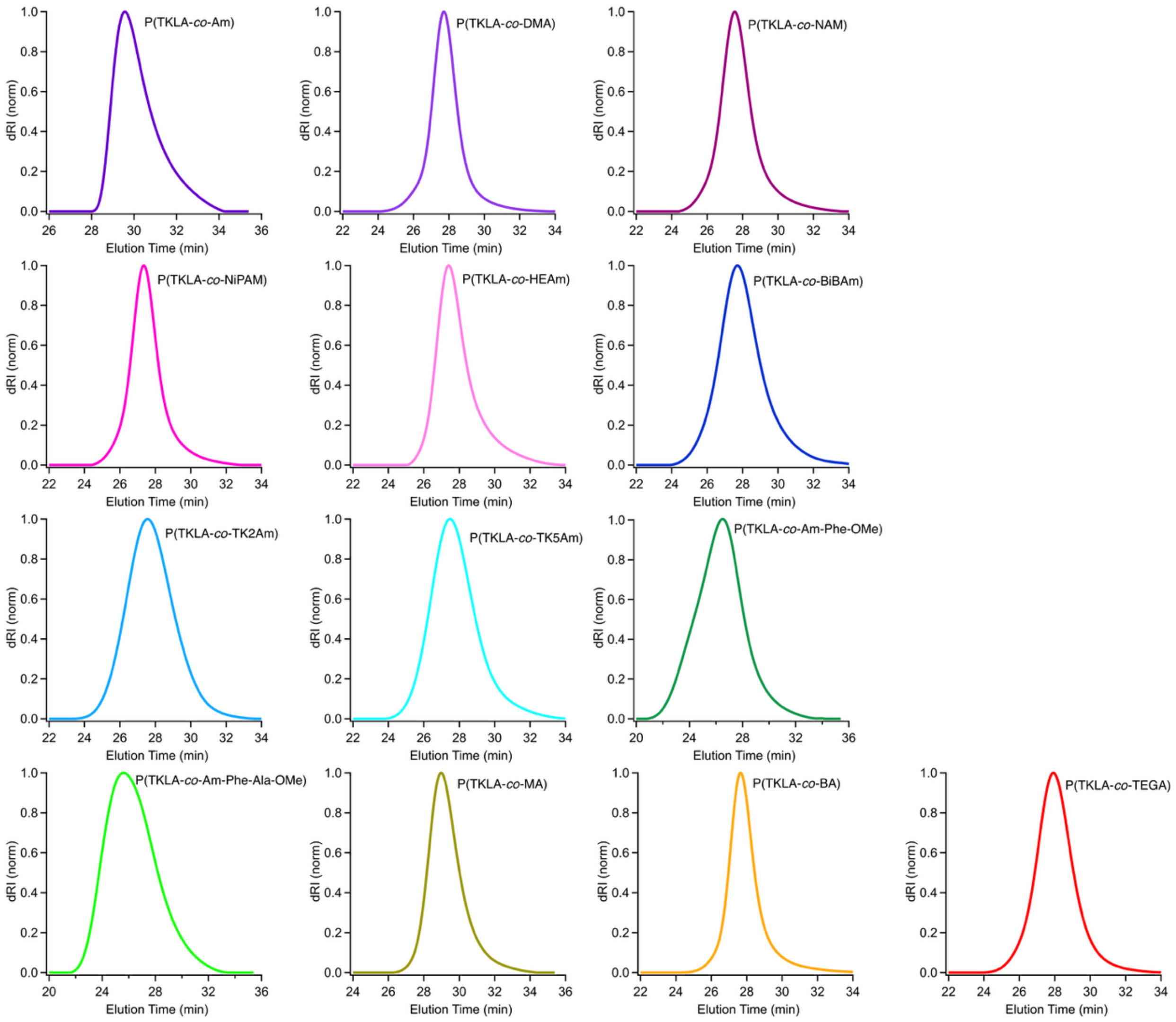

Figure S31. SEC traces corresponding to Table 3: Vinyl Comonomer Scope, run in SEC with DMF + 50 mM LiBr as eluent.

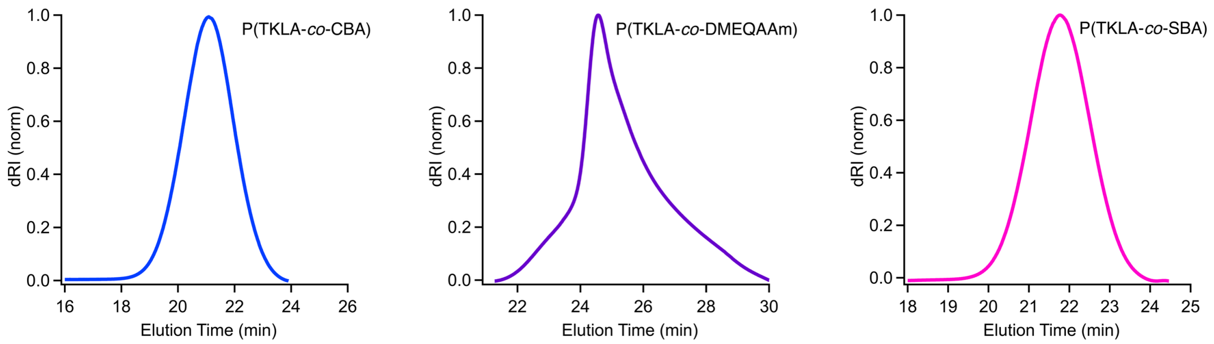

Figure S32. SEC traces corresponding to Table 3: Vinyl Comonomer Scope, run in aqueous buffer as eluent.

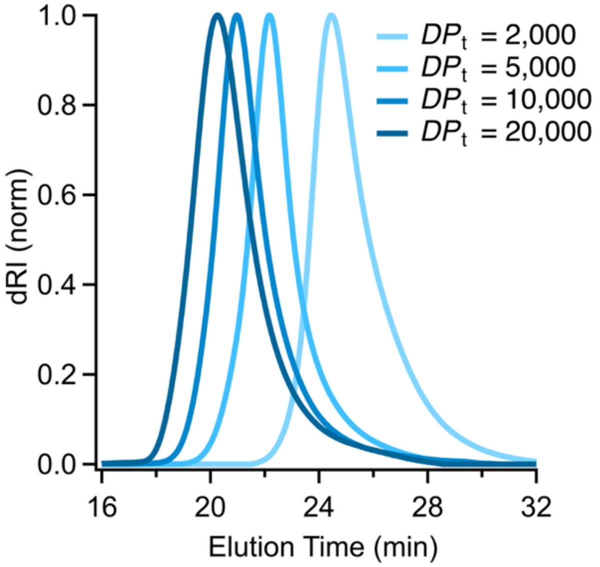

Figure S33. SEC traces corresponding Table S3: Copolymerization of TKLA with MA at high DP_t.

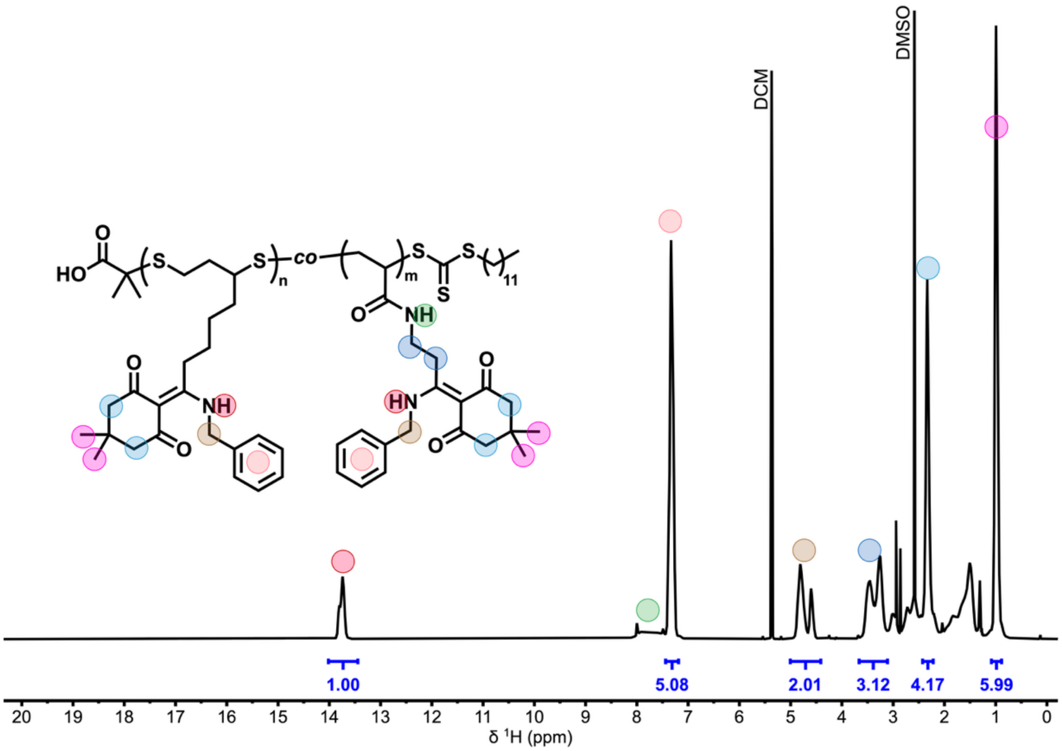

Figure S34. ¹H NMR of (DKE-benzyl) prepared from P(TKLA-*co*-TK2Am), recorded in DCM-d_2.

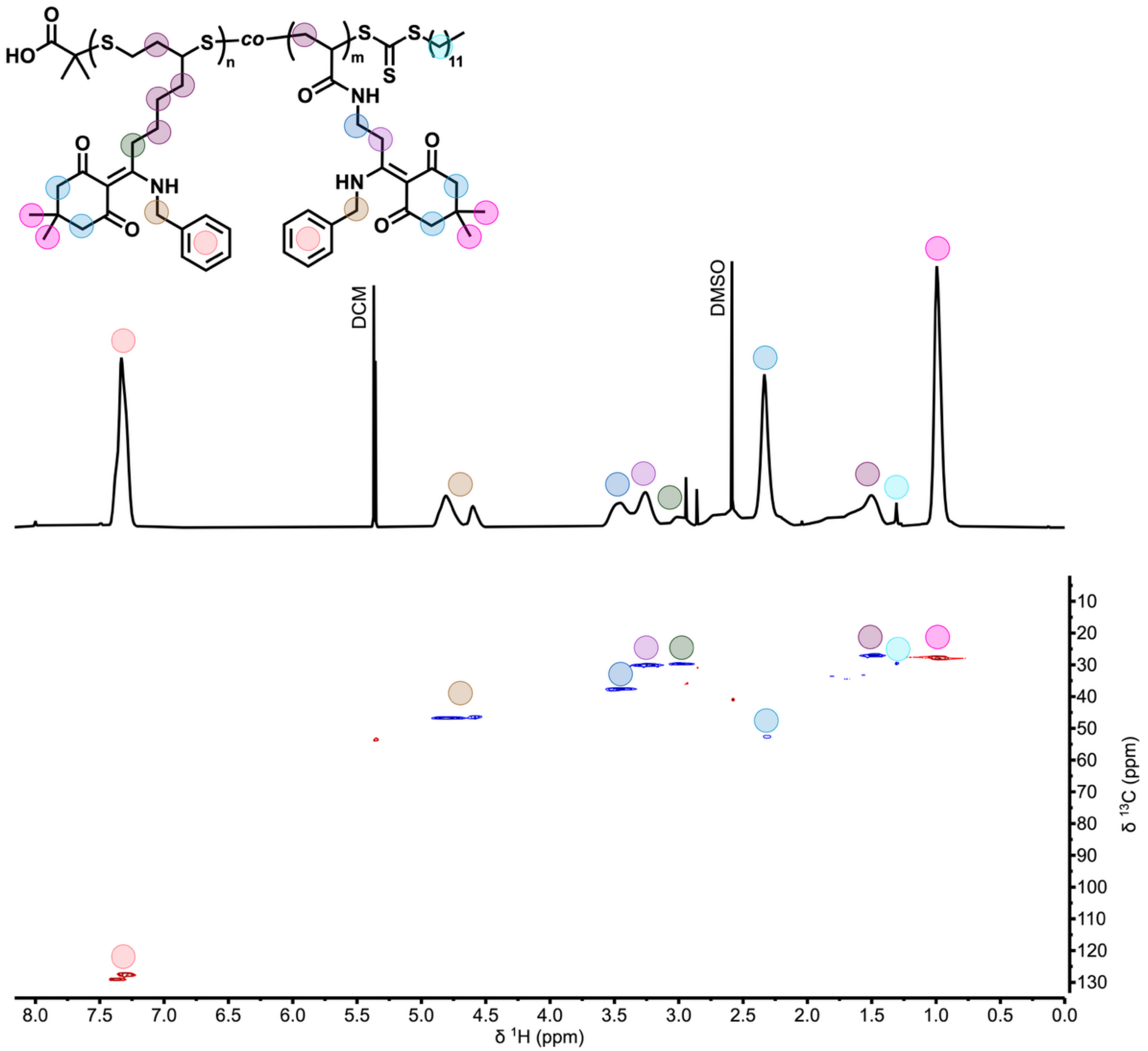

Figure S35. 2D ^{1}H-^{13}C HSQC NMR of (DKE-benzyl) prepared from P(TKLA-*co*-TK2Am), recorded in DCM-d_2.

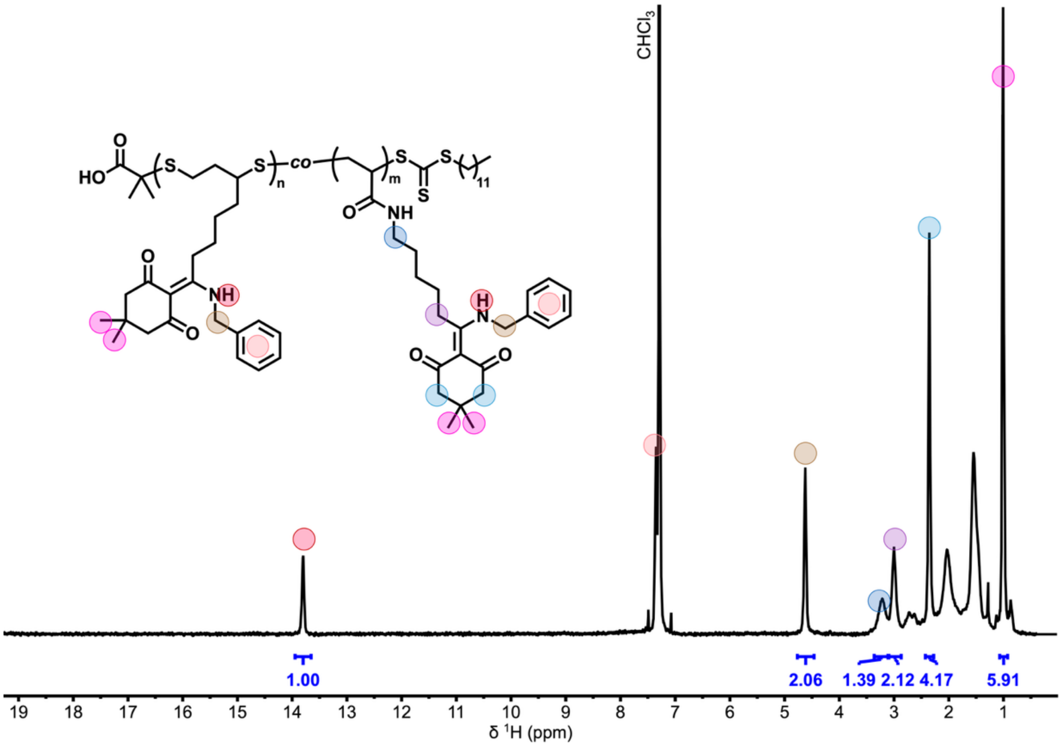

Figure S36. ^{1}H NMR of P(DKE-benzyl) prepared from P(TKLA-*co*-TK5Am), recorded in $CDCl_3$.

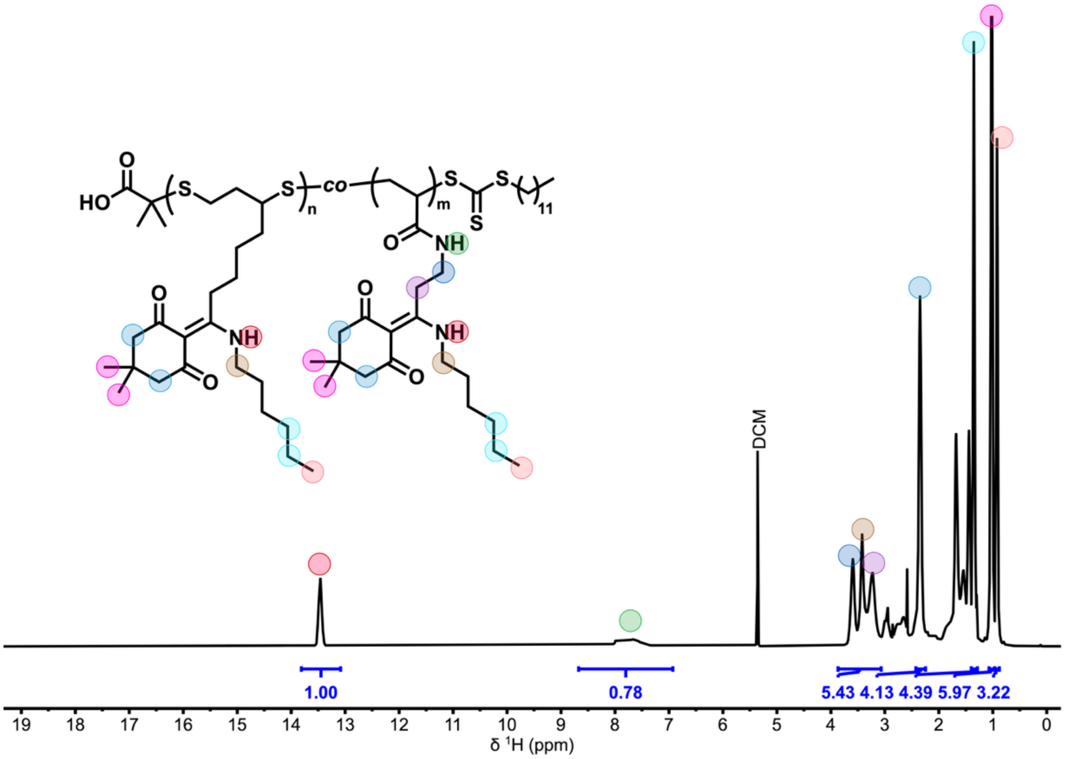

Figure S37. ^{1}H NMR of P(DKE-hexyl) prepared from P(TKLA-*co*-TK2Am), recorded in DCM-d_2.

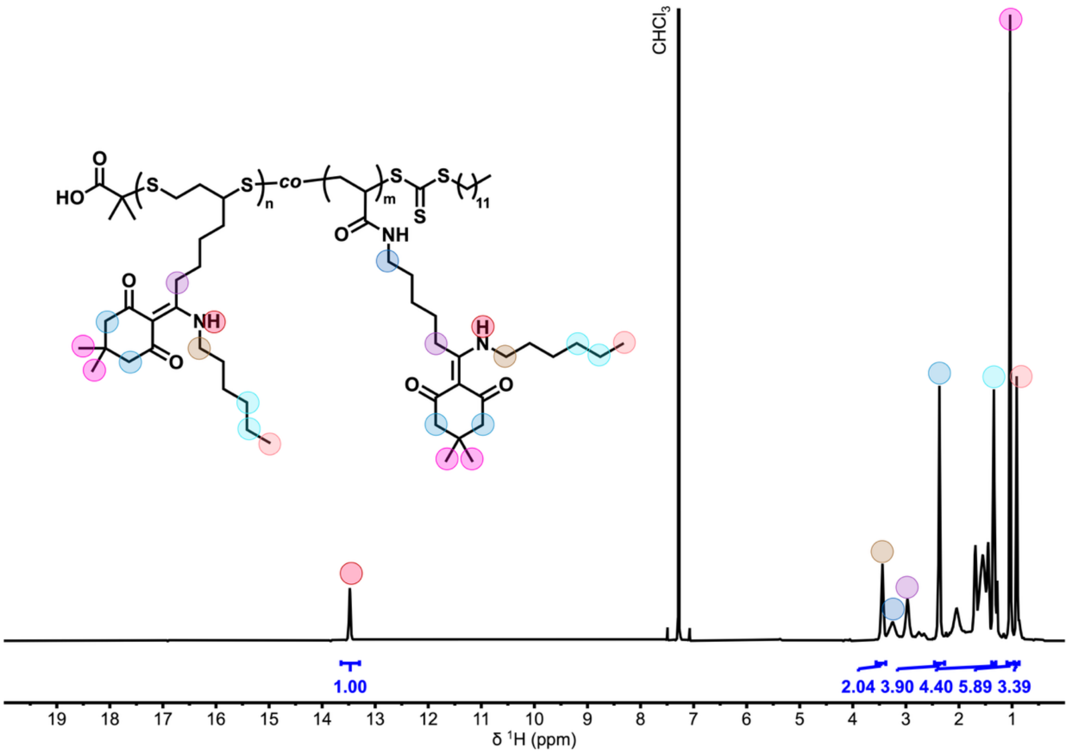

Figure S38. ^{1}H NMR of P(DKE-hexyl) prepared from P(TKLA-*co*-TK5Am), recorded in $CDCl_3$.

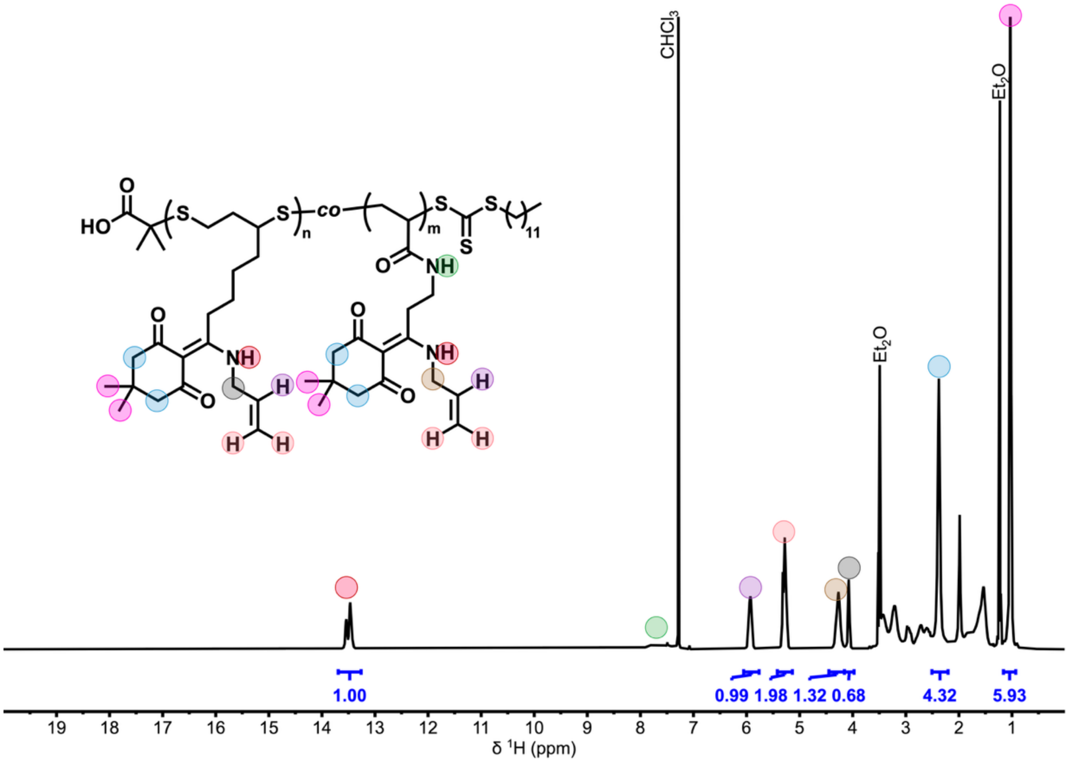

Figure S39. ^{1}H NMR of P(DKE-allyl) prepared from P(TKLA-*co*-TK2Am), recorded in $CDCl_3$.

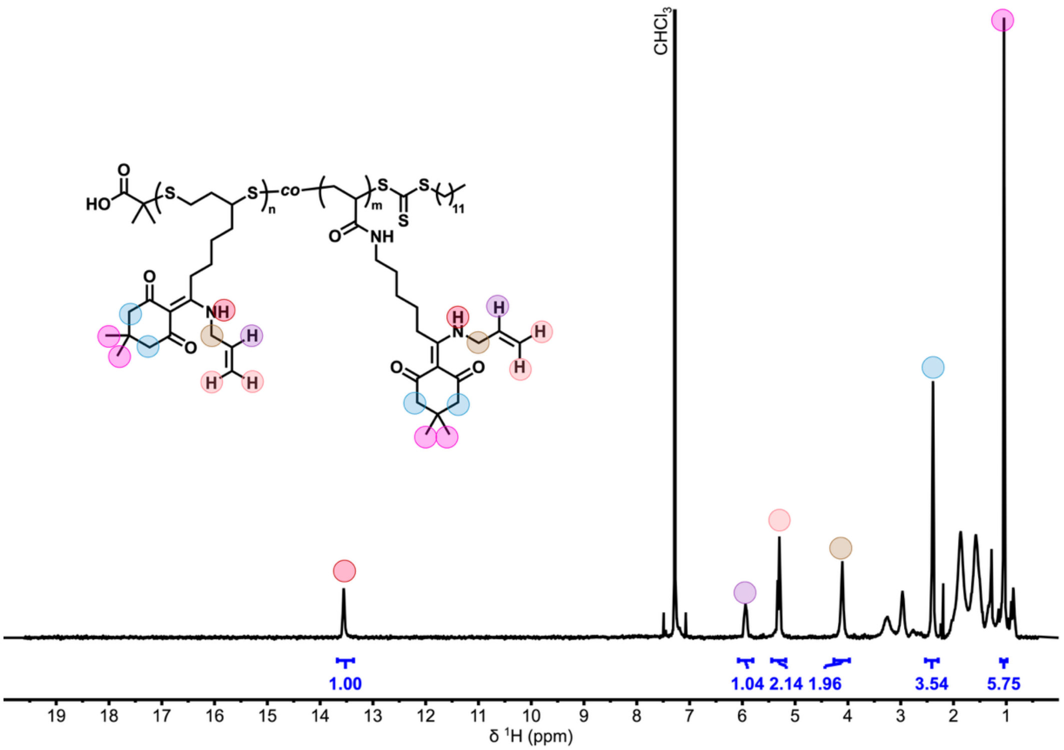

Figure S40. ^{1}H NMR of P(DKE-allyl) prepared from P(TKLA-*co*-TK5Am), recorded in $CDCl_3$.

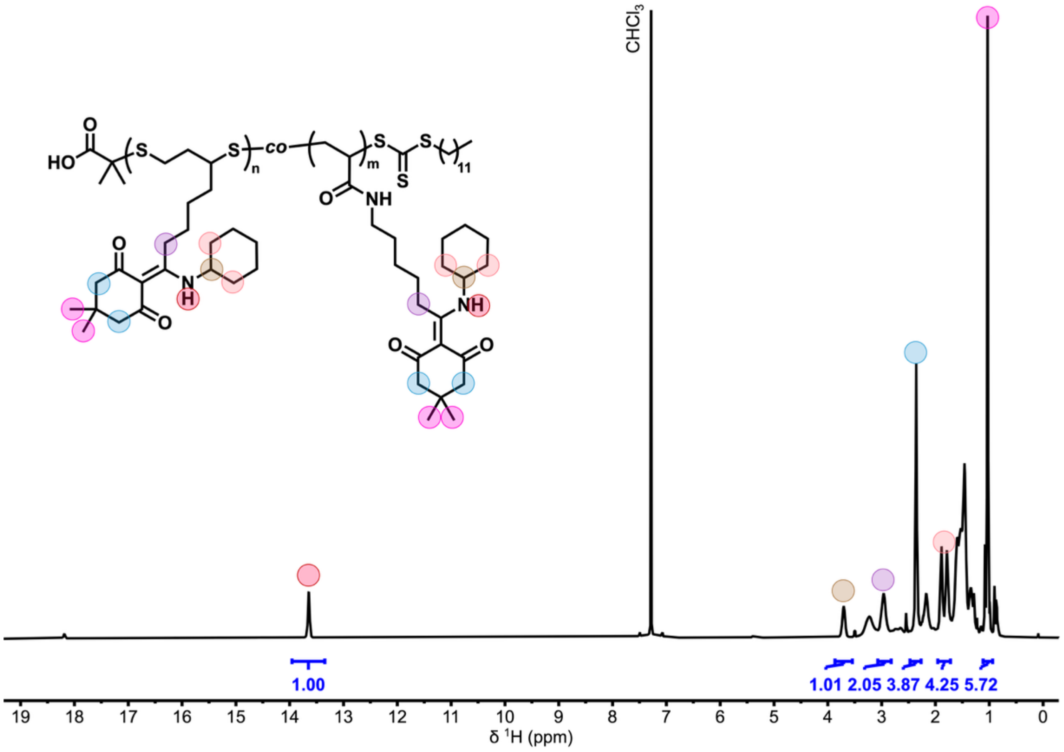

Figure S41. ^{1}H NMR of P(DKE-cyclohexyl) prepared from P(TKLA-*co*-TK5Am), recorded in $CDCl_3$.

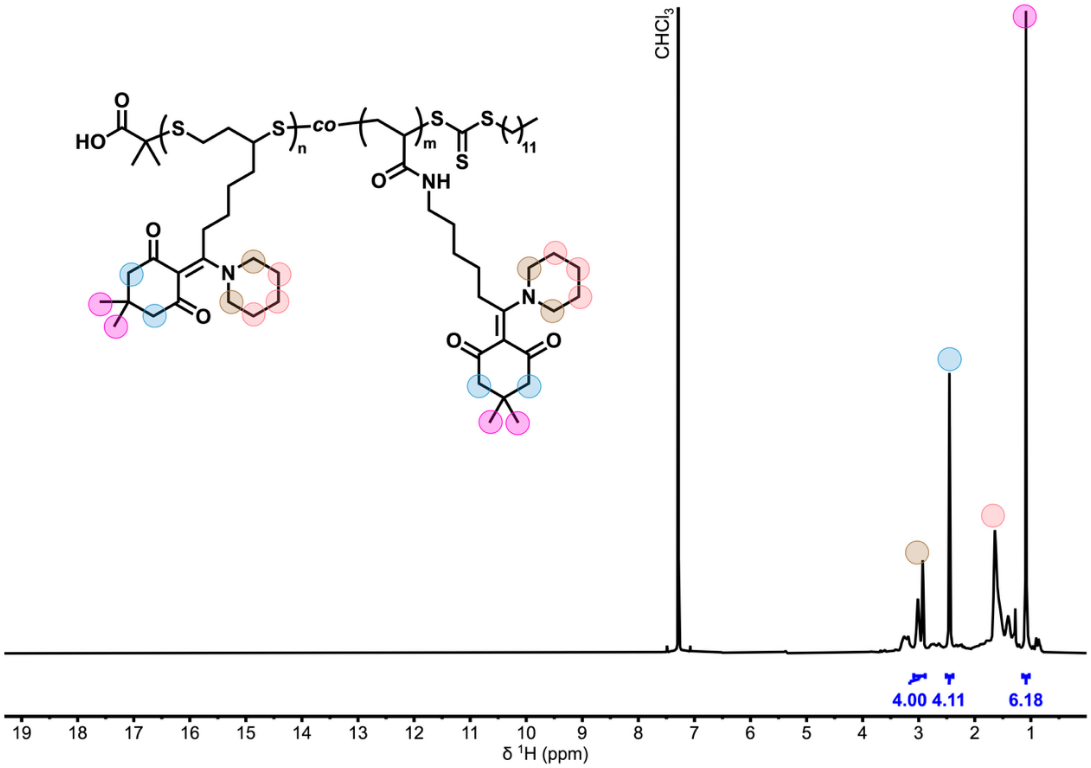

Figure S42. ^{1}H NMR of P(DKE-piperidine) prepared from P(TKLA-*co*-TK5Am), recorded in $CDCl_3$.

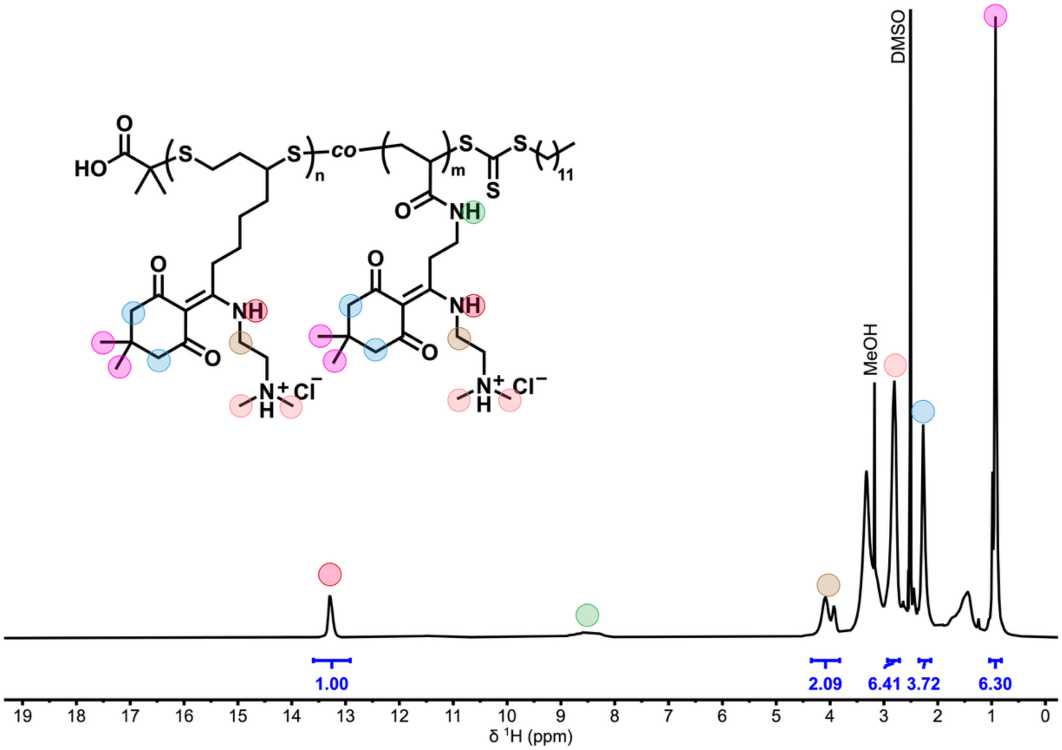

Figure S43. ^{1}H NMR of P(DKE-DMED-HCl) prepared from P(TKLA-*co*-TK5Am), recorded in DMSO-d_6.

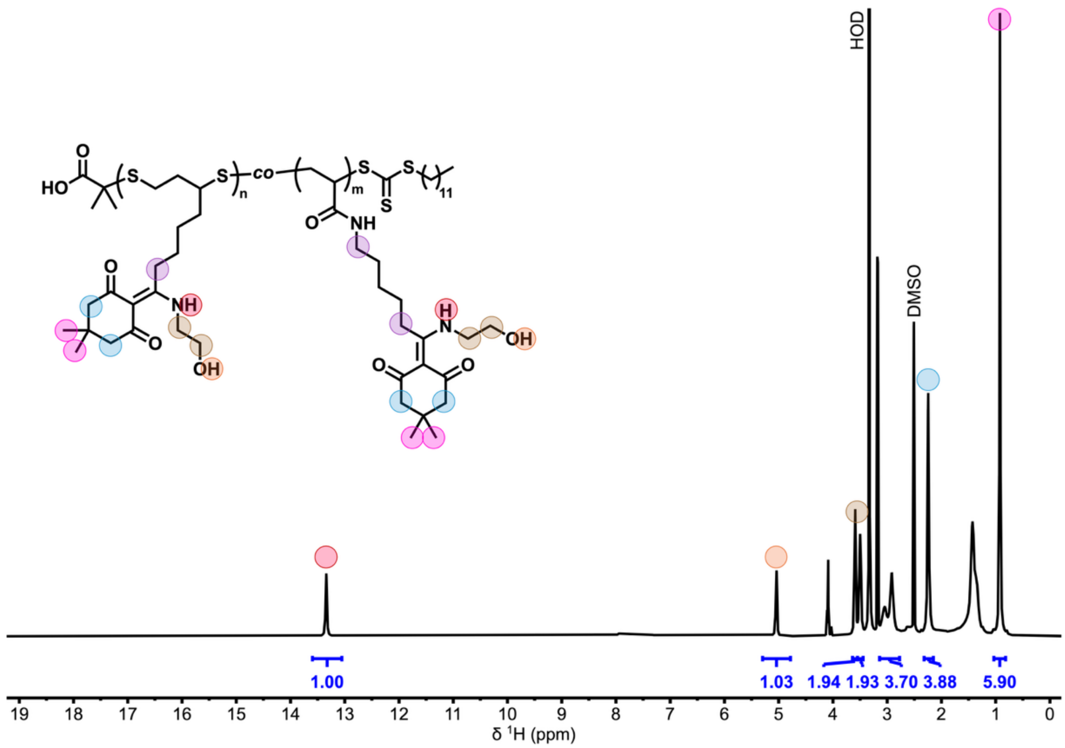

Figure S44. ^{1}H NMR of P(DKE-hydroxyethyl) prepared from P(TKLA-*co*-TK5Am), recorded in DMSO-d_6.

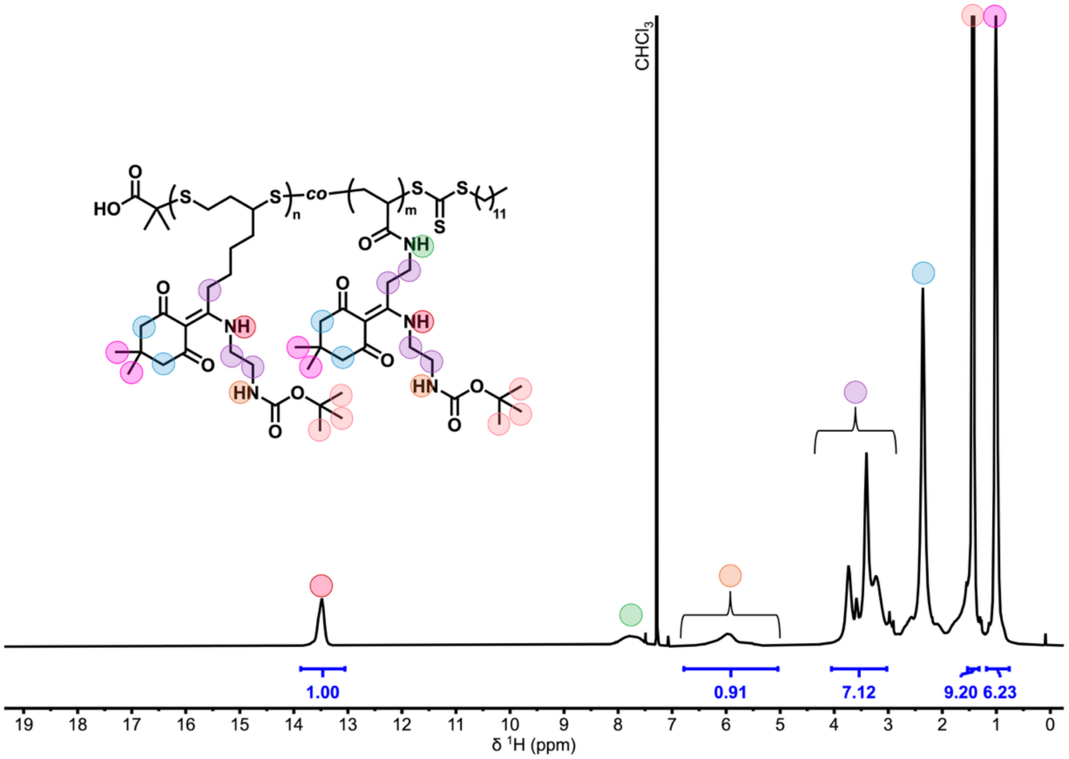

Figure S45. ^{1}H NMR of P(DKE-BOC-ED) prepared from P(TKLA-*co*-TK2Am), recorded in $CDCl_3$.

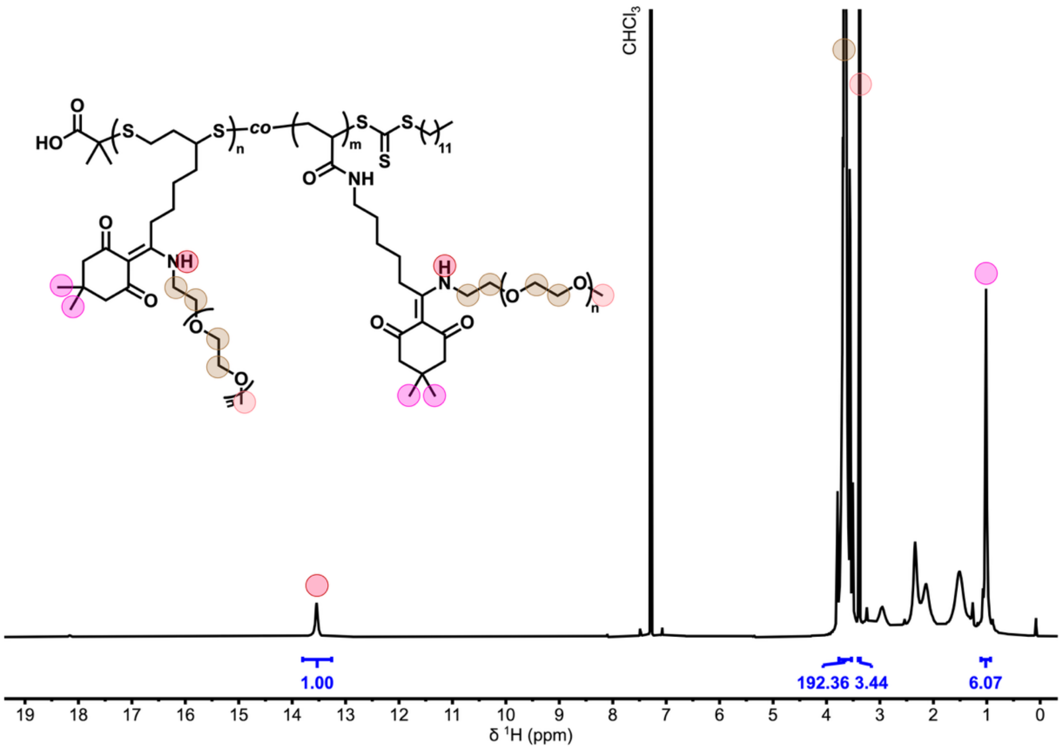

Figure S46. ^{1}H NMR of P(DKE-PEG) bottlebrush prepared from P(TKLA-*co*-TK5Am), recorded in $CDCl_3$.

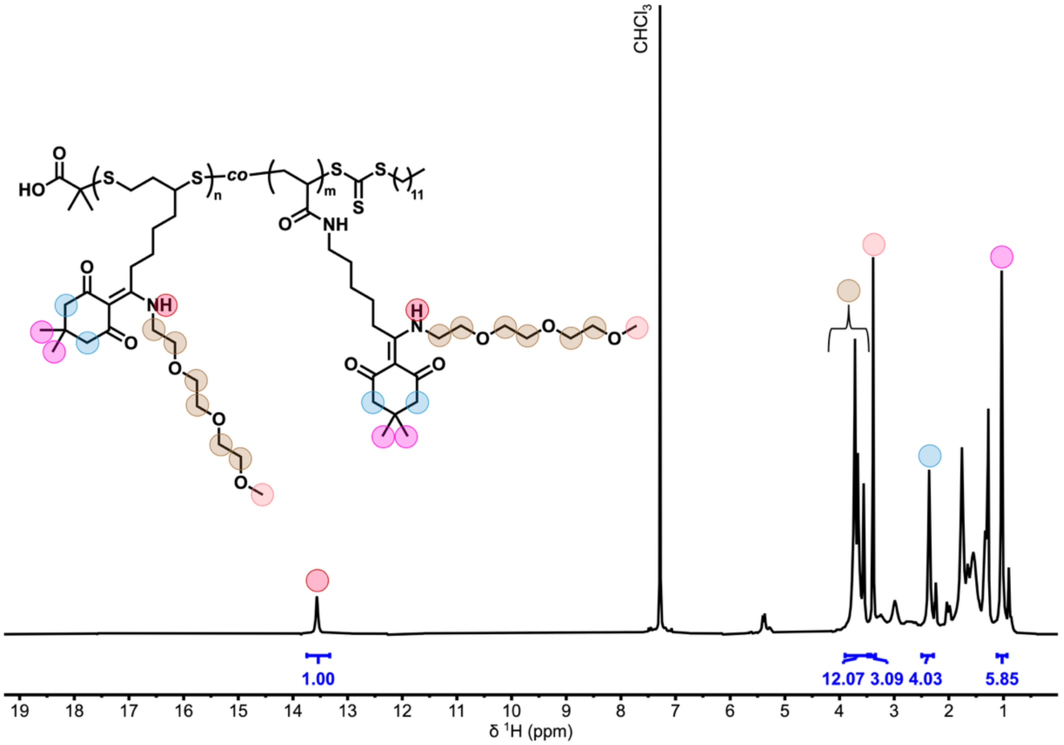

Figure S47. ^{1}H NMR of P(DKE-PEG3) prepared from P(TKLA-*co*-TK5Am), recorded in $CDCl_3$.

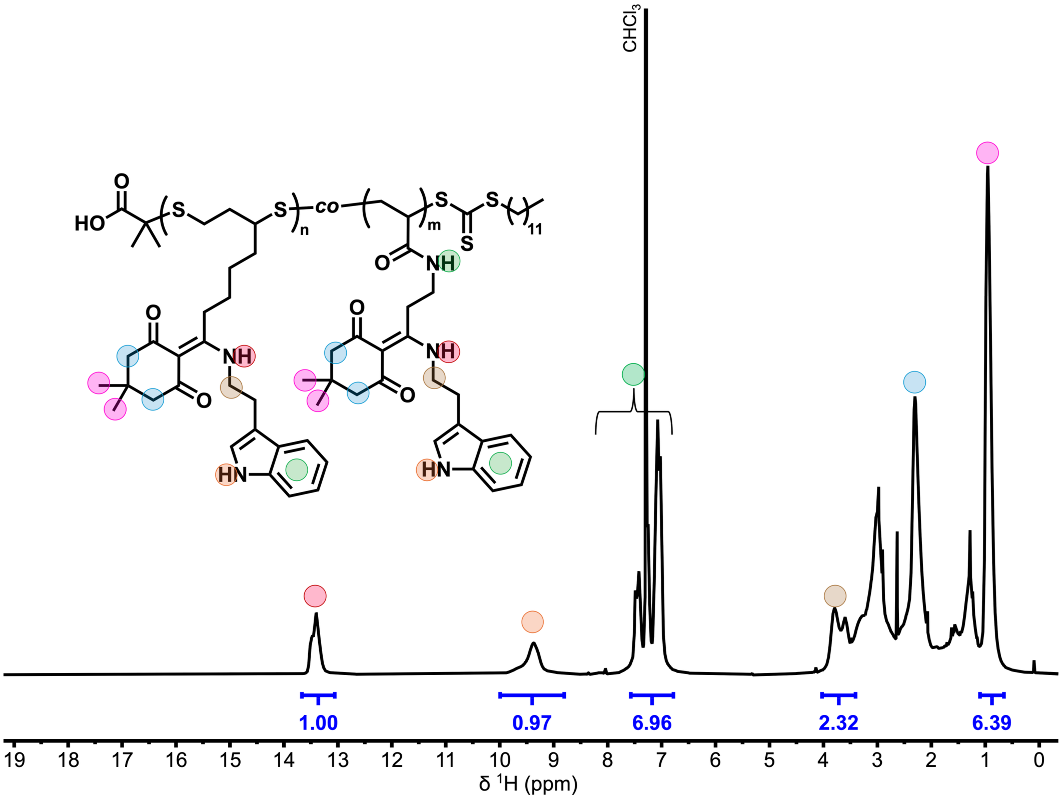

Figure S48. ^{1}H NMR of P(DKE-tryptamine) prepared from P(TKLA-*co*-TK2Am), recorded in $CDCl_3$.

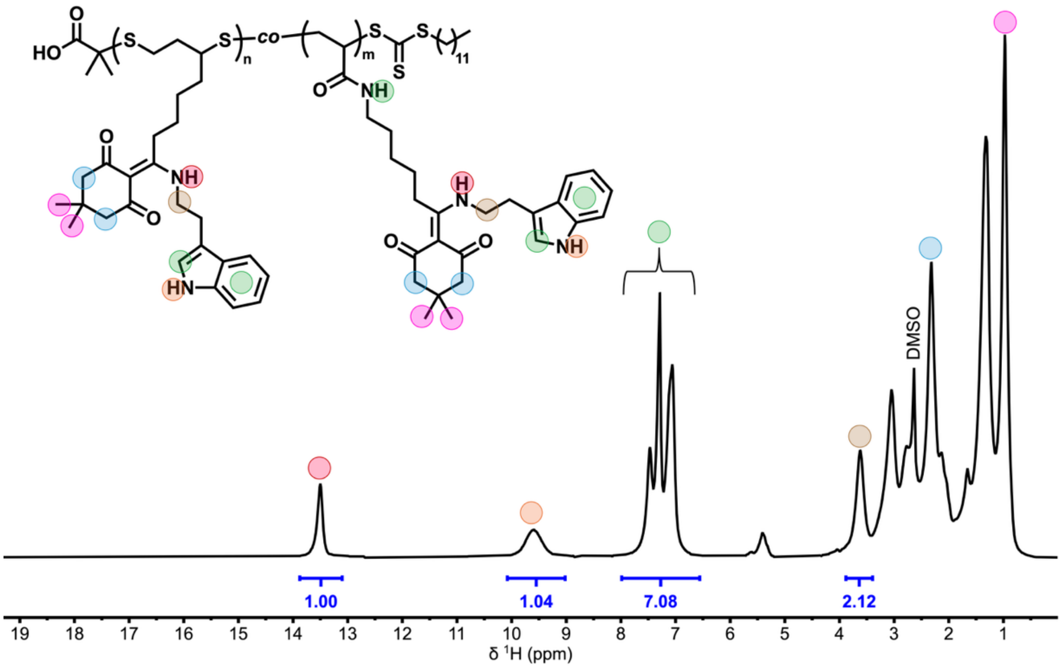

Figure S49. ^{1}H NMR of P(DKE-tryptamine) prepared from P(TKLA-*co*-TK5Am), recorded in DMSO-*d*$_6$.

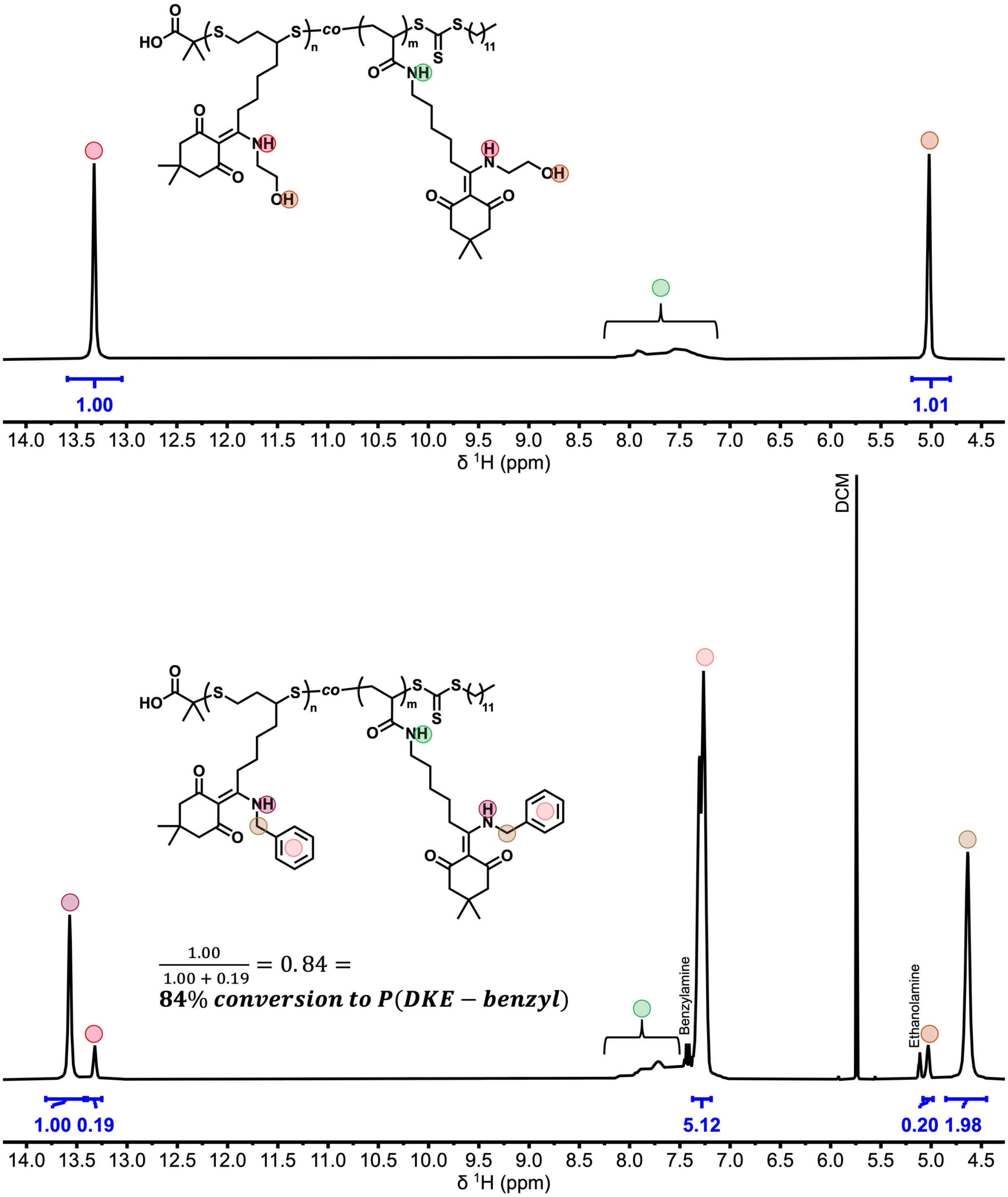

Figure S50. ^{1}H NMR overlay of P(DKE-hydroxyethyl) prepared from P(TKLA-*co*-TK5Am), and P(DKE-benzyl after transamination of P(DKE-hydroxyethyl) with benzylamine in DMF at 50 °C for 18 h.

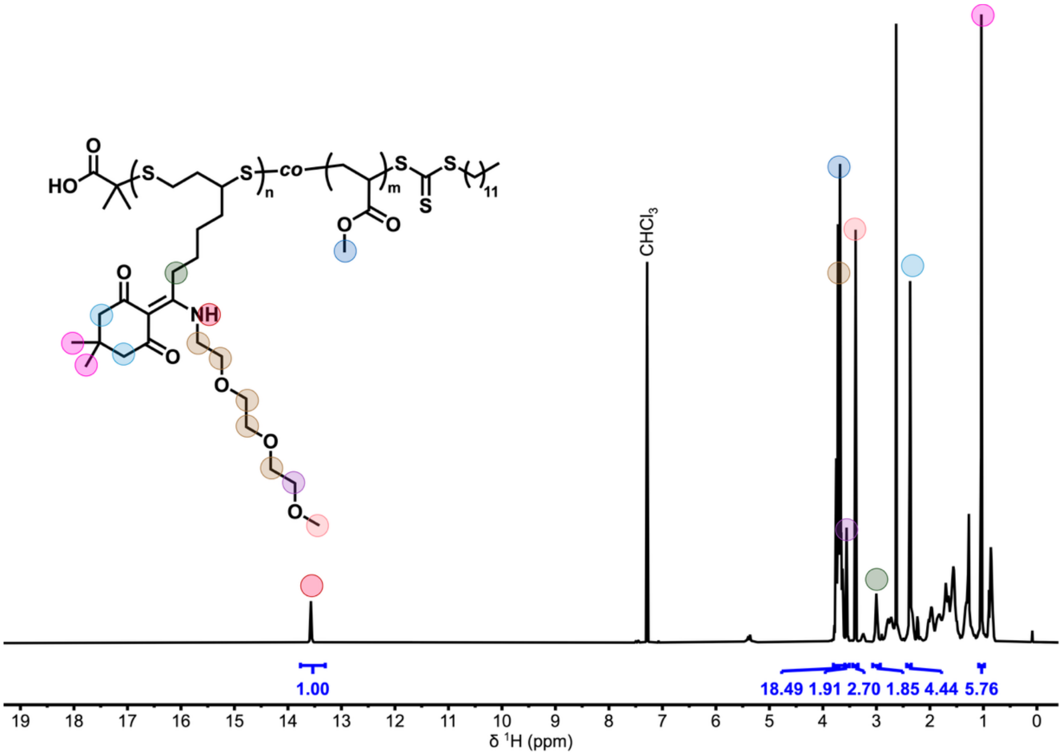

Figure S51. ^{1}H NMR of P(DKE-PEG3) prepared from HMW P(TKLA-*co*-MA), recorded in $CDCl_3$.

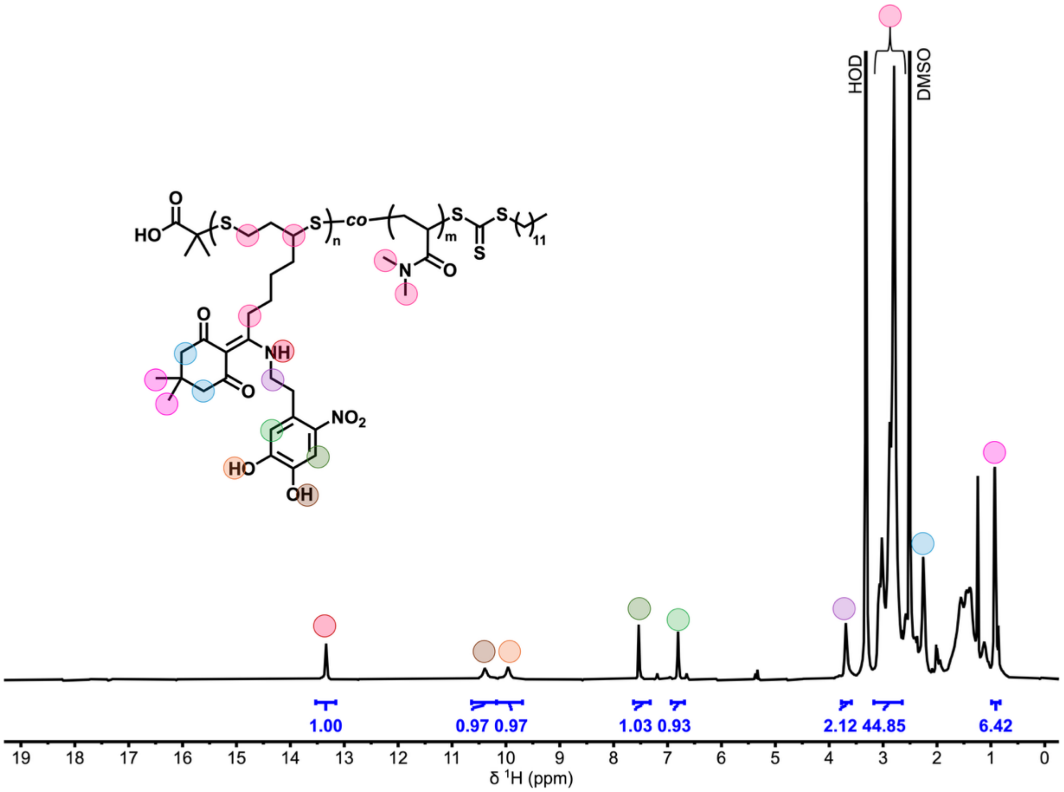

Figure S52. ^{1}H NMR of P(DKE-nitrodopamine) prepared from HMW P(TKLA-*co*-DMA), recorded in DMSO-*d*$_6$.

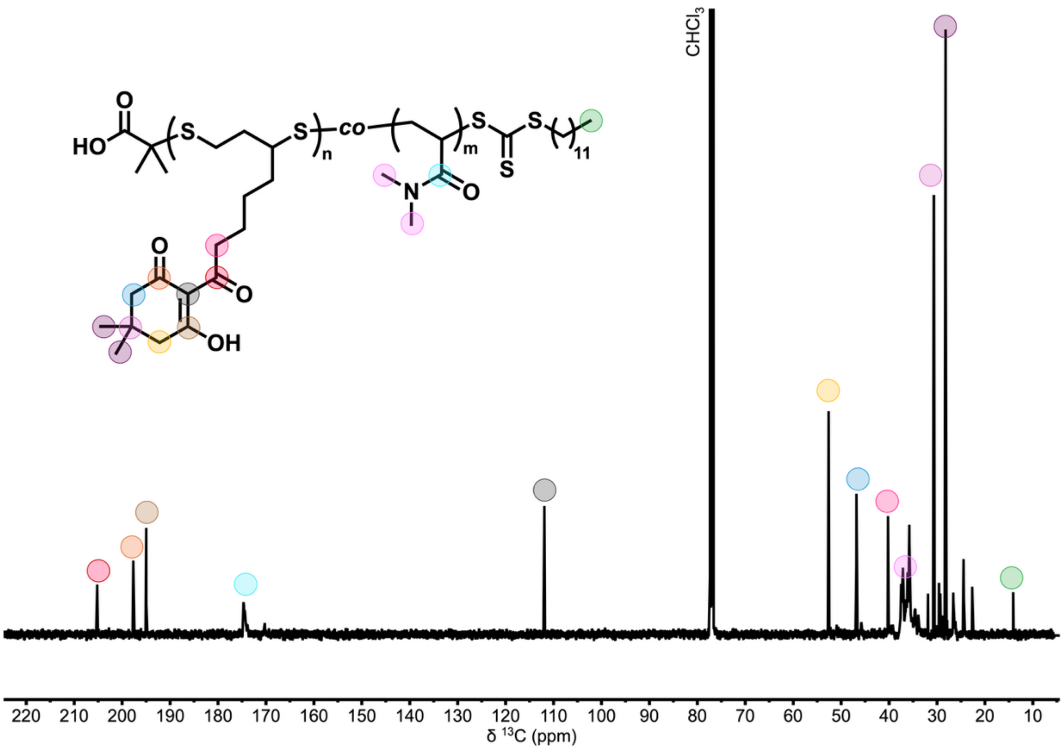

Figure S53. ^{13}C NMR of P(TKLA-*co*-DMA) macroinitiator used in chain extensions, recorded in $CDCl_3$.

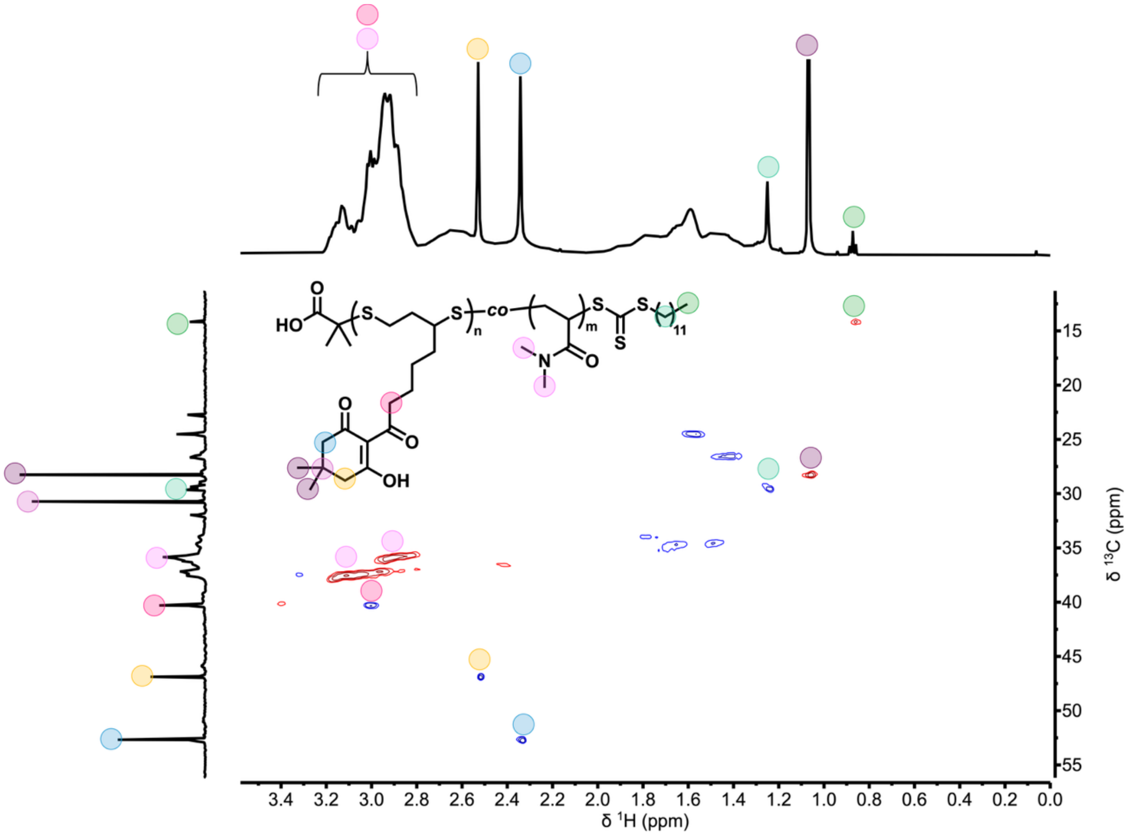

Figure S54. 2D ^{1}H-^{13}C HSQC NMR of P(TKLA-*co*-DMA) macroinitiator used in chain extensions, recorded in $CDCl_3$.

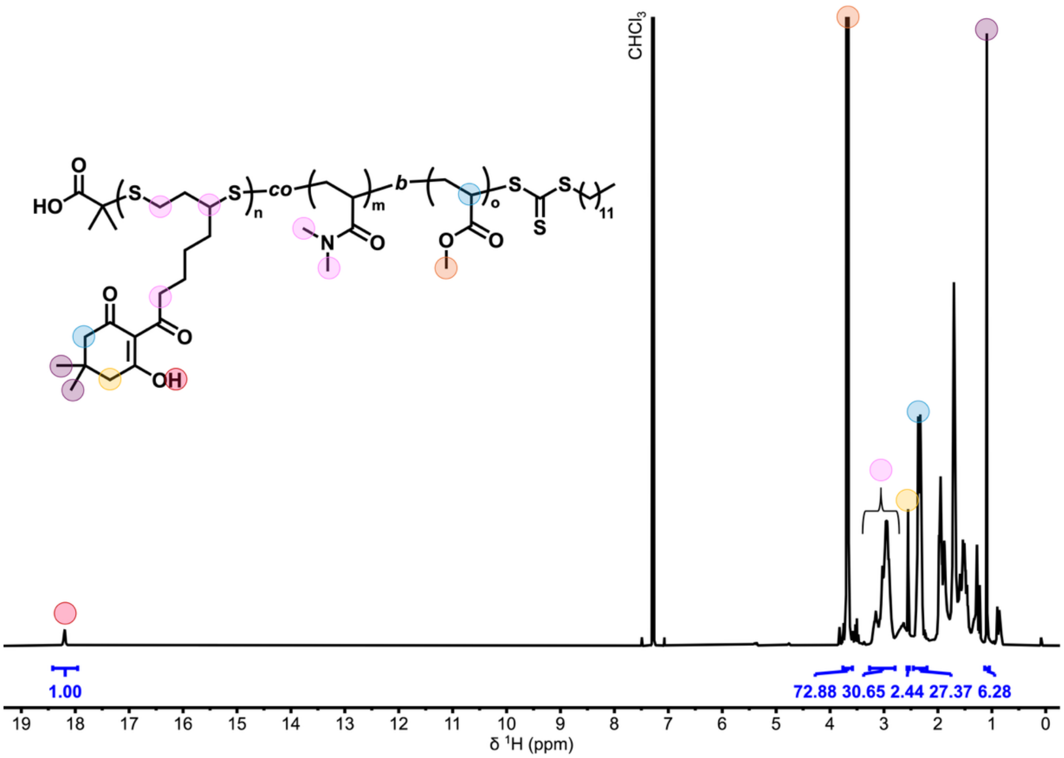

Figure S55. ^{1}H NMR of P(TKLA-*co*-DMA)-*b*-PMA, recorded in $CDCl_3$.

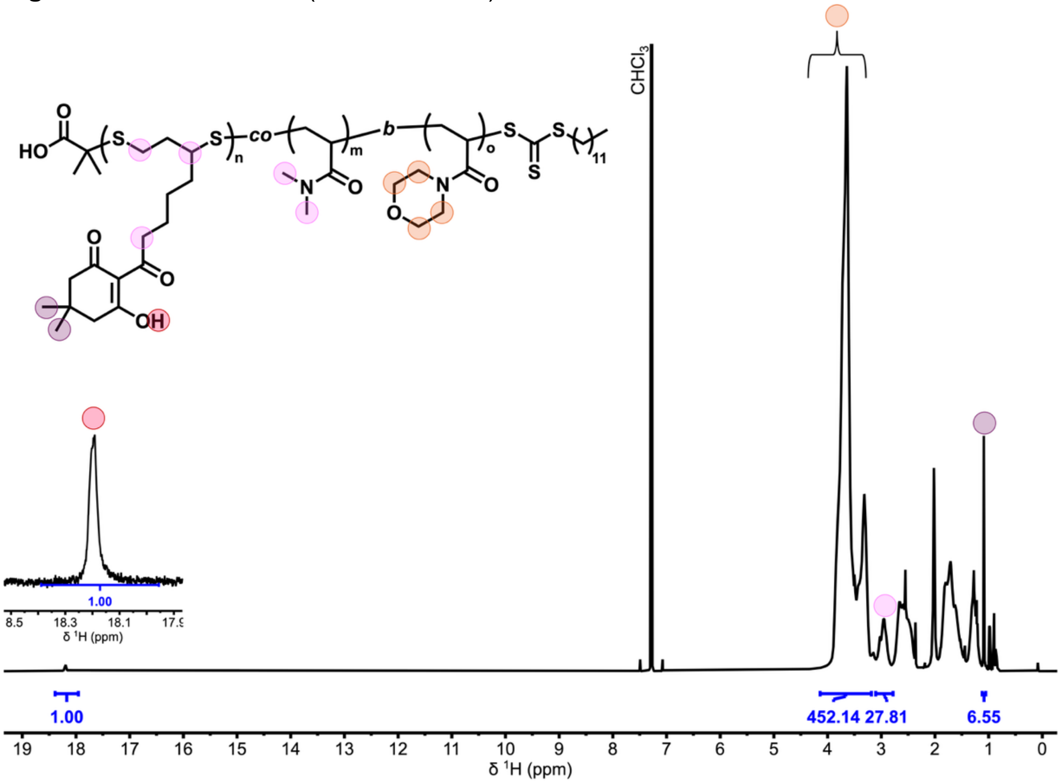

Figure S56. ^{1}H NMR of P(TKLA-*co*-DMA)-*b*-PNAM, recorded in $CDCl_3$.

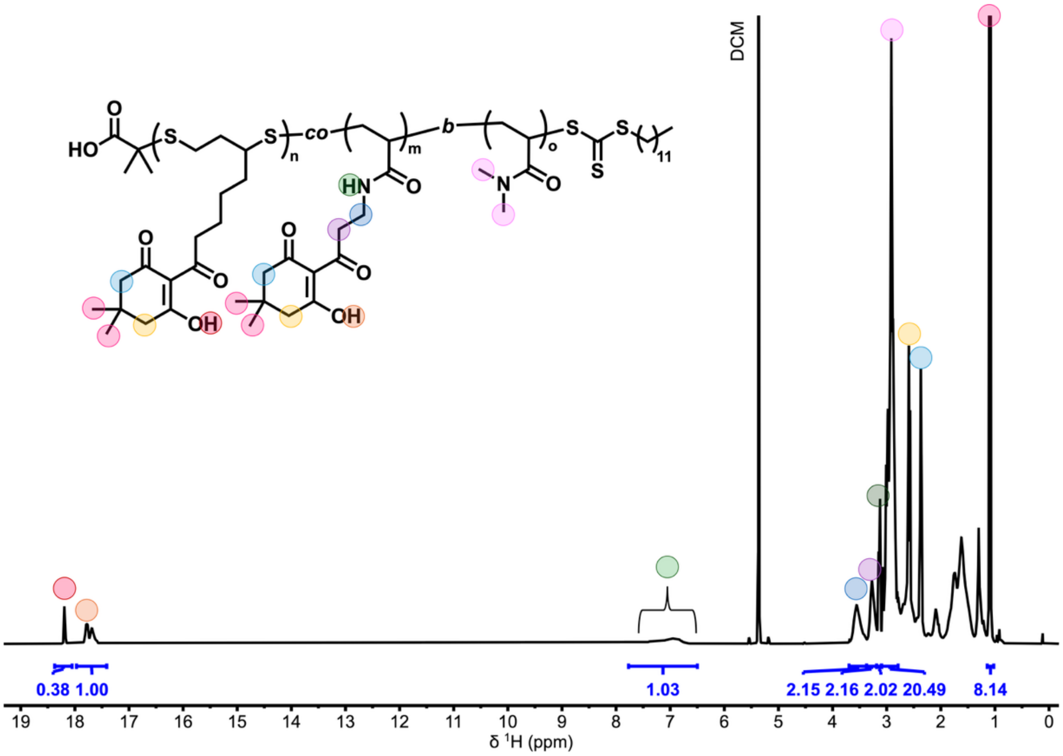

Figure S57. ^{1}H NMR of P(TKLA-*co*-TK2Am)-*b*-PDMA, recorded in DCM-d_2.

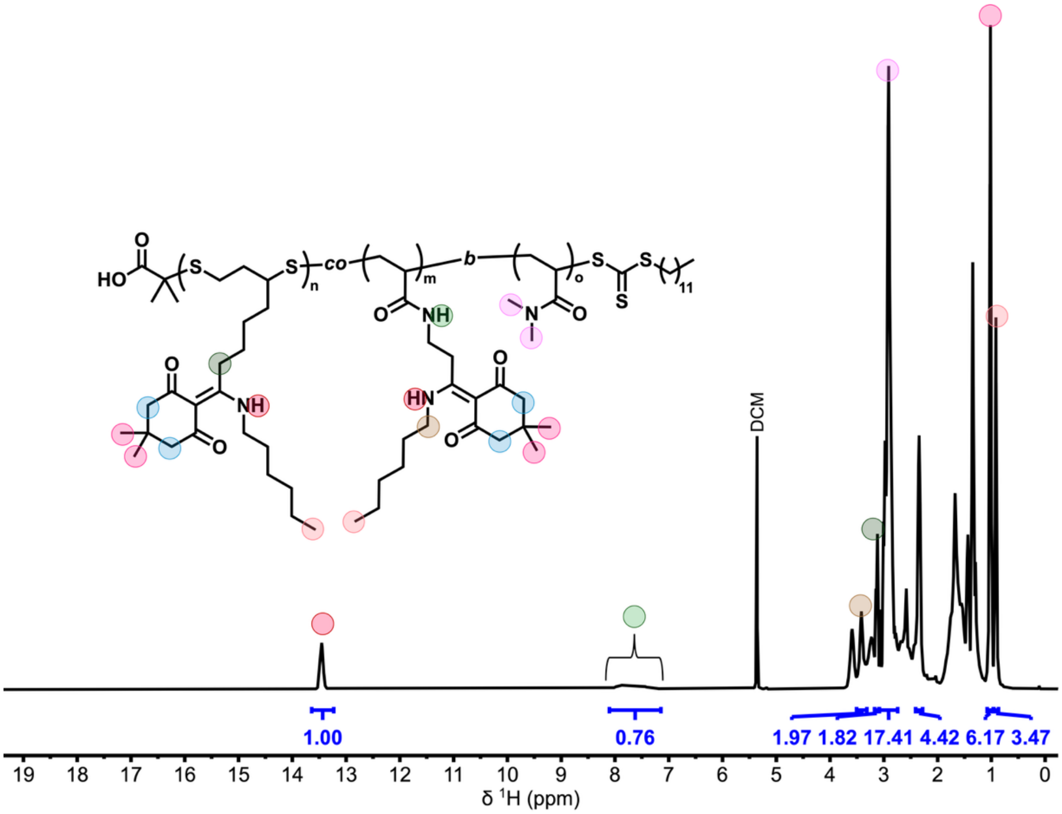

Figure S58. ^{1}H NMR of P(DKE-hexyl)-*b*-PDMA prepared from P(TKLA-*co*-TK2Am)-*b*-PDMA, recorded in DCM-d_2.

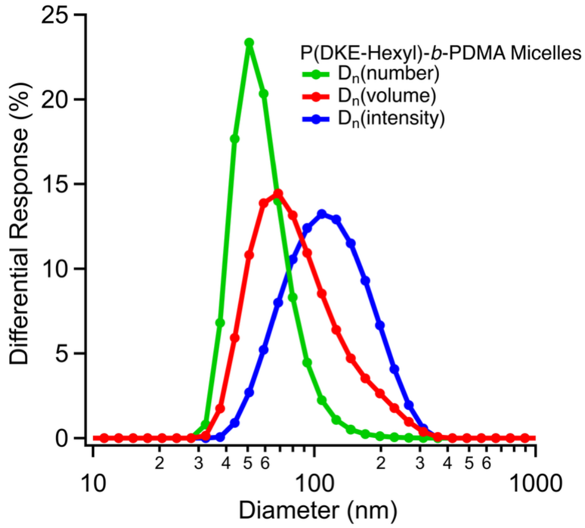

Figure S59. Number-, volume-, and intensity-average hydrodynamic diameters of micelles as measured by dynamic light scattering DLS of self-assembled P(DKE-hexyl)-*b*-PDMA.

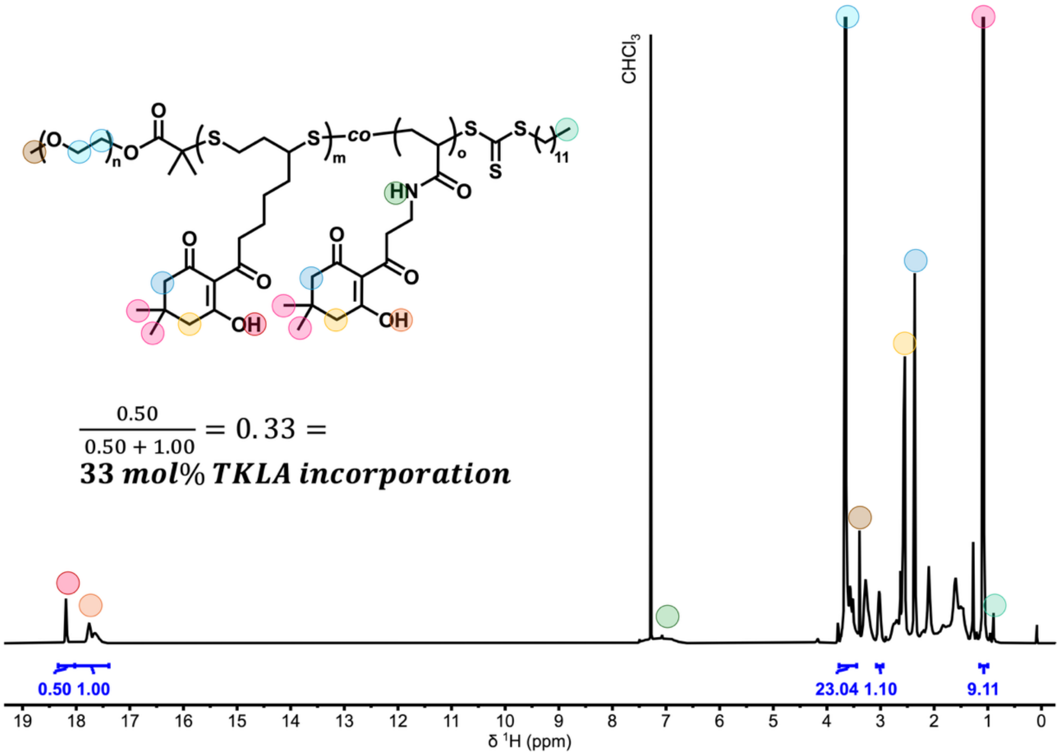

Figure S60. ^{1}H NMR of PEG-*b*-P(TKLA-*co*-TK2Am), recorded in $CDCl_3$.

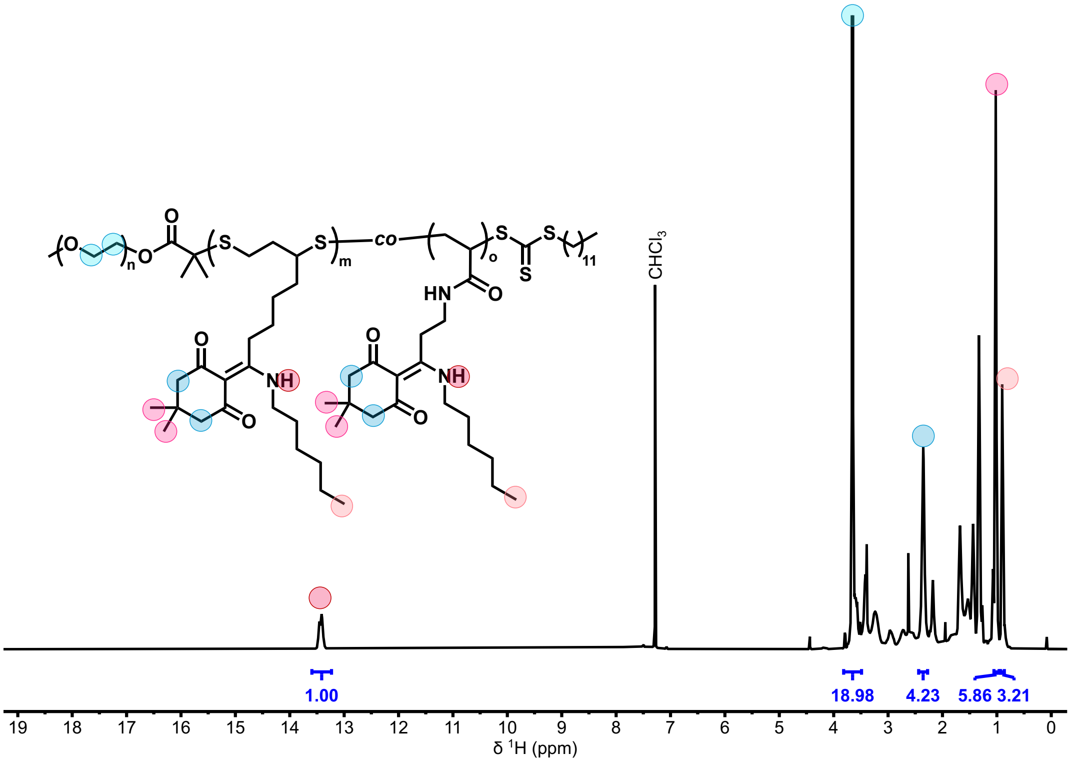

Figure S61. ^{1}H NMR of PEG-*b*-P(DKE-hexyl) prepared from PEG-*b*-P(TKLA-*co*-TK2Am), recorded in $CDCl_3$.

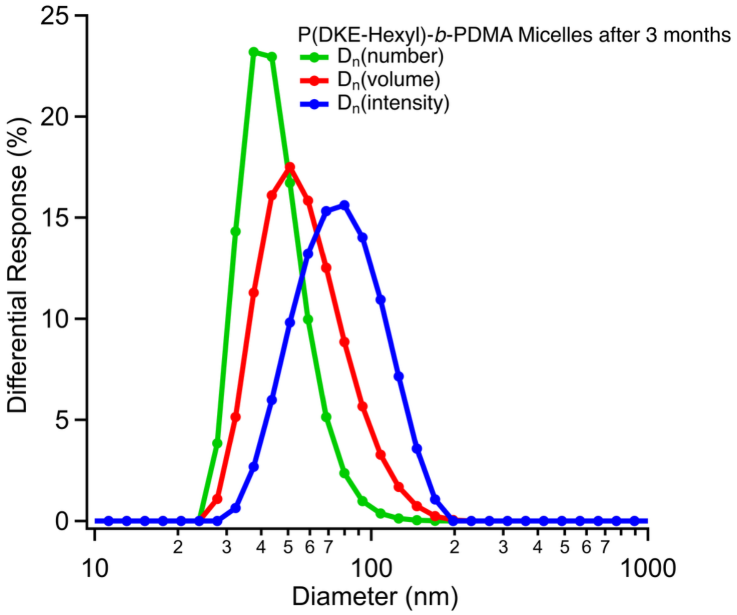

Figure S62. Number-, volume-, and intensity-average hydrodynamic diameters of micelles as measured by dynamic light scattering DLS of self-assembled PEG-*b*-P(DKE-hexyl) after 3 months of storage at rt, showing good colloidal stability.

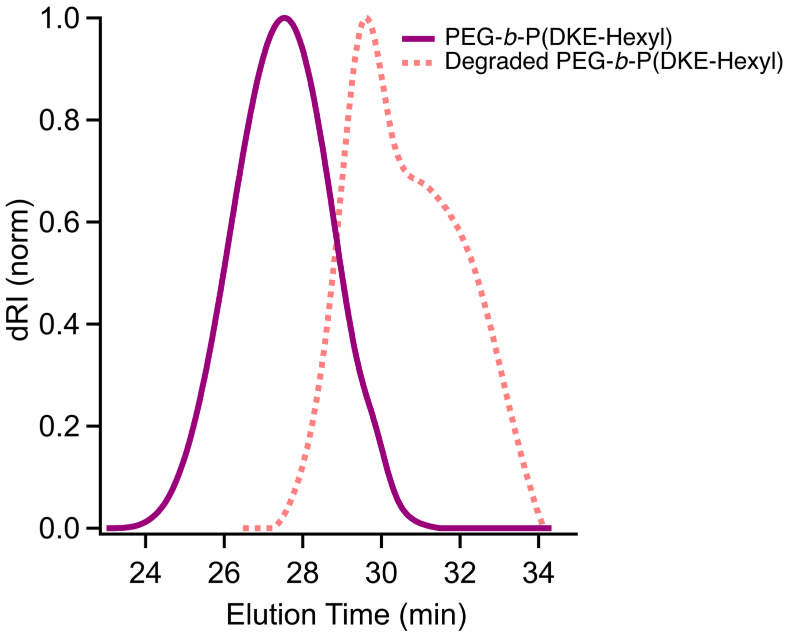

Figure S63. SEC traces corresponding to degradation of PEG-*b*-P(DKE-hexyl) with 100 mM TCEP in water/DMF mixture.

References

(1) Lai, J. T.; Filla, D.; Shea, R. Functional Polymers from Novel Carboxyl-Terminated Trithiocarbonates as Highly Efficient RAFT Agents. *Macromolecules* **2002**, *35* (18), 6754–6756. https://doi.org/10.1021/ma020362m.

(2) Grothe, D. C.; Meyer, W.; Janietz, S. Acrylate Functionalized Tetraalkylammonium Salts with Ionic Liquid Properties. *Molecules* **2012**, *17* (6), 6593–6604. https://doi.org/10.3390/molecules17066593.

(3) Cummings, C. S.; Campbell, A. S.; Baker, S. L.; Carmali, S.; Murata, H.; Russell, A. J. Design of Stomach Acid-Stable and Mucin-Binding Enzyme Polymer Conjugates. *Biomacromolecules* **2017**, *18* (2), 576–586. https://doi.org/10.1021/acs.biomac.6b01723.

(4) Napolitano, A.; d'Ischia, M.; Costantini, C.; Prota, G. A New Oxidation Pathway of the Neurotoxin 6-Aminodopamine. Isolation and Characterisation of a Dimer with a Tetrahydro[3,4a]Iminoethanophenoxazine Ring System. *Tetrahedron* **1992**, *48* (39), 8515–8522. https://doi.org/10.1016/S0040-4020(01)86599-6.
